# Failure Detection Methods for Pipeline Networks: From Acoustic Sensing to Cyber-Physical Systems †

**DOI:** 10.3390/s21154959

**Published:** 2021-07-21

**Authors:** Boon Wong, Julie A. McCann

**Affiliations:** Adaptive Emergent Systems Engineering Group, Department of Computing, Imperial College London, Exhibition Road, London SW7 2AZ, UK; j.mccann@imperial.ac.uk

**Keywords:** pipeline failure detection, non-destructive measurement, acoustic measurement, wireless sensor networks, cyber-physical systems

## Abstract

Pipeline networks have been widely utilised in the transportation of water, natural gases, oil and waste materials efficiently and safely over varying distances with minimal human intervention. In order to optimise the spatial use of the pipeline infrastructure, pipelines are either buried underground, or located in submarine environments. Due to the continuous expansion of pipeline networks in locations that are inaccessible to maintenance personnel, research efforts have been ongoing to introduce and develop reliable detection methods for pipeline failures, such as blockages, leakages, cracks, corrosion and weld defects. In this paper, a taxonomy of existing pipeline failure detection techniques and technologies was created to comparatively analyse their respective advantages, drawbacks and limitations. This effort has effectively illuminated various unaddressed research challenges that are still present among a wide array of the state-of-the-art detection methods that have been employed in various pipeline domains. These challenges include the extension of the lifetime of a pipeline network for the reduction of maintenance costs, and the prevention of disruptive pipeline failures for the minimisation of downtime. Our taxonomy of various pipeline failure detection methods is also presented in the form of a look-up table to illustrate the suitability, key aspects and data or signal processing techniques of each individual method. We have also quantitatively evaluated the industrial relevance and practicality of each of the methods in the taxonomy in terms of their respective deployability, generality and computational cost. The outcome of the evaluation made in the taxonomy will contribute to our future works involving the utilisation of sensor fusion and data-centric frameworks to develop efficient, accurate and reliable failure detection solutions.

## 1. **Introduction**

Pipeline networks are commonly used to transport water, oils and gases over long distances in cities, housing estates and industrial areas. While some pipelines are subject to faults such as weld defects that are caused by a variety of reasons which include poor quality of pipe materials and cracking due to strain [1], most pipelines are openly exposed to environmental conditions, such as rain and floods, and damage due to human error and vandalism, as well as unintended damage due to construction and development activities. For overground pipelines, although structural failures, such as cracks and leakages can be identified visually, often, these failures can only be detected at their critical stages when they become disruptive. For buried, underground and submarine pipelines, where visual inspection is not possible, inspection tools, which are either human-operated [2,3] or automated [4,5], are used.

Human-operated inspection tools are often inefficient since intensive human participation is required in order to inspect relatively long distances of pipelines daily. Therefore, the employment of automated inspection tools has become increasingly popular. Prior to 2010, growing popularity in the use of ultrasonic-based inspection methods was observed, where research in the optimisation of the geometrical design of ultrasonic-phased arrays for guided wave inspection was actively conducted [6,7,8]. Since 2010, there has been a shift of interest from ultrasonic transducers to acoustic emission (AE) sensing methods [9,10,11]. There has also been a growing interest among researchers in inspection methods based on the analysis of hydraulic parameters such as pressure and flow rate [12,13,14]. At the same time, the employment of magnetic flux leakage (MFL) sensing for pipeline failure detection has become increasingly relevant due to its applicability across various types of pipeline failures [15,16,17].

Technologies such as ground-penetrating radar (GPR) [2], infrared thermography [18] and impact echo (IE) [19] are widely employed in the industry, especially in human-operated inspection tools. However, the dimensions, designs and operational requirements of the sensing devices for these technologies have constrained them from being adopted in remote and automated pipeline monitoring systems [20]. In conjunction with the extensive implementation of Industry 4.0 [21,22,23], sensors, such as ultrasonic, acoustic, hydraulic and Hall effect sensors, have been retrofitted in the form of wireless sensor networks in existing pipeline networks [24,25,26]. These sensors are often small in size, inexpensive and can be easily interfaced with embedded systems. These technologies became increasingly relevant with the deployment of autonomous robots for the direct measurement of the magnitudes of defects in pipeline networks [27,28]. The emergence of unmanned aerial vehicles, better known as drones, for the detection of surface defects of pipelines has overcome the limitations of remote monitoring, at the same time reducing the workload required to monitor the integrity of pipelines in large plants [3].

Since 2010, many researchers have been focusing on developing efficient pre-processing and pipeline failure categorisation techniques for data or signals collected from sensors by employing machine learning methods [29,30] suited to embedded platforms. The pre-processing of data or signals using such methods as Kalman filter [13] and wavelet transform algorithms [31] helps to increase the reliability of failure categorisation techniques through the removal of noise and the enhancement of quality. There has also been an increasing interest in the image reconstruction of the in-pipe environment, where detection methods, such as process tomography [32,33], are becoming more mainstream. As of today, many innovative wireless sensor networks for pipeline systems emphasize the failure response time, efficiency of computation and the reliability of the communication systems used [14,34,35,36,37,38,39,40]. By having a combination of physical pipeline networks, sensing capabilities and computational elements, wireless sensor networks for the detection of defects in pipelines are essentially part of the family of cyber-physical systems.

The rest of this paper is structured as follows: Section 2 discusses the working principles, theories and critiques for existing, non-destructive pipeline failure detection methods. In Section 3, the findings from Section 2, the computational cost, deployability and generality of each of the failure detection methods are discussed and summarised. The computational cost takes into account elements such as the types of the algorithms used, the processing mode and the number of sensor nodes. The aspect of deployability of an inspection method considers the intensity of the effort needed to implement the method in industrial pipeline networks. The generality of an inspection method is measured based on the level of human intervention required to adjust the hardware specifications and software parameters to cater to pipeline networks of various operating environments.

## 2. **Pipeline Failure Detection Methods**

A failure or defect in a pipeline can exist generally in the form of a crack, a blockage, a leakage, a weld defect or corrosion. Cracks and leakages in pipelines may be caused by mechanical stress, pressure and prolonged thinning of the pipeline due to corrosion. Blockages in pipelines are normally caused by oversized loads or the build-up of sediments. Corrosion, which is related to the ageing of pipelines, is induced by the oxidation of the metallic wall of the pipeline and friction between the transported load and the inner wall of the pipeline, as well as the corrosive nature of the load. Weld defects at pipeline joints are attributed to poor welding jobs and mechanical damage due to fluid pressure and ambient stress. In order to avoid the occurrence of disruptive failures, the early detection of pipeline defects is necessary [41].

Table 1, in the form of a look-up table, shows various existing non-destructive methods along with their suitability for the detection of different pipeline defects. The key aspects and common data or signal processing techniques for each of the methods are also enumerated in the same table. The failure detection methods covered in this paper are non-exhaustive and are, to the best of our knowledge at the point of writing, include non-destructive technologies that have been practically validated in the industry either in the form of modern wireless sensor networks or human-operated devices.

### 2.1. Acoustic Reflectometry (Blockage, Leakage, Crack, Corrosion, Weld Defect)

The use of acoustic reflectometry is a dated approach in the pipeline inspection industry, especially in the detection of physical blockages and mechanical flaws, such as leakages and cracks. Acoustic reflectometry employs the law of reflection and time-of-flight technique for the computation of the position and the size of a foreign object in a pipeline. The time-of-flight (TOF) technique is used to resolve the distance between the source of the wave and an object, acting as an obstruction, by measuring the round trip time of the wave. Since the law of reflection and time-of-flight technique are used, reflectometry methods perform best in straight pipelines when the line-of-sight is present between the acoustic–ultrasonic sources and the objects of interest. The ease of the TOF technique makes the reflectometry methods more cost-effective, computationally.

**Figure 1:**
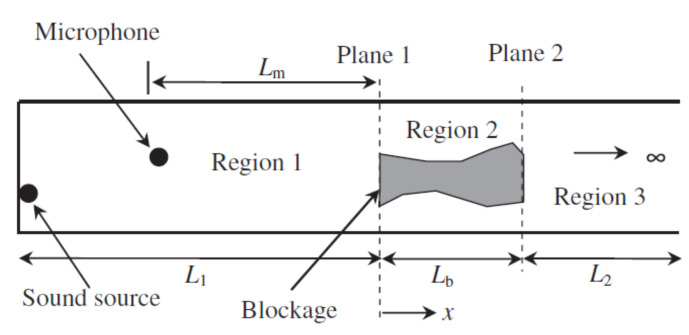
The placement of sound source and microphone in a pipeline for reflectometry test. Reprinted from Applied Acoustics, Volume 87, Duan, W.; Kirby, R.; Prisutova, J.; Horoshenkov, K.V. On the Use of Power Reflection Ratio and Phase Change to Determine the Geometry of a Blockage in a Pipe, Page 190–197, Year 2015 with permission from Elsevier [43].

The setup for a reflectometry test is also relatively simple and replicable for different pipeline scenarios since common portable testing devices, such as loudspeakers, ultrasound transducers, microphones and signal analysers, are generally used. Being common and well developed, the testing devices are also mostly affordable and can be repeatedly used with minimal maintenance effort. However, the deployability of reflectometry methods in the form of wireless sensor networks is limited by the bulkiness of these devices. This limitation affects the scalability of reflectometry approach adversely since a larger amount of human intervention is required as the capacity of a pipeline plant increases. An example of the placement of these devices in a pipeline is shown in Figure 1.

In reflectometry, an audible sound or ultrasonic wave, in the form a small signal pulse, is injected into the pipeline, using pulse generators to remotely detect obstructions, such as blockages, and pipeline components, such as valves, flanges and T-sections, along the length of the pipeline [44]. In the presence of an obstruction in the path of the transmitted pulse, a portion of the pulse is reflected towards the acoustic source. The reflected pulse is measured and analysed in the time and frequency domains to obtain an estimation of the location and the size of the blockage [42].

In [42], audible sound waves were injected into the pipeline, using a loudspeaker that was driven by an acoustic pulse generator. The reflection response of the waves was subsequently measured using a microphone. The reflected signal can be observed and analysed on an oscilloscope. Tests were conducted on a pipe with varying blockage areas and varying blockage lengths, respectively. The acoustic response for both tests was analysed in the frequency domain in order to obtain a more pronounced differentiation of the amplitudes of the signals for different blockage conditions.

The method in [42] does not provide the absolute dimensions of the blockages since the evaluation of sizes is based on the comparison between the acoustic response captured from different blockage scenarios. Since no direct measurements are involved, the understanding about the blockage condition is mostly qualitative or, at best, indirectly quantitative. There is also a need for an offline data analysis before the blockage condition can be identified. Unlike that in [42], which relied solely on the comparison of the amplitudes and centre frequencies of the reflected acoustic signals between blockages of different sizes, the method proposed in [43] employed the power reflection ratio (ratio of square of the respective amplitude for incident and reflected acoustic waves) and the phase change between the incident and reflected acoustic waves to determine the geometry of the blockage in the form of the area ratio and length.

The power reflection ratio (∧), containing blockage length ($L_{b}$), area ratio (σ), wave number of air (k=ω/c), radian frequency (ω) and speed of light (c), is expressed in [43] as follows:

(1) $$\wedge =\frac{{\left(\sigma^{2}-1\right)}^{2}}{{\left(\sigma^{2}+1\right)}^{2}+4\sigma^{2}\cot^{2}kL_{b}}$$

The phase difference (ψ) is expressed in [43] as follows:

(2) $$\psi =\frac{2\sigma \cot kL_{b}}{\sigma^{2}+1}$$

By rearranging Equations (1) and (2), the area ratio (σ) and blockage length ($L_{b}$) can be solved separately as follows:

(3) $$\sigma^{2}=\frac{\wedge \left(1+\psi^{2}\right)+1\pm 2\sqrt{\wedge (1+\psi^{2})}}{1-\wedge (1+\psi^{2})}$$

(4) $$L_{b}=\frac{1}{k}\cot^{-1}\left[\frac{(\sigma^{2}+1)\psi }{2\sigma }\right]+\frac{n\pi }{k},\;n=0,1,2,\cdots$$

Equation (4) primarily has an infinite of solutions due to the *n* number of frequencies. However, by having the frequencies specified along with the identification of the area ratio σ via Equation (3), a finite number of solutions for blockage length $L_{b}$ can be obtained. Percentage errors of less than 20% were obtained for the area ratio of a blockage when its cross-sectional area was greater than 5% of the pipe cross-section. For the blockage length, predictions were within 30% of the actual values. While the method in [42] relied on the backward analysis to understand the reflection responses of different blockage sizes, the method proposed in [43] was able to quantitatively identify the length and area ratio of the blockage based on the differences between the properties of the incident and reflected acoustic waves.

The acoustic reflectometry method in [44], mentioned previously for the detection of blockage, was also used in the detection of leakages in pipelines. The presence of a hole in a pipe is detected through the identification of the difference between the reference reflected signal signature and the test reflected signal signature. The test signature is obtained experimentally from a faulty pipe, while the reference signature is obtained from a normal pipe with similar material type and dimensions. In the test reflected signal, reflected components from the welded joints of the pipeline network were identified. Although this would allow the locations of the welded joints to be identified, no further steps were taken in [44] to detect defective welds since the granularity of the reflected components was insufficient for further analysis.

Audible sound waves in the kilohertz frequency range, having wavelengths between 17 mm and 17 m, are effective for the detection of moderate to large blockages [42] but are often limited by the lack of granularity for the inspection of small defects, such as cracks, leakages, gradual thinning, pitting defects and weld defects. Vos and As [46] conducted a case study to investigate the effectiveness of acoustic resonance technology (ART) for the measurement of the thickness of the wall of a pipeline, using ultrasonic waves. ART improved upon the standard ultrasonic free-space time-of-flight technology. The standard ultrasonic technology uses single-frequency narrow-band waves between 5 MHz and 10 MHz that are prone to data loss in the presence of impurities or irregularities on the surface of the wall of a pipeline. At these megahertz frequencies, an echo of a higher amplitude can be detected since the energy of the incident wave is high. However, one trade-off is the limited range between the transducer and the wall due to the short micrometre wavelengths of the ultrasonic waves.

**Figure 2:**
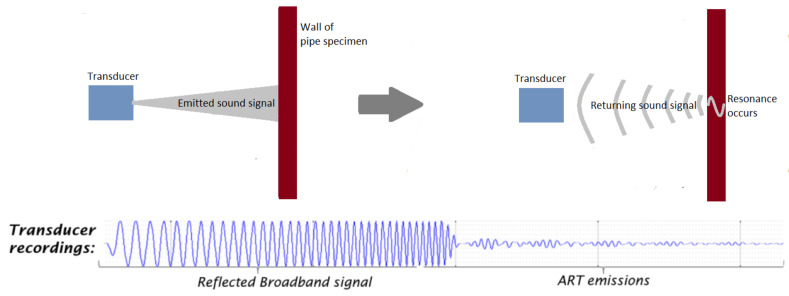
The operating principle of the acoustic resonance technology. Modified from [46].

In ART, a wide-band ultrasound signal, consisting of constituent signals of lower frequencies ranging from 400 kHz to 1.2 MHz, is transmitted by an ultrasonic transducer towards the wall of a pipeline. At the wall, part of the signal is reflected, while the remaining signal is transmitted through the wall. The signal that is transmitted through the wall is subsequently compressed by the high-density interfaces within the wall, resulting in the resonance of the transmitted signal, which varies in terms of the peak amplitudes of the resonant frequencies depending on the thickness of the wall. The resonance continues for a period, usually longer than the time-of-flight of the ultrasonic signal. This resonant signal is eventually emitted by the wall to the transducer and is detected as tone bursts of smaller amplitudes, compared to those of the ultrasound signal that was reflected earlier by the wall. Figure 2 summarises the operating principle of ART. By measuring the amplitudes of the resonance and their respective frequencies for different thicknesses of the wall of a pipeline as well as their respective frequencies, the thickness of the wall for a random pipeline can be derived using data interpolation with estimated accuracies of ±0.2 mm.

Although acoustic reflectometry can be performed with a relatively simple setup, it is, however, invasive since the test requires access to the internal environment of a pipeline. At the same time, while the test is ongoing, the operation of the pipeline network needs to be halted. Due to the limited range of the acoustic wave and the presence of multiple intersections in a pipeline network, intensive human intervention is also required to carry out a large number of tests to inspect all the pipelines thoroughly. However, acoustic reflectometry can be used in various types of pipelines since air is used as the propagation medium.

The acoustic reflectometry technology in the form of an ultrasonic phased array was used in [6,7,8,45] for the detection of weld defects. Weld defects are irregularities at the welding point joining two different pipelines. These irregularities are often due to corrosion at the welding points, mechanical damage due to excessive vibrations, and poor welding skills. Cracks in the wall of a pipeline are difficult to detect since these defects often exist in the form of discontinuities that contribute to only minor changes in the surface roughness and irregularities. Weld defects, on the other hand, due to the bulging nature and relatively larger surface area of individual welds compared to cracks, contribute to changes in the surface roughness at the welding points. Therefore, ultrasonic waves that have sub-centimetres wavelengths are suitable to detect and localise weld defects in a pipeline network.

Traditional ultrasonic testing systems are based on a single transducer unit, which is only capable of producing a single ultrasonic beam that is unable to diffuse through a solid medium efficiently, due to its dispersed nature. Another weakness of these traditional systems is their fixed focus, which limits their application in the inspection girth weld at the welding point between two pipelines. The inspection of a girth weld requires various angles of focus to ensure that all layers of the weld can be inspected.

**Figure 3:**
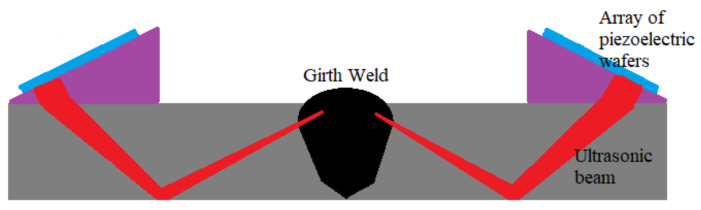
The schematic model of an ultrasonic phased array. Modified from [45].

The limitations of traditional ultrasonic testing systems are overcome using an ultrasonic phased array, consisting of an array of wafers as shown in Figure 3. Each of the wafers can be electronically steered to change the transmission angle of their respective ultrasonic beams. A delay of the transmission of the ultrasonic beam from each of these wafers allows these beams to superimpose based on Huygen’s principle to produce a resultant inspection beam that has higher energy level and directivity. By controlling the delay of the transmission of the ultrasonic beams from individual wafers of a phased array, the side lobes of the resultant inspection beam can be reduced to increase the magnitude and directivity of the central lobe. In [7,8], the authors from [45] optimised the number of wafers in an ultrasonic phased array by analysing the directivity of the superimposed ultrasonic beams in terms of the amplitudes of both the main lobe and the side lobes. It was found that the amplitude of the main lobe increases, while the amplitudes of the side lobes decrease as the number of wafers increases. The result therefore indicated that the performance of inspection increases as the number of wafers increases. However, it is important to note that the complexity of the channels of the acquisition system increases as the number of ultrasonic beams generated by the phased array increases.

However, the conventional ultrasonic phased array, despite the pronounced improvement in performance, compared to the traditional ultrasonic inspection systems, is only able to generate a two-dimensional image of the girth weld of a pipeline since the inspection is performed from a stationary standpoint. In order to overcome this limitation, Ref. [6] employed the synthetic aperture radar (SAR) technique to produce a three-dimensional image, which has a higher resolution, compared to the two-dimensional image produced by the conventional phased array methods. The image reconstruction scheme of the SAR technique involves several instances of the transmission of an ultrasonic beam towards the target object as the transducer moves along the longitudinal axis of the pipeline. At each instance of transmission, the two-dimensional information from the echo received by the transducer is used to construct a three-dimensional image of the target object.

Ultrasonic reflectometry involves the use of signal processing techniques to remove noise and perform frequency analysis on the echoes produced by the transmitted ultrasonic beams, which are reflected.However, since weld defects are relatively larger than cracks, the construction of the image of a girth weld can be performed by identifying the dimensions of the weld based on the fundamental time of flight principle. The time taken for one round trip for the inspection beam varies proportionally with the change in the width of the girth weld at the different layers of inspection. The inspection system for weld defects based on ultrasonic phased arrays is only suitable to monitor conditions of the weld in a localised manner since only a maximum of two welding points (one welding point at each end of a pipeline) can be inspected by a single phased array. Depending on the length of a pipeline, which determines the distance between two welding points, the ultrasonic phased array may only be able to serve one welding point, due to the limited propagation range of the ultrasonic waves.

### 2.2. Guided Wave Inspection (Blockage, Leakage, Crack, Corrosion)

**Figure 4:**
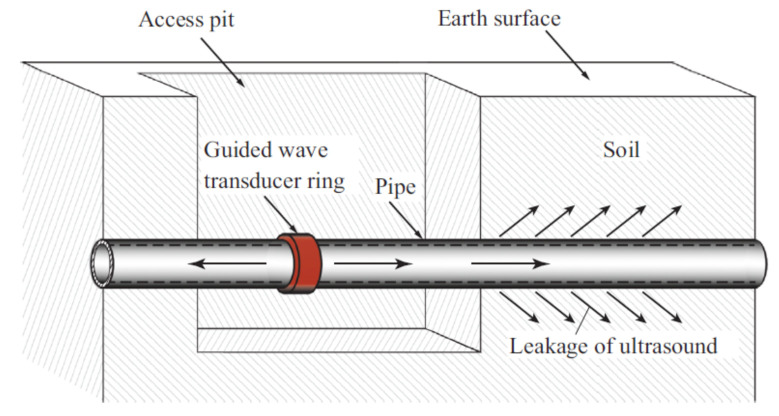
An ultrasonic transducer ring mounted onto an excavated pipeline to perform guided wave inspection. Reprinted under Creative Commons CC BY license [48].

Ultrasonic waves are commonly used in guided wave inspection since ultrasound can be transmitted in a narrower beam, compared to that of audible sound, due to its significantly shorter wavelength. The narrow beam property allows ultrasound to be propagated more efficiently in thin solid media, such as the wall of a pipeline, for a distance of up to 50 m [48]. The wide-area scanning capability of ultrasonic waves can be significantly enhanced, using a guided wave transducer ring as shown in Figure 4. While the cost required to either acquire or build the ultrasonic transducer ring is still far from being economical due to the precision of the manufacturing aspects required, the cost of computation required to analyse the reflected signals captured by the ultrasonic transducer is also expensive, due to the high level of accuracy and resolution in signal processing needed. Often, a signal or spectrum analyser is used to overcome the computational needs.

However, unless the guided wave methods are implemented in the form of wireless sensor networks along with embedded signal analysers with smaller form factors, these methods have low scalability and are not favourable in scenarios where human intervention has to be minimized. As mentioned previously, the sensing range and the propagation characteristics of ultrasonic waves vary with the types of building materials and the dimensions of the pipelines. The selection of the optimal centre frequency and the range of frequencies should, therefore, be tuned to suit the characteristics of the pipelines for optimal sensing performance.

In [47], a guided ultrasonic wave inspection was used as a non-intrusive means to detect and characterise blockages in a pipeline. A frequency sweep was performed to detect the frequencies with the highest reflection coefficients which correspond to the cut-off frequencies of the torsional modes of the reflected ultrasonic wave. By studying the reflection coefficients of the reflected signal and the degree of dispersion of the transmitted signal using a spectrogram, the bonding condition of the blockage in a pipeline was able to be determined. Time-series cross-correlation between the incident and the reflected signals was also performed to localise the position of the blockage in the pipeline. Being non-intrusive, guided ultrasonic wave inspection is a suitable means for the detection of blockages in both overground and underground pipelines. However, pipelines with different diameters require transducer rings of different sizes. The ease of retrofitting the inspection system to an existing pipeline network largely depends on the operational environment and maintenance access. For an underground pipeline network, the pipeline is excavated as shown in Figure 4 to reveal the mounting point for the ultrasonic transducer ring. The number of excavation exercises depends on the effective range of the ultrasonic waves, which is affected by such factors as soil conditions, pipeline materials and pipeline dimensions.

In a guided ultrasonic wave inspection, the ultrasonic waves propagate in the walls of the pipelines. Therefore, the performance of the inspection in terms of the sensing range and signal fidelity is affected by the type of pipeline materials and wall thickness. For instance, the attenuation coefficient of steel and that of polyvinyl chloride (PVC) are different. The degree of attenuation is dependent on the frequency of the ultrasonic waves as well as the attenuation coefficient of the propagation media [9]. In order to achieve optimal inspection performance, it is necessary to tune the frequency of the ultrasonic wave based on the type and dimensions of the propagation medium.

Conventionally, an excavation exercise is necessary every time a pipeline requires inspection since the transducer ring is only mounted during the period of inspection. The guided wave inspection method also requires maintenance engineers to access data continuously, using a computer and a signal analyser during the inspection process. Human intervention in the guided wave inspection can be reduced significantly if a distributed wireless sensor network with inspection chambers built at strategic locations for sensors is installed permanently.

Ultrasonic waves, which have shorter wavelengths (less than 1.9 cm) and higher frequencies (more than 20 kHz), compared to audible sound waves, which have wavelengths longer than 1.9 cm and frequencies between 20 Hz and 20 kHz, are able to detect smaller objects more efficiently. However, the area coverage of ultrasonic reflectometry is limited by the shorter wavelengths of the ultrasonic waves. Therefore, since wide-area scanning is required for the detection of blockages, the use of audible sound waves would be more favourable, compared to ultrasonic waves, due to their longer wavelengths.

Ultrasonic waves have shorter wavelengths, which are predominantly more effective than acoustic waves for the detection of small discontinuities on the wall of a pipeline. Ultrasonic reflectometry or scanning has, therefore, been a more popular research subject in the domain of leakage and crack detection. In [49], a line-focusing ultrasonic array in the form of multiple rings with sensors spaced with no overlaps on each ring, was employed to detect crack-like flaws, metal loss and dents in pipelines. Two-dimensional images of the flaws in the pipeline were reconstructed, using amplitude-scan (A-scan) ultrasonic signals captured by the array, after which the sizes and the locations of the flaws could be obtained. An improvement to the line-focusing array was in the form of a guided wave transducer ring as shown earlier in Figure 4. However, the ultrasonic techniques utilised by [47,49] relied on conventional methods that are based on principle of passive scattering, which makes use of the amplitude and phase variations between the transmitted and reflected signals in the linear elastic region of the characteristic profile of the ultrasonic wave [52]. The sensitivity of these conventional methods is limited to the wavelength of the ultrasonic wave transmitted by the transducer in the inspection system.

The drawback in the limited sensitivity can be addressed using non-linear ultrasonic techniques, which study the non-linear ultrasonic behaviour, such as higher harmonic generation, sub-harmonic generation, non-linear resonance and mixed frequency response. Higher harmonic generation occurs when the waveform of an incident wave is distorted by the non-linear elastic response of a target object, resulting in the generation of higher harmonics in the frequency spectrum alongside the fundamental frequency component. Lower harmonic generation occurs as the result of two overlapping adjacent ultrasonic waves of different amplitudes in the presence of a crack closure phenomenon, where two opposite faces of the crack remain in contact. Non-linear resonance happens when a target object that has a poorly defined geometry, such as a micro-crack, is excited using an ultrasonic wave. Mix frequency response is the vibro-modulation effect experienced by a high frequency ultrasonic wave when a low frequency vibration is used to induce compression and dilation phenomena on a defect [52].

**Figure 5:**
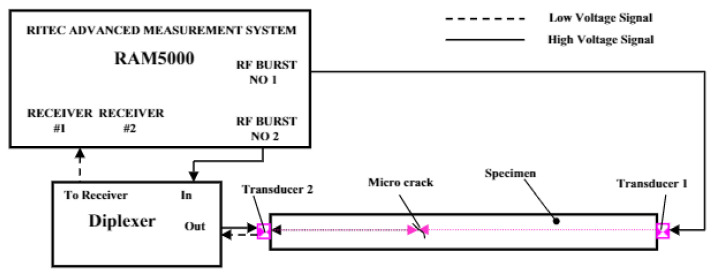
Schematic diagram of the experimental setup for collinear wave mixing. Reprinted from NDT & E International, Volume 62, Jiao, J.; Sun, J.; Song, G.; Wu, B.; He, C. Micro-crack Detection Using a Collinear Wave Mixing Technique, Page 122–129, Year 2014 with permission from Elsevier [54].

**Figure 6:**
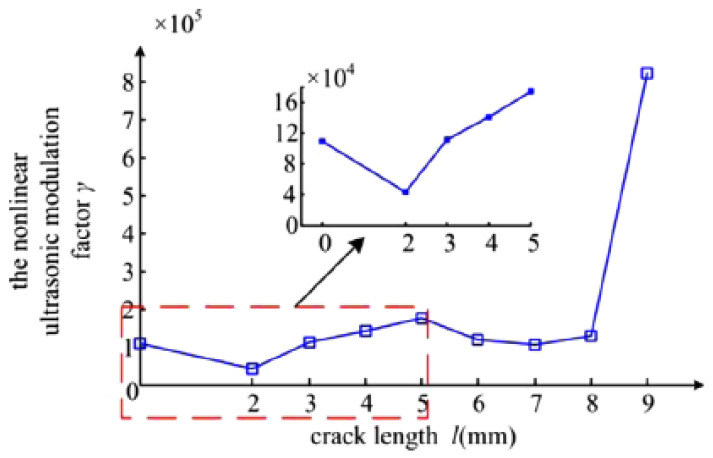
The relationship between the non-linear ultrasonic modulation factor and the crack length.Reprinted from NDT & E International, Volume 79, Li, N.; Sun, J.; Jiao, J.; Wu, B.; He, C. Quantitative Evaluation of Micro-cracks Using Non-linear Ultrasonic Modulation Method, Page 63–72, Year 2016 with permission from Elsevier [53].

The non-linear behaviour of the ultrasonic wave was explored in [53] to quantitatively evaluate the dimensions of micro-cracks through the computation of the non-linear ultrasonic modulation factor from which the length of a micro-crack can be estimated. As shown in Figure 5, an excitation signal consisting of two driving frequencies f_1_ and f_2_ are injected using Transducers 1 and 2, respectively. The transmitted signal is then received using Transducer 2 after which a spectral analysis is conducted to obtain the bispectrum values attributing to the f_1_ + f_2_ and f_1_ − f_2_ components. The bispectrum values are used to calculate the non-linear ultrasonic modulation factor, which correlates with the length of the target crack in the experiment as shown in Figure 6. However, the results produced in [53] as shown in Figure 6 show that between the modulation factor and the crack length, there is an approximate linear relationship when the length is less than 2 mm and more than 5 mm. Beyond 5 mm, the modulation factor becomes highly irregular, due to the changes in the interaction between the ultrasonic waves and the crack.

The use of guided-wave high-frequency ultrasonic scanning for the detection of corrosion in pipelines was discussed in [50]. Low-frequency ultrasonic reflectometry, which has a larger area coverage compared to its high-frequency counterpart due to the long wavelengths of the ultrasonic wave, are generally effective in detecting and locating faults. However, at inaccessible locations, such as pipe supports and T-joints, the detectability of small, sharp and gradual defects using low-frequency ultrasonic reflectometry is low, due the long wavelengths of the ultrasonic waves that are sensitive to the presence of the large features of the pipe supports or joints. The reflections produced by larger features make the detection of smaller features challenging and almost impossible. Therefore, in ultrasonic scanning, the selection of the frequency of the ultrasonic waves determines the effective range of the sizes of defects for which the detectability is high. The frequencies of the ultrasonic waves that are commonly used in guided-wave ultrasonic scanning are as follows:

- S0 mode Lamb wave at ∼1.5 MHz-mm.
- SH0 and SH1 modes at ∼3 MHz-mm.
- Creeping Head-wave Inspection Method at ∼20 MHz-mm.
- Multi-skip (M-skip) at ∼20 MHz-mm.
- Higher-order mode cluster (HOMC) A1 mode at ∼18 MHz-mm.

**Figure 7:**
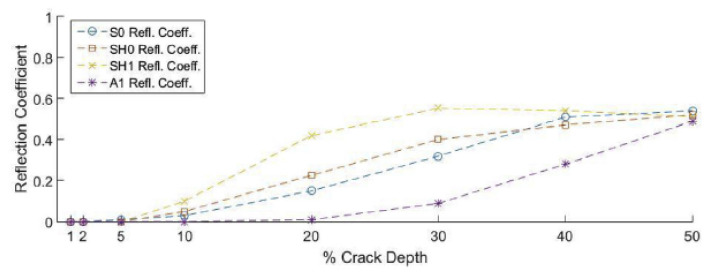
The predicted reflection coefficients of the S0, SH0, SH1 and A1 modes at varying crack depths on a 10 mm thick pipeline using the 2D finite element method. Reprinted under Creative Commons CC BY license [50].

**Figure 8:**
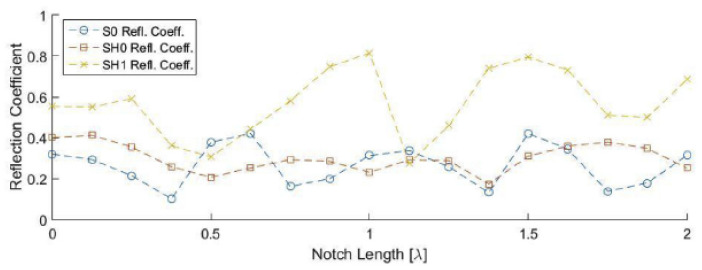
The predicted reflection coefficients of the S0, SH0 and SH1 modes at varying notch lengths and a fixed 30% notch depth on a 10 mm thick pipeline, using the 2D finite element method. Reprinted under Creative Commons CC BY license [50].

The transmitted and reflected waves from the ultrasonic scanning of the wall of a pipeline were analysed to compute the transmission and reflection coefficients. The amplitudes of the coefficients determine whether the modes are suitable for the evaluation of a particular type of defect. Figure 7 shows the predicted reflection coefficients of various guided-wave modes at varying crack depths on a 10 mm thick pipeline, using the 2D finite element method. Based on the results in Figure 7, the SH1 mode produces the highest reflection coefficients among others at crack depths below 30%. At crack depths below 30%, the reflection coefficients for the A1 mode, however, remains insensitive to the changes in depth. At crack depths above 30%, while the reflection coefficients of the S0, SH0 and SH1 modes remain almost constant, those of the A1 mode are observed to be sensitive to the changes in depth. Figure 8 shows the predicted reflection coefficients of various guided-wave modes at varying notch lengths and a fixed 30% notch depth on a 10 mm thick pipeline, using the 2D finite element method. Based on Figure 8, it can be observed that the reflection coefficients of the S0 mode remain insensitive to the changes in the notch length. The reflection coefficients of the SH0 and SH1 mode are sensitive to the changes in the notch length. The reflection coefficient of the SH0 mode portrays a cyclical pattern with the peaks corresponding to the 0.5, 1.0, 1.5 and 2.0 λ notch lengths, whereas those of the SH1 mode show a significant increase at 1.0, 1.5 and 2.0 λ notch lengths [48].

The results in Figure 7 and Figure 8 provide theoretical evidence that the A1 mode was most effective for detecting severe thickness loss (>30%) defects, such as deep and sharp pitting defects, while the SH modes, especially the SH1 mode, was suitable for detecting shallow defects and wide-area gradual thinning. This evidence is also supported by the subsequent practical validation in [50].

In ultrasonic scanning, apart from the computation steps involved in the calculation of the reflection and transmission coefficients, the presence of corrosion defects is determined mainly by a comparative analysis of the amplitudes of the reflection and transmission coefficients. This form of detection provides qualitative, but not quantitative, characterisation of the defects. For example, the inspection is only able to determine whether a defect is present and also the approximate severity of the defect. The challenge to address here is to quantitatively characterise the severity of a corrosion defect to provide useful inputs for predictive and preventive maintenance. Since the guided-wave ultrasonic method is widely used for the inspection of pipelines [48], the scalability remains a minor issue to be addressed despite the need to excavate a pipeline before it can be inspected. However, it was also claimed in [48] that the number of excavations can be minimised by maximising the range covered by each guided-wave transducer ring. In [161], the inspection range for guided-wave inspection varied from several metres for the testing of composite materials to about 100 m for the testing of pipelines. However, the inspection range is often limited by a variety of attenuating factors, such as the conditions of the soil in terms of water saturation, compaction and burial depths.

Guided microwave inspection, unlike the guided ultrasonic wave inspection that uses the wall of a pipeline as the medium of transmission, uses air in the pipeline instead. In [51], microwave signals were generated to propagate in the pipelines. The reflected microwave signals were measured, using a microwave network analyser. By analysing the changes in the resonant frequency between the original and reflected microwave signals, the reduction in the thickness of the wall of a pipeline was able to be determined with evaluation errors smaller than 0.5% of the diameter of the pipeline. The change in the wavelength experienced by the microwave signal caused by the abrupt change in the thickness of the wall of the pipeline results in the shift of wave phase that is responsible for the change in the resonant frequency of the microwave signal.

The experimental results of frequency sweeping conducted in [51] portrayed how the amplitude vs. frequency plots peak at different frequency values for different wall thicknesses of a pipeline. The frequency values at which the peaks occur correspond to the resonant frequencies for each thickness value. This relationship allows the changes in the thickness to be evaluated.

Like the guided ultrasonic wave inspection, the guided microwave inspection relies on the use of a signal analyser to identify changes in the thickness of the wall of a pipeline. Before the quantitative evaluation of the reduction in the thickness can be performed, a resonant frequency versus thickness reduction plot has to first be generated by running an exhaustive set of experiments to obtain the corresponding resonant frequencies for a defined range of thickness reductions. Based on the resonant frequency measured from any random pipelines that are of a similar type as that used in the generation of the plot, the thickness reductions for these random pipelines can be easily determined by searching for the corresponding thickness in the plot. While the computational steps involved in guided microwave inspection are relatively simple, the reliance on the use of signal analysers necessitates the involvement of human intervention, which limits the deployability of the inspection system.

### 2.3. Ultrasonic Gauging (Corrosion, Weld Defect)

**Figure 9:**
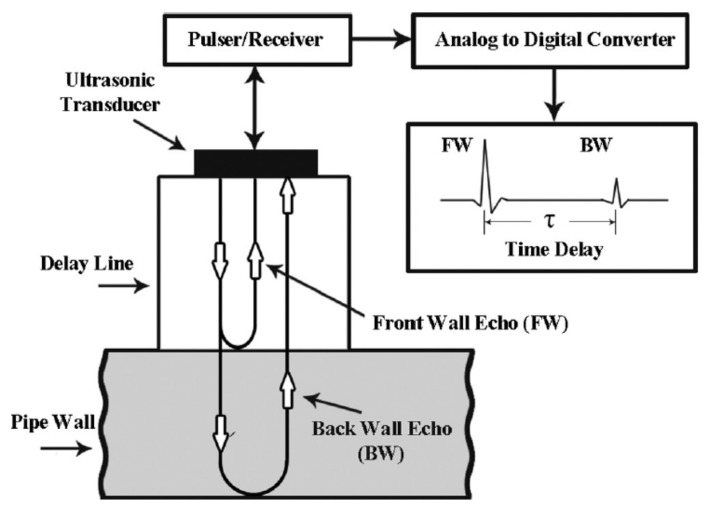
The working principle of an ultrasonic gauge. Reprinted from Ultrasonics, Volume 53, Honarvar, F.; Salehi, F.; Sasfavi, V.; Mokhtari, A.; Sinclair, A.N. Ultrasonic Monitoring of Erosion/Corrosion Thinning Rates in Industrial Piping Systems, Page 1251–1258, Year 2013 with permission from Elsevier [55].

Ultrasonic gauging, in general, is a form of inspection that measures the thickness of the wall of a pipeline based on the difference in the time of flight of the ultrasonic wave from the transducer to the front wall and that to the back wall [55]. Figure 9 shows the working principle of an ultrasonic gauge. Based on Figure 9, the thinning rate of the wall of a pipeline over a time period Δt is expressed as follows:

(5) $$\mathrm{Thinning}\;\mathrm{rate}=\frac{\Delta \tau c_{t}}{2\Delta t}$$

where Δτ= the time delay between the echoes of the front wall and the back wall, and $c_{t}=$ the longitudinal velocity of the ultrasonic wave in the wall of the pipeline. Another form of ultrasonic gauging technique was proposed in [58], which is equivalent to the acoustic resonance technology mentioned in the acoustic reflectometry section, involving the transmission of ultrasonic pulses from a piezoelectric transducer at an incident angle normal to the wall of a pipeline, after which the pulses are measured at the opposite side of the wall.

In the implementation of conventional ultrasonic gauging techniques, the key to accurately determine the thickness of the wall of a pipeline is by accurately obtaining the time delay Δτ. In [55], the authors investigated the performance of the cross-correlation and model-based estimation (MBE) technique in obtaining the time delay Δτ. An identical cross-correlation approach was also proposed by [59]. The authors of [57] investigated how the same ultrasonic gauging technology can be used to inspect the condition of welded joints of metallic pipelines by using the combination of finite element modelling and longitudinal critically refracted waves, an approach that is commonly used in ultrasonic guided wave inspection.

**Figure 10:**
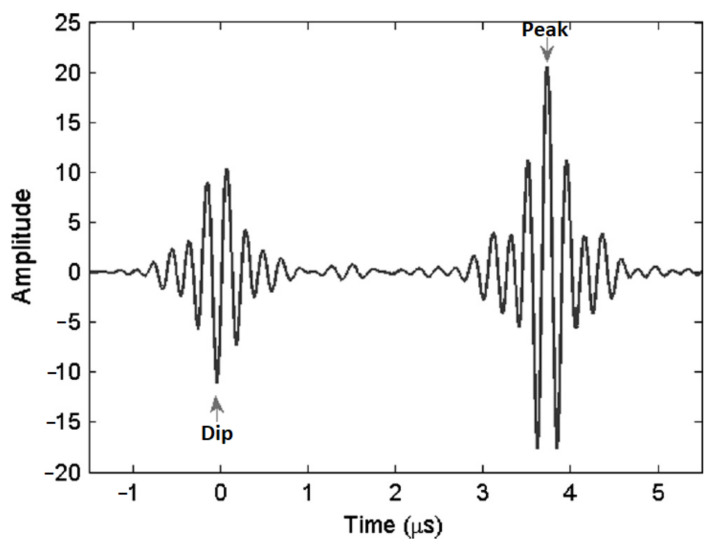
The dip and peak of pulses attributed to the front pipe wall and back pipe wall (designated by the arrows) which are 180${}^{\circ }$ apart in phase. Reprinted from Ultrasonics, Volume 53, Honarvar, F.; Salehi, F.; Sasfavi, V.; Mokhtari, A.; Sinclair, A.N. Ultrasonic Monitoring of Erosion/Corrosion Thinning Rates in Industrial Piping Systems, Page 1251–1258, Year 2013 with permission from Elsevier [55].

The cross-correlation technique proposed in [55] measures the time delay between similar time-series patterns by identifying the dip of the front wall pulse and the peak of the back wall pulse. The reason why the dip-peak pair is used in the determination of the time delay is that the front wall pulse and the back wall pulse are 180${}^{\circ }$ apart in phase. Figure 10 shows the dip and peak (designated by the arrows) of the front wall and back wall pulse, which are 180${}^{\circ }$ apart in phase. In order to improve the time resolution of the pulse measurements, a polynomial fitting is used to interpolate the dip and the peak of the pulses. The time position of the dip and peak is determined by setting the derivative of the polynomial curve to zero.

**Figure 11:**
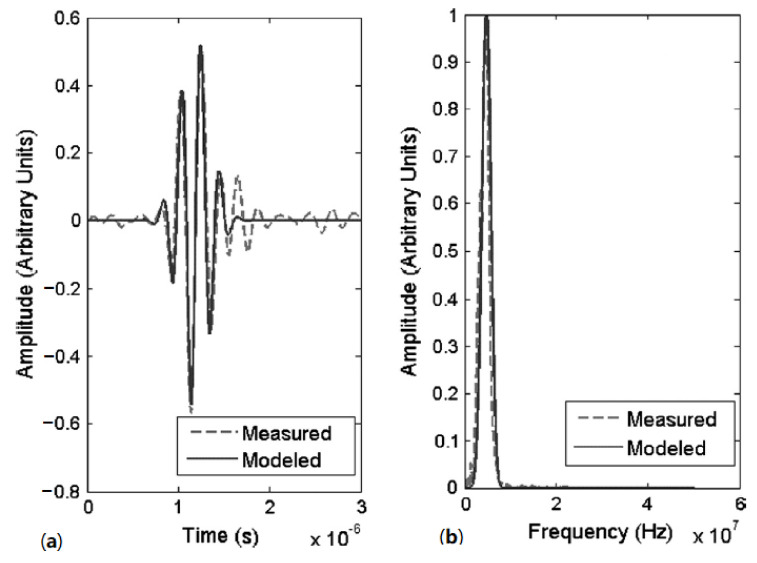
(**a**) The typical measured front wall echo and its corresponding Gaussian pulse. (**b**) The frequency spectra of the measured and modelled pulse. Reprinted from Ultrasonics, Volume 53, Honarvar, F.; Salehi, F.; Sasfavi, V.; Mokhtari, A.; Sinclair, A.N. Ultrasonic Monitoring of Erosion/Corrosion Thinning Rates in Industrial Piping Systems, Page 1251–1258, Year 2013 with permission from Elsevier [55].

The MBE technique uses a Gaussian profile to model the ultrasonic pulses. Figure 11a shows a typical measured front wall echo and its corresponding Gaussian pulse. Despite the removal of noise from the measured echo by the Gaussian filter, the frequency spectra of both the measured and modelled pulses are almost identical as shown in Figure 11b. By filtering out the noise in the pulses, the time delay between the front wall and back wall pulses can be accurately determined.

Both the cross-correlation and MBE technique were found to be able to estimate the thinning rates as low as 10 µm/year within 15 days with low uncertainties of ±1.5 µm/year with a 95% confidence interval. Despite being marginally more accurate than the cross-correlation technique, the MBE technique is more computationally complex. Therefore, the cross-correlation technique is favoured in industrial applications.

In order to achieve sub-micron accuracy in ultrasonic gauging, [56] mentioned three difficulties that must be overcome. The difficulties are the following:

- The stability of the mechanical assembly of the ultrasonic gauge over a long period for consistent accuracy of the measurements. This can be achieved if the ultrasonic gauges are assembled professionally by the manufacturers.
- The effectiveness of the interpolation or filtering signal processing scheme to achieve the desired accuracy. This is addressed in [55]
- The compensation of the temperature effects. A temperature compensation strategy was proposed in [56].

The temperature compensation strategy in [56] first calculated the function $v^{*}\left(T\right)$, from the initial thickness of the pipeline $th_{ref}$, the experimental values of $t_{0f}\left(T,0\right)$, which is the time-of-flight of the ultrasonic wave at temperature *T*, and change in the thickness of the pipeline δ=0, with the assumption that the thickness remains constant during the period of measurement. $v^{*}\left(T\right)$, which is the longitudinal velocity of the ultrasonic wave at temperature *T* is expressed as follows:

(6) $$v^{*}\left(T\right)=\frac{2th_{ref}}{t_{of}\left(T,0\right)}$$

Then, the measured value of the time of flight at temperature *T* and geometrical changes δ, due to the thermal expansion of the material of the pipeline, can be translated to the temperature-compensated thickness of the pipeline $th(T_{ref},\delta$) by using the following:

(7) $$th\left(T_{ref}m\delta \right)=\frac{v^{*}\left(T\right)t_{of}\left(T,\delta \right)}{2}$$

### 2.4. Ground Penetrating RaDAR (GPR) (Leakage)

**Figure 12:**
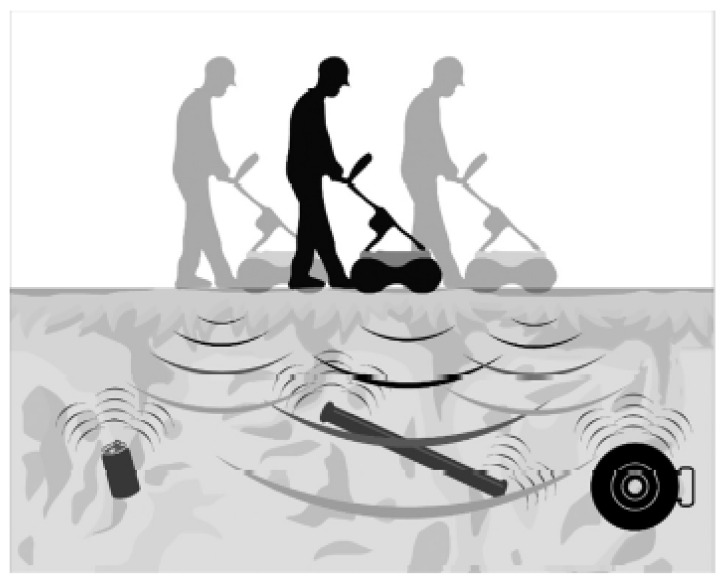
GPR survey conducted using a human-operated device. Reprinted under Creative Commons CC BY-NC license [64].

The ground-penetrating RaDAR (GPR) is a technology that can be equipped on a vehicle or a human-operated tool as shown in Figure 12 to perform underground inspection of buried objects. The GPR detects electromagnetic contrasts in the soil by transmitting and receiving specified high-frequency electromagnetic waves. The electromagnetic waves are distorted in terms of amplitude, phase-shift and frequency depending upon the types of reflecting materials in the soil [64]. In [60], GPR was used to assess the condition of peatlands, with thickness ranging from 1.5 m to 2.5 m, for the planning of pipeline placement.

**Figure 13:**
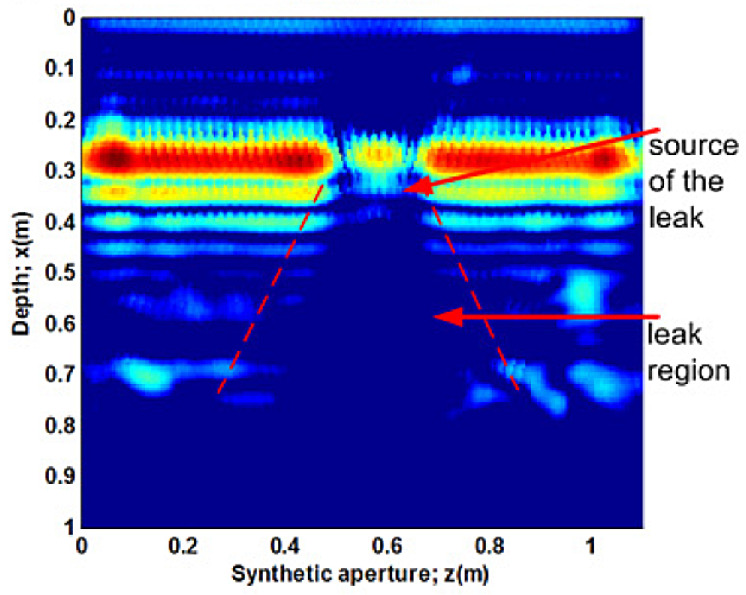
The GPR image of a leaking water pipeline. Reprinted from NDT & E International, Volume 47, Demirci, S.; Yigit, E.; Eskidemir, I.H.; Ozdemir, C. Ground Penetrating Radar Imaging of Water Leaks from Buried Pipes Based on Back-projection Method, Page 35–42, Year 2012 with permission from Elsevier [2].

In [2], the distortion of the electromagnetic wave was measured in the form of a two-dimensional brightness scan (B-Scan). A series of overground B-Scans of the pipelines were put together and reconstructed using a back-projection algorithm to produce the image shown in Figure 13. A similar back projection approach was employed in [61] for the 3D image reconstruction of underground pipelines. The back-propagation algorithm proposed in [2] takes into account the compensation for the wave-front curvature effects, the effect of the finite bandwidth of the antenna and the refraction of the electromagnetic wave at the air–ground boundary. All these considerations improve the contrasts of the image pixels, which allows a clearer visualisation of the water loss events. The conventional back-projection method used in [2,61] employs Maxwell’s equations for the modelling of the electromagnetic wave propagation of the GPR. The inhomogeneous medium of propagation, such as varying soil densities and the abrupt change in the density at the soil–pipe interface, may result in the presence of uncertainties in the mathematical model. This concern is addressed in [62], using the Bayesian approximation error approach.

The authors of [63] provided a theoretical solution to understand the behaviour of GPR in real-world complex geological structures, using the finite-difference time-domain method (FDFT), which allows the depth of an underground pipeline to be computed immediately after the time-of-flight of the incident wave. The FTFD mathematical model was simplified by constraining the detection target to a two-dimensional problem. The simulation results show that the deviations between the computed depth and the actual depth of the underground pipeline are 4.87% and 2.03% when the actual depths are 10 cm and 30 cm, respectively. The error in the computation is attributed to the simplification of the FTFD model but is within the acceptable range for underground pipeline exploration [63].

The authors of Li et al [65] further improved the performance of GPR in underground pipeline mapping by fusing the GPR scans in the form of hyperbolic responses with camera images. The inspection was implemented using a robot consisting of a GPR sensing unit, a camera and an on-board computer for data fusion. Simultaneous locating and mapping of the head-on surrounding of the robot as the robot moves provide visual cues to classify the GPR scans into groups belonging to different pipelines; thereafter, a 3D image of the underground pipelines is reconstructed. This method is effective in differentiating multiple overlapping pipelines in the same region of interest. Authors of [65], in their experimental setup, were able to produce a reconstructed 3D image of the underground pipeline network with a localisation accuracy of 4.47 cm and orientation errors of 1.73${}^{\circ }$ and 0.73${}^{\circ }$ for the two pipe orientation angles present in the network.

The computational challenge in the detection of leakages using the GPR technology exists in the reconstruction of the final image using a series of scans. The effectiveness of the algorithm used in the reconstruction process affects the quality of the image in terms of the clarity, resolution and contrast. While GPR provides near real-time visualisation of the condition of a pipeline, unless a computerised approach such as that in [65] is used, human intervention is required since the decision-making process is based on one’s perception of the indicators of water loss events in the GPR image.

### 2.5. Impact Echo (IE) (Leakage, Crack)

**Figure 14:**
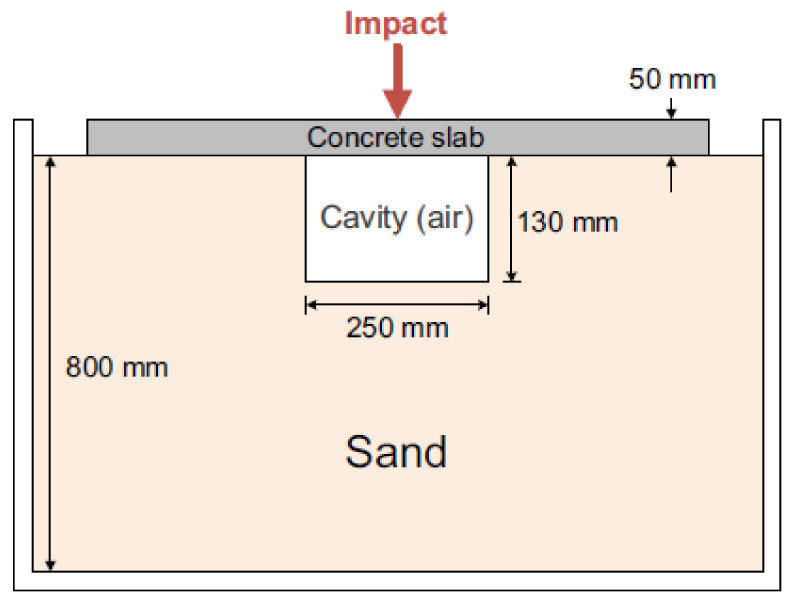
The schematic drawing of the impact echo test. Reprinted from Tunnelling and Underground Space Technology, Volume 65, Kang, J.M.; Song, S.; Park, D.; Choi, C. Detection of Cavities around Concrete Sewage Pipelines Using Impact-echo Method, Page 1–11, Year 2017 with permission from Elsevier [19].

Impact echo is a form of reflectometry that detects the presence of underground cavities under a concrete slab by employing the law of reflection. In the presence of leakages in underground pipelines, cavities form around the leakages due to the run-off of fluid carried by the pipelines. In [19], by dropping a steel ball onto the concrete slab, a stress wave was generated and transmitted as shown in Figure 14. The wave propagated towards the sand beneath the concrete slab. By measuring the sustained duration, a parameter denoting the width of the curve on a normalised amplitude of the resonant frequency component versus time plot, of the reflected wave, the presence of cavities was able to be determined. In a normal scenario, the wave is reflected by the surface of the sand and attenuated almost immediately by the concrete slab, resulting in a short sustained duration.

However, if a cavity is present, due to the air gap between the concrete slab and the bottom of the cavity, the reflected wave experiences less attenuation, resulting in a longer sustained duration. The sustained duration is precisely defined in [19] as the period in which the amplitude of the normalised resonant frequency component is above 0.8. The resonant frequency is determined using a short-time Fourier transform of the time-series reflected wave.

In [67], using the same principle as [19], crack detection was achieved by correlating the depths of the cracks present in an underground pipeline with the amplitudes of the resonant frequencies. Refs [19,67] suggested to first correlate the resonant frequency measured on the spectrogram with the distance of the target from the surface of the ground, using the following:

(8) $$f_{r}=\frac{0.96c}{2T}$$

where $f_{r}$ is the resonant frequency of the reflected wave, *c* is the speed of the wave in the propagation medium, *T* is distance of the target from the surface of the ground and 0.96 is the correction factor that has been incorporated in the American Society for Testing and Materials (ASTM) standards. The validation of the correction factor can be found in [68], where the guided wave theory was used to develop a numerical model on the basis of the Lamb wave. However, the approach proposed in [67], instead of measuring the sustained durations of the resonant frequency components, correlated each resonant frequency to the presence of one underground boundary, where the lowest resonant frequency with the highest amplitude is attributed to the pipeline itself, and other resonant frequencies are attributed to the cracks present on the wall of the pipeline. By cross-correlating the *T* values for each resonant frequencies calculated using Equation (8), the depth of the cracks were able to be determined.

The impact echo is computationally simple since the presence of cavities and cracks can be determined by analysing the resonant frequencies on the spectogram of the reflected impact wave. The short time Fourier transform required to determine the resonant frequency [19] is also a commonly used signal processing technique, which is effective in determining the frequency components of signals with time-varying frequencies. However, the approaches in [19,67] are not suitable for the detection of micro-cracks since their detection sensitivity is higher than 40 mm. A similar approach was proposed in [66] to detect the presence of voids in underground grouted tendon ducts.

The presence of signal cluttering or reflection due to the contact of the impact wave with the edges of the concrete structures during impact echo tests reduces the accuracy of the depth evaluation of underground defects. The authors of [69] introduced a method to compensate for the signal cluttering by first analysing the behaviour of the impact wave at four different reflecting surfaces: air, water, saturated soil and cement paste. Based on the results of the analyses, the compensation, known in [69] as the virtual edge extension technique, was able to resolve the presence of noise in the reflected impact wave.

The impact echo test requires dedicated human intervention, and the area coverage for each inspection setup is limited by the size of the impact hammer. The scalability and generality of the impact echo method are, therefore, limited. The setup parameters of the test also vary greatly with different environments of inspection, such as the thickness of the concrete slabs and the depth of the pipelines underground.

### 2.6. Acoustic Emission (AE)/Vibration Analysis (Blockage, Leakage, Crack, Weld Defect)

Pipelines in operation generate micro translations, turbulence and noise that can be analysed for the detection of anomalies and defects. In [38], the effects of blockages in a circular pipeline using vibration measurements were investigated. The flow of a fluid through an obstacle causes an increase in the velocity of the fluid flowing over the obstacle, resulting in a decrease in the pressure around the obstacle, following Bernoulli’s principle. The phenomenon occurs in the form of a fluctuating disturbance that can be measured in the form of vibrations. The vibration parameters were measured using accelerometers that were mounted onto the external walls of pipelines. The mounting is more challenging for pipelines that are buried underground unless inspection chambers are present for ease of access. The scalability of the methods based on the analyses of acoustic emission and vibrations is limited by the capacity of a pipeline network. Pipeline systems with a larger number of pipes require a larger number of sensor nodes to be installed.

**Figure 15:**
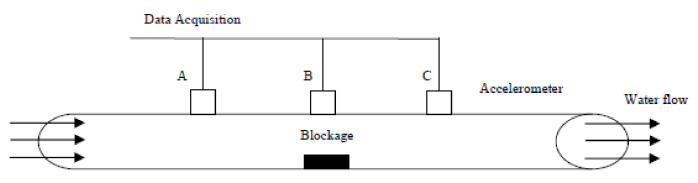
The experimental setup with accelerometers mounted on the external surface of the pipeline for the measurement of vibration signals. Reprinted under Creative Commons CC BY license [38].

**Figure 16:**
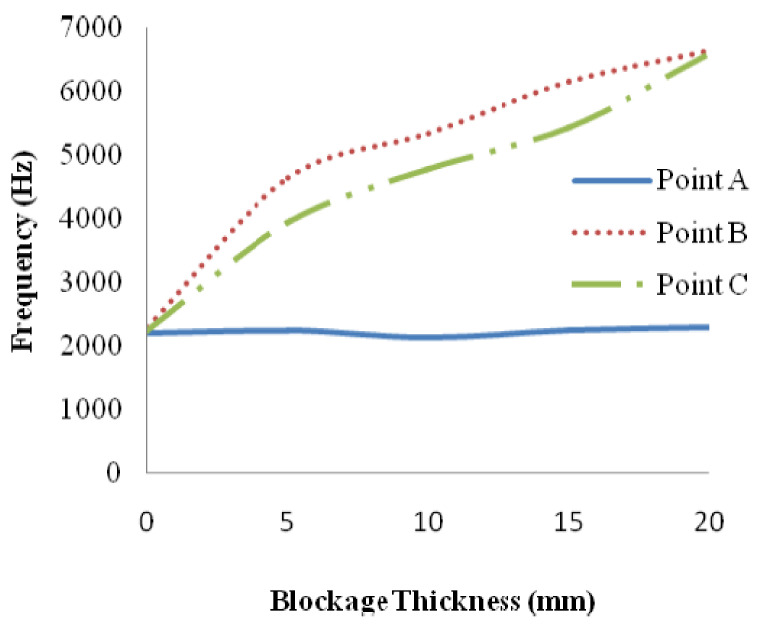
Frequency response at different sensor locations. Reprinted under Creative Commons CC BY license [38].

An analysis was then made to establish the relationship between the blockage levels and the characteristics of the vibration signals observed. The experimental setup is as shown in Figure 15. Figure 16 shows the plot of frequencies of the vibration signals for different accelerometer locations at Points A, B and C. Based on Figure 5, it can be observed that the frequencies of the vibration signals at Point B are the highest, due to the drastic fluctuation in pressure and the highest fluid velocity, compared to that at Point A and Point C [38]. The method proposed in [38] works well for the localisation of blockages. However, if a more refined and accurate localisation is required, the number of accelerometers required needs to be increased since this method determines the position of a blockage based on the gradients of the readings of neighbouring accelerometers.

Variations in vibration signals caused by fouling and clogging pipelines were also studied in [11], using finite element models of fluid-conveying pipelines, developed using ANSYS. In [71], a multi-feature fusion technique based on features of wavelet energy entropy, approximate entropy and fractal box dimension, extracted from acoustic signals collected from the pipelines, was proposed. The classification of the feature sets was achieved using a SVM classifier that was optimised using the particle swarm optimisation (PSO) algorithm. Unlike the method in [38], the approach in [11] detects the presence of blockages in pipelines by distinguishing abnormal vibration signals from the normal ones, using machine learning.

The method in [11], despite being more complex computationally due to the need to collect a large data sample for training, is more cost-effective for large-scale deployment since it eliminates the need for using a large number of sensors. Both of the methods proposed in [11,38], respectively, have different emphases. The study of [38] focused on the accurate localisation and estimation of the sizes of blockages while that of [11] gave weight to fast qualitative identification of blockages in a pipeline network by eliminating real-time signal processing.

The magnitudes of the variations in the vibration signals in a pipeline depend on the severity of both the structural failures and obstruction to load flow. The magnitudes of the variations in the signals need to be sufficiently large for effective signal analyses and feature extraction. Thus, if the dimensions of a blockage are not significant, compared to the length and cross-sectional area of a pipeline, the employment of the vibration analysis results in false negative detections. It is also important to note that the attenuation of the vibration signals are affected by the building materials of the pipelines. Therefore, the strategic placement of sensors and the selection of accelerometers with sufficient sensitivity are necessary.

Structural failures in pipelines, such as leakages or cracks, generate acoustic emissions that propagate in the form of hydrodynamic transients in the fluid. These transients are characterised by pressure and velocity oscillations. In [9], experimental tests were conducted to evaluate the performance of acoustic and mass balance methods. Leakages in a pipeline were simulated by the opening of solenoid valves. The acoustic approach, which accounts for acoustic attenuation, is expressed as follows:

(9) $$\Delta P\left(x\right)=\Delta P_{0}\exp\left(-\alpha x\right)$$

where ΔP(x)= the amplitude of the pressure pulse at distance *x* from the leakage location and α= the attenuation coefficient of the fluid medium in the pipeline. In order to calculate the amplitude of the pressure pulse at the point of leakage ($\Delta P_{0}$), the distance of the leakage from the inlet of the pipeline (*ℓ*) is calculated based on the difference in the arrival times of the pressure transients at the inlet and outlet of the pipeline. In Equation (9), *x* is substituted with the distance of the leakage from the inlet of the pipeline (*ℓ*), and ΔP(x) is expressed as the average of the amplitudes of pressure measured at the inlet ($\Delta P_{in}$) and the outlet of a pipeline ($\Delta P_{out}$), resulting in the following:

(10) $$\Delta P_{0}=\frac{\Delta P_{in}+\Delta P_{out}}{2\exp\left(\alpha \ell \right)}$$

By resolving $\Delta P_{0}$, Equation (9) can now be used to locate the distance *x* of a leakage more efficiently by measuring ΔP(x) at only one point in a defined length of pipeline, instead of using pressure values at two points in a pipeline to resolve the location of one leakage.

The leak flow rate ($m_{leak}$) is calculated using Equation (11) in terms of the diameter of the pipeline (*D*), amplitude of pressure measured at the inlet of the pipeline ($\Delta P_{in}$), amplitude of pressure measured at the outlet of the pipeline ($\Delta P_{out}$), attenuation coefficient of the fluid medium (α) and distance of the leakage from the inlet of the pipeline (*ℓ*).

(11) $$m_{leak}=\frac{\pi D^{2}}{8a}\cdot \frac{\Delta P_{in}+\Delta P_{out}}{\exp\left(\alpha \ell \right)}$$

It was found that, with relatively simple computations based on Equations (9)–(11), the acoustic approach provides an accurate indication of the location of leakages. However, despite the approximately zero error of the acoustic approach in the localisation of leakages, the systematic error for the estimation of leak flow rate using the same approach is higher at 3.21 L per minute. In order to resolve the high systematic error in the estimation of leak flow rate, a mass balance approach was employed. The principle of the conservation of mass for the approach is expressed fundamentally in Equation (12), where ΔT= the integration interval, $m_{leak}=$ the leak mass flow rate, $m_{in}=$ the mass flow rate at the input of the pipeline and $m_{out}=$ the mass flow rate at the output of the pipeline. The mass approach was able to estimate the leak flow rate with an approximately zero error.

(12) $$m_{leak}=\frac{1}{\Delta T}\int_{\Delta T}^{}\left(m_{in}-m_{out}\right)dt$$

The acoustic approach is able to resolve the location of a leakage based on the amplitude of the pressure pulse measured by one pressure sensor. However, the placement of sensors should take into account limitations, such as the sensitivity of the sensors and the degree of attenuation during the propagation of the oscillations. Unlike the acoustic approach, the mass-balance approach requires continuous monitoring of the mass flow rate, using two pressure sensors in a defined length of the pipeline. While observing similar limitations as those of the acoustic approach, due to the use of the readings from two pressure sensors in resolving the leak mass flow rate, the mass balance method is less efficient, computationally. In practical scenarios, because of the mass variation due to the origins of errors mentioned earlier, the integration effort as shown in Equation (12) employed in the mass-balance method causes the accumulation of errors originating from the thermal and elastic expansion of the pipeline, as well as the compressibility of the fluid.

**Figure 17:**
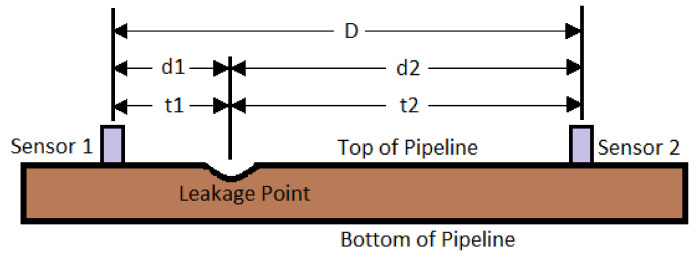
The experimental setup for the identification of the location of the leakage based on the difference in arrival times of acoustic signals, over distances d1 and d2. Modified from [72].

In [72], the acoustic signals emitted by the leakage were captured using two sensors that were installed separately at two ends of the pipeline as shown in Figure 17. The location of the leakage was identified, using a cross-correlation technique to calculate the difference in the arrival times of the acoustic signals at both of the sensors. Unlike the methods in [9], wavelet transform was employed in [72] for noise reduction of the signals captured by the sensors. The effort of noise reduction involves the removal of high-frequency oscillations from the desired signals to promote more effective signal analyses for better localisation of the water loss events.

Based on Figure 17, distances d1 and d2 could be calculated by the following:

(13) d1=(D+c·Δt)/2

(14) d2=(D−c·Δt)/2

where c= the propagation speed of the acoustic signals, D= the distance between the sensors, and Δt= the time delay of the arrival of acoustic signals at the sensors [72]. Δt can be precisely estimated as the value of τ corresponding to the peak of weighted cross-relation function $\hat{R_{xy}}\left(\tau \right)$, which is expressed in [72] as follows:

(15) $$\hat{R_{xy}}\left(\tau \right)\;=W_{ML}\left(\tau \right)*R_{xy}\left(\tau \right)$$

(16) $$R_{xy}\left(\tau \right)=\alpha R_{xx}\left(\tau -\Delta t\right)$$

where $W_{ML}\left(\tau \right)$ is a continuous time optimal maximum likelihood weighting function, used to sharpen the peak in cross-relation function $R_{xy}\left(\tau \right)$.

Like the approach in [9], the method proposed in [72] measures propagating acoustic emission from stationary points. This approach of measurement allows the minimisation of the number of sensors installed through a strategic placement of sensors based on various determinable variables, such as the operating environment, the attenuation coefficient of the fluid transported in the pipeline network and the sensitivity of the sensors. Methods based on the measurement of localised hydraulic parameters, on the other hand, require a high-density sensor distribution in order to accurately localise water loss events. If the sensors are spaced too far apart, there is a high probability that the sensor network will fail to detect small leakages or cracks that are present in the physical gap between the two adjacent sensors.

Despite the similarity mentioned, the method in [72], unlike the approach in [9], requires continuous monitoring of sensor readings in pairs. Apart from that, the method in [72] uses accelerometers instead of pressure sensors. The installation of the accelerometers is non-invasive since they are mounted on the external walls of pipelines.

The detect and classification of acoustic third-party damage (TPD) signals for pipelines using the least square support vector machine (LS-SVM) was proposed in [30]. Using wavelet packet decomposition, the TPD signal was decomposed using wavelet packet decomposition, after which the wavelet packet energy was selected as a feature for the LS-SVM. This method successfully classified four TPD signals: normal, drilling, hammering and excavating conditions, with a success rate of more than 85%. By classifying the different TPD signals, preventive maintenance can be conducted by eliminating their respective sources to prevent damage to the pipelines. The work in [30] can be adapted to complement the approaches in [9,72] by classifying the types of defects.

In [10], the authors proposed an approach using a local projection procedure instead of the wavelet transform methods to remove noise from acoustic signals. The local projection procedure was able to retain the high-frequency contents in the acoustic signals. These high-frequency characteristics are generated by high-speed jet flow from small pipeline leakages. There is a tendency for the wavelet transform to eliminate high-frequency components that are contributed by small water loss events.

Despite the large variation in the computational and deployment costs incurred by the methods in this category, due to the abundance of the underlying approaches used as well as the types of sensors employed, the overall cost remains lower than that of the reflectometry methods for large-scale deployment since the sensor network is mostly in the form of mainstream embedded systems, is highly scalable and requires minimal human intervention. Unlike the reflectometry methods, the measurement of acoustic emission and vibration can take place while the pipeline system is operating, allowing the monitoring and detection of defects in real time. At the same time, acoustic sensors are installed by latching them to the external wall of a pipeline. The access to the internal environment of a pipeline network, like that required by the flow and pressure sensors in the sensing of hydraulic parameters, is not required.

**Figure 18:**
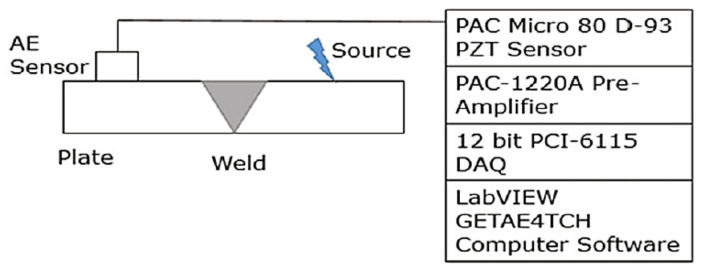
The arrangement of the AE sensor and acoustic source. Reprinted from Journal of Constructional Steel Research, Volume 134, Droubi, M.G.; Faisal, N.H.; Orr, F.; Steel, J.A.; El-Shaib, M. Acoustic Emission Method for Defect Detection and Identification in Carbon Steel Welded Joints, Page 28–37, Year 2017 with permission from Elsevier [1].

In [1], the acoustic emission (AE) method was used to detect weld defects. Unlike the acoustic emission methods employed in the detection of blockages, for cracks and leakages that rely on the analysis of the acoustic waves emitted by the defects naturally when the pipelines are in operation, the detection of weld defects is only possible by transmitting acoustic waves through the girth welds and, subsequently, detecting the acoustic waves that are emitted by the welds. The emissions are caused by the interactions between the elastic waves and the surfaces of the welds. The pencil lead break (test) is used as the source to transmit acoustic waves through the welds. The characteristics of the AE signals depend on the geometry and the elastic properties of the girth weld. These characteristics include the energy levels, peak amplitudes and root mean square (RMS) amplitudes of the signals. By sampling the signals with a priori knowledge about the conditions of all the girth welds in a pipeline network and subsequently classifying the signals based on the aforementioned characteristics, the presence of weld defects can be identified based on the taxonomy of the signal samples. Figure 18 shows the placement of the acoustic source and the AE sensor with respect to the girth weld on a pipeline.

Unlike the ultrasonic phased arrays, the AE method does not require precise hardware design since no multi-layer inspection that requires high directivity is involved. The lack of directivity of the acoustic waves generated by the pencil lead break test only allows the qualitative monitoring of the condition of a girth weld. This is due to the lower resolution of the AE signals, unlike that of the reflected ultrasonic echoes in ultrasonic reflectometry. Therefore, the reconstruction of the two-dimensional image of the girth weld is not possible. Since the AE method involves only the use of signal processing techniques to extract features from the AE signals, the computational cost incurred is cheaper, compared to that of the ultrasonic reflectometry that requires both signal and image processing techniques. The AE method is a form of localised inspection since each girth weld requires one acoustic source–sensor pair. Therefore, the number of acoustic source–sensor pairs in a pipeline network is proportional to the number of welding points that are present. If the pencil lead break test is used as the acoustic source, challenges in terms of scalability will arise in real-life deployment since continuous human intervention is required. The use of an automated acoustic transmitter is, therefore, necessary to make the AE method practical for the inspection of industrial pipelines.

### 2.7. Resonance Shift Analysis (Blockage, Leakage, Crack)

**Figure 19:**
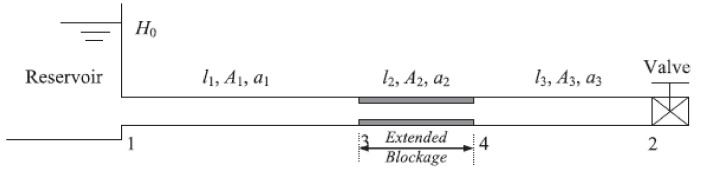
Pipeline with single extended blockage. Reprinted from Journal of Fluids and Structures, Volume 46, Duan, H.F.; Lee, P.J.; Ghidaoui, M.S.; Tuck, J. Transient Wave-blockage Interaction and Extended Blockage Detection in Elastic Water Pipelines, Page 2–16, Year 2014 with permission from Elsevier [74].

The resonant frequency of a pipeline is the natural frequency of the vibration in the wall of the pipeline in which matter flows. Pipelines made of different materials and different sizes have different resonant frequencies. The analysis of system resonant frequency is especially effective for the detection of extended blockages. An extended blockage causes a reduction in the cross-sectional area of a pipeline, which disrupts the flow of fluid, leading to changes in the hydraulic parameters and, hence, a shift in the resonant frequency. A method to detect the presence of extended blockages, employing the inspection of the shift in the resonant frequency of a pipeline system, was numerically and experimentally validated in [73,74]. Unlike discrete blockages, extended blockages, as shown in Figure 19, induce changes to system resonant frequencies. The shift in resonant frequency normalised by the fundamental frequency of a uniform blockage free pipeline ($\Delta \omega_{rf}$) is expressed in [74] as follows:

(17) $$\begin{array}{c} \Delta \omega_{rf}=\frac{\epsilon_{A}}{\lambda_{0}\left(2-\epsilon_{A}\right)}[\sin\left(2\lambda_{1}\omega_{rf0}\right)-\sin\left(2\lambda_{3}\omega_{rf0}\right)\\ +\frac{\epsilon_{A}\sin\left(2\epsilon_{L}\lambda_{0}\omega_{rf0}\right)}{\left(2-\epsilon_{A}\right)}] \end{array}$$

where $\epsilon_{A}=$ change of pipe cross-sectional area, $\epsilon_{L}=$ the longitudinal range of the blockage, $\lambda_{0}=$ the wave propagation coefficient of the uniform blockage-free pipeline, $\lambda_{1}=$ the wave propagation coefficient of the region before the blockage, $\lambda_{3}=$ the wave propagation of the region after the blockage and $\omega_{rf0}=$ the resonant frequency of the uniform blockage-free pipeline.

By measuring the change in the resonant frequency and the wave propagation coefficients of the fluid corresponding to $\lambda_{1}$ and $\lambda_{3}$, Equation (17) can be solved numerically to quantify the cross-sectional area and the longitudinal length of a blockage. Due to the relatively large number of measured values required before the numerical computation can be carried out, the accuracy of the quantification is compromised by the reliability of the sensor readings. On top of that, the accuracy also declines with the increase in the percentage of the cross-sectional area of the pipeline covered by the blockage due to the increase in the degree of uncertainty of the mathematical model. Moreover, invasive access is required since piezoelectric sensors for the measurement of pressure signals have to be installed inside the pipelines. Unless the sensors are installed in strategic locations, the localisation of blockages based on the analysis of the shift in the resonant frequency could be challenging.

From another perspective, the effectiveness of pipeline inspection based on the analysis of the resonant frequency is not constrained by the dimensions, the shape and the building materials of pipelines. However, the detection of discrete or unextended blockages is not favourable for inspection based on the resonant frequency since these blockages may not cause an appreciable shift in the resonant frequency of a pipeline network. This drawback is addressed in [77], where an energy analysis involving the study of the energy transmission coefficient patterns to categorise various forms of non-uniform blockages was proposed. On top of that, different types and dimensions of pipelines have different natural frequencies and acoustic behaviours, rendering the generality and scalability of the methods in this category computationally challenging.

In the presence of structural discontinuities, such as cracks, variations in the natural or the resonant frequency of the vibration in the wall of a pipeline occur. In [75], a split ring resonator (SRR) was designed for the detection of leakages, corrosion and cracks in pipelines, particularly to detect ruptures in pipeline coatings made of FR4 epoxy. The SSR, tuned to a resonant frequency of 6.1 GHz, was simulated, using the high-frequency structure simulator (HFSS) tool to detect ruptures in pipeline coatings. In the presence of discontinuities on the coating in the form of air gaps, changes in the resonant frequency and quality factor were observed. As the size of the gap increases, which is an indication of the reduction in the structural integrity of the pipeline due to coating failure, the quality factor (sharpness of the peak of the central frequency) of the resonance decreases, while the bandwidth (range between the lower and upper cut-off values) of the resonance increases.

A similar approach of analysis can be used to detect the presence of cracks, ruptures or other structural changes in pipelines. However, the variations may be indistinguishable when the defects are small and positioned at discrete locations outside the effective range of the resonator. In [75], the analysis of the resonant frequency generally involves straightforward computing strategies since any changes in the condition of the pipeline are inferred from the shifts in the quality factors and bandwidths of the resonant profiles plotted as S21 gain versus frequency graphs. A drastic shift is observed between the resonant profiles produced by [75] for the 0.1 mm and 0.5 mm air gap. The shift is an indication of a coating failure, which requires immediate attention. A shift in the resonant profiles is also observed when leakages or cracks are present. The method discussed in [75] is highly scalable since it employs a resonator, which can be easily tuned to the resonant frequency according to the specifications of the pipeline that is being tested, unlike those in [73,74] that relied on the measurement of the resonance in real time from the pressure transients. It is also important to note that most conventional resonance shift inspection methods are unable to provide a quantitative evaluation, such as the locations and dimensions of the flaws in the pipeline, since the existing evaluation approaches only target the effective qualitative categorisation of defects.

In [76], an inverse algorithm using maximum correlation function was proposed to quantify the locations and sizes of defects on the test specimen. The maximum correlation approach helps to address the mathematical problem with the presence of infinite combinations of location, size and type parameters for a certain resonance shift measured. This approach assumes that the frequency spectrum shift induced by multiple defects is equal to the sum of the shifts induced by each of the defects independently. It was also assumed in the approach that the frequency shift induced by a defect is different from the shift induced by a defect at another location. The investigation of the influence of different defect locations and different defect sizes on the frequency shift allows all possible unit locations of defects on a test specimen to be indexed, according to their unique frequency shifts. The size of a defect on the specimen is then represented by *N* units of defects, where the influence of the frequency shift can be made known through linear superposition. The aforementioned assumptions and investigation allow the formulation of an inverse algorithm to quantify the characteristic of a defect by maximum correlation of the frequency spectra. Another method in [78] employs the use of guided microwave inspection to monitor the resonant frequency shift of the biofilm deposit on the internal wall of a pipeline. Biofilm is a common deposit caused by the accumulation of microorganisms on the internal surfaces of fluid-handling pipelines. Excessive thickening or lengthening of the biofilm may result in the formation of a blockage in a pipeline. By monitoring the resonant frequency shift of the biofilm over time, the growing rate of the biofilm can be evaluated quantitatively, using the following equation:

(18) $$V_{biofilm}=\frac{\pi d^{2}l}{2(\epsilon_{r}-1)}\left|\frac{\Delta f}{f}\right|$$

where $V_{biofilm}$ is the volume of the biofilm, *d* is the diameter of the pipe and, $\left|\frac{\Delta f}{f}\right|$ is the normalised resonant frequency shift, and $\epsilon_{r}$ is the change in permittivity caused by the biofilm, or the relative permittivity of the biofilm.

### 2.8. Hydraulic Transient Analysis (Blockage, Leakage)

The hydraulic parameters, in the context of this paper, are focused mainly on the direct use of fluid transients, such as pressure and flow rate, which can be measured using sensors that are inexpensive and readily available. Since the measurement of hydraulic parameters is involved, methods in this category can only be employed for fluid-filled pipelines of any building materials. The presence of blockages in the pipelines causes localised changes to the flow rate and pressure of the fluid. These changes are captured by nearby hydraulic sensors. In [79], the detection of a blockage, using an implicit finite difference modelling, was demonstrated using computer simulations. By using only the measured values of pressure and flow rate at the inlet and the outlet of a pipeline, the size and the location of the blockage could be computed effectively, even in the presence of measurement noise. The reliance on the measured values of pressure and flow rate makes the methods in this category highly transferable among pipelines carrying similar substances.

**Figure 20:**
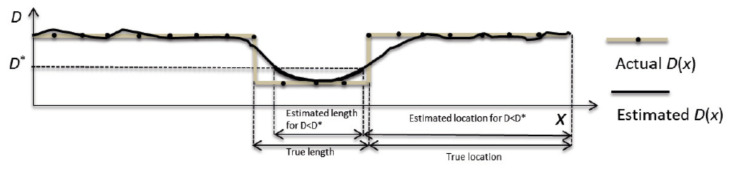
Estimated diameter and actual diameter along the length of the pipeline at point *X*. Reprinted from Procedia Engineering, Volume 70, Massari, C.; Yeh, C.J.; Ferrante, M.; Brunone, B.; Meniconi, S. A Stochastic Tool for Determining the Presence of Partial Blockages in Viscoelastic Pipelines: First Experimental Results, Page 1112–1120, Year 2014 with permission from Elsevier [80].

A stochastic tool, based on the successive linear estimator (SLE) proposed in [80] was able to provide a good estimate of the length and the size of the blockage in a single, short duration transient test, by measuring the pressure at different sections of a pipeline using piezoelectric sensors. SLE, despite the presence of many structural errors in the transient simulation, was still able to provide an approximation of the dimensions of the blockage with low relative errors as shown in Figure 20. This was due to the accountability of SLE for the complex geometries of blockages.

Another method used the blockage-induced damping of fluid transients [81] to detect and locate blockages. The harmonic components of the fluid transients, such as pressure and flow rate, were linearly analysed. The damping of each of the harmonic components depended on the size and the position of the blockage. The damping did not depend on the location of measurement and the characteristics of the transient events. In [86], an inverse transient analysis approach was employed in blockage and leakage detection in pipelines. Prior to the calibration of the operating parameters using the genetic algorithm (GA) to predict failure conditions, such as leakage location, leak quantity, blockage location and blockage coefficient, transient responses due to unsteady friction impact were converted from their respective values in the frequency domain to the corresponding values in the time domain.

The methods proposed in [80,81,86], unlike that in [79] that employed raw sensor readings directly in its computations, required further signal analysis to be performed to perceive the fluid transients. The analysis of fluid transients, such as pressure spikes, provides the possibility of evaluating the dimensions and the locations of blockages more accurately at the expense of a higher computational cost. The precision of the transient measurements depends highly on the sampling frequency and the resolution of the sensors. The computational cost of the methods in this category do not differ too much from methods based on acoustic emission and vibration analysis since the number of sensors required by these methods are proportional to the size of the pipeline plant and the desired sensitivity of detection. The deployability of sensing systems for hydraulic parameters can be measured based on the intensity of effort required for the installation of sensors, which depends on the number of sensors and the ease of access to the internal environments of the pipelines. Methods based on the analysis of the resonant frequency incur a higher computational cost since the value of the resonant frequency cannot be measured directly by a sensor. The value has to be computed using readings of the hydraulic parameters before the shift in the resonant frequency can be calculated as the basis to evaluate the dimensions of the blockages in a pipeline network.

For reliable blockage detection, methods based on the measurement of hydraulic parameters have to overcome the fluctuations of sensor readings caused by events, such as micro-turbulence and sudden surge in water demand. The placement of sensor nodes in the pipeline network also plays an important role in the sensitivity of the sensor network towards the presence of blockages in various locations. While hydraulic curves formed from the interpolation of discrete sensor readings can be denoised using various techniques, such as wavelet transform and Kalman-filtering methods [13,31], uncertainties are present in the physical gaps between adjacent sensor nodes. These uncertainties affect the sensitivity of the system in the detection of small blockages.

Hydraulic parameters, such as pressure, flow rate and velocity, are also sensitive to hydrodynamic changes that are caused by the degradation in the structural integrity of pipelines, which often manifest in the form of cracks or leakages. The availability of a wide array of inexpensive sensors for hydraulic parameters allows the monitoring of these parameters to be carried out continuously in a real-time system. In the context of leakage and crack detection, the computational costs for methods based on the analysis of hydraulic parameters are comparable to those of acoustic emission since both the categories of methods monitor changes in the hydrodynamic characteristics of the fluid flowing in a pipeline. The only notable difference is in the type of sensor used. In the context of the analysis of hydraulic parameters, methods that rely on a gradient analysis instead of transient analysis of the fluid pressure have a relatively lower computational cost.

**Figure 21:**
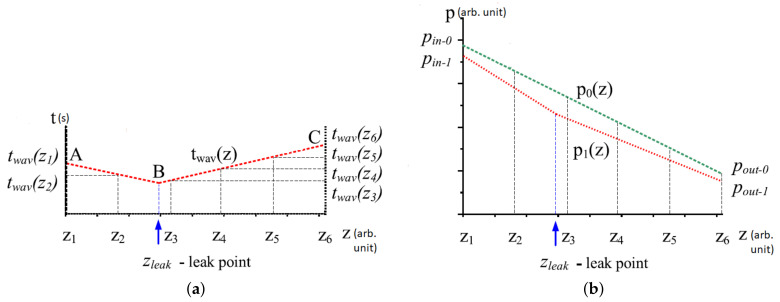
(**a**) Negative pressure wave method analysis. (**b**) Gradient analysis method. Reprinted from Engineering Structures, Volume 113, Ostapkowicz, P. Leak Detection in Liquid transmission Pipelines Using Simplified Pressure Analysis Techniques Employing a Minimum of Standard and Non-Standard Measuring Devices, Page 194–205, Year 2016 with permission from Elsevier [82].

In [82], efficient solutions for the detection of a single leakage based on negative pressure wave and gradient methods were proposed. A negative pressure is generated due to a sudden drop of pressure at the point of leakage. This pressure drop is then propagated upstream and downstream of a pipeline as negative pressure waves, which are detected by pressure sensors that are arranged equally apart along the length of the pipeline at different times ($t_{wav}\left(z_{1}\right)$, $t_{wav}\left(z_{2}\right)$, $t_{wav}\left(z_{3}\right)$, $t_{wav}\left(z_{4}\right)$, …). By plotting time (*t*) against sensor position (*z*) as shown in Figure 21a, the location of the leakage ($z_{leak}$) corresponds to the intersection point of straight lines AB and BC.

Changes in the pressure gradients along the length of a pipeline can be used to locate a single or multiple leakages. In Figure 21b, where $p_{0}\left(z\right)=$ the pressure gradient in a normal pipeline and $p_{1}\left(z\right)=$ the pressure gradient(s) in a pipeline with a single leakage, the location of the leakage ($z_{leak}$) can be found by identifying the intersection point between two straight lines of different pressure gradients. The number of pressure gradients (straight lines) along the length of a pipeline depends on the number of leakages present. A similar gradient method was proposed in [83]. The methods that are based on the gradient analysis of negative pressure waves are computationally straightforward since no transient analysis and pre-processing of data are required. However, there is a need to pay careful attention in determining the distances between successive sensors based on the sensitivity of the sensors and the desired performance of detection in terms of the severity of leakages.

A mathematical approach based on extended sequential probability ratio technique (ESPRT) was proposed in [12] to detect abrupt changes in the pressure of the fluid flowing in a pipeline. Unlike the original ESPRT, which requires the calculation of the statistical mean of the pressure of the fluid in the pipeline prior to the occurrence of a leakage, this innovative method sets the mean to the value of zero, which addresses the challenge to compute the mean pressure due to the rapidly changing characteristics of the fluids in pipelines. This method based on an improved ESPRT is computationally more costly due to the use of the non-linear time-series forecasting method based on back-propagation neural networks to overcome the limitations in the conventional ESPRT method. Despite the higher computational cost, the statistical method in [12] is more effective in the detection of near real-time abrupt pressure changes in time-series pressure data from a single sensor compared to the methods based on gradient analysis, which requires the processing of negative pressure data from multiple sensors.

Pressure signals measured by sensors are often embedded in noise, which reduces the success rate in the detection of small leakages. In [31], the denoising performance between discrete wavelet transform (DWT), lifting wavelet transform (LWT) and undecimated wavelet transform (UWT) was compared. Wavelet transform methods retain the characteristics and information in a signal after denoising. However, LWT and UWT are able to remove noise more effectively, compared to DWT.

A combined Kalman filter–DWT-based negative pressure wave method was proposed in [13]. The leakage location is obtained by extracting the inflection points of the K-filtered pressure signal, using the DWT method. In [84], the independent component analysis (ICA) technique, which assumes that all source signals are independent, was employed to increase the signal-to-noise ratio (SNR) from pressure signals. However, as mentioned earlier, the wavelet transform tends to remove high-frequency components, which may be attributed to the presence of small leakages. The solution to this can be found in [85], which employs a window function to improve the decay rate in the negative pressure signal to detect small pipeline leakages in the presence of complex noise.

### 2.9. Micro-Electro-Mechanical Systems (MEMSs) (Leakage)

A micro-electro-mechanical system is a micro-scaled electronic platform integrated with an actuator or a sensing unit. The sensing unit of a MEMS measures various types of information input, such as mechanical, chemical, thermal, optical and biological changes. The measured information is processed by electronic integrated circuits (ICs), which act as decision makers. The decisions from the ICs are translated into outputs through mechanical actuators. In the context of pipeline monitoring, accelerometers and piezoelectric sensors are two of the few MEMSs that play an important role in detecting changes in the structural integrity of pipelines by measuring information, such as vibration and pressure, respectively. The method proposed by [38], which was mentioned previously, is a good example of how accelerometers are used to collect vibrational data to detect blockages in pipelines.

In [87], piezoelectric sensors, mounted onto the sides of a robot, were used to scan the internal wall of a pipeline for changes in the hydraulic pressure caused by ruptures or water loss events. These sensors output voltages of different amplitudes with respect to the degree of deflection of the piezo film. When a leakage is present, the uniformity of the hydraulic pressure of the flowing liquid in a pipeline is disturbed, resulting in a heterogeneous distribution of pressure. The size of the leakage affects the magnitude of the pressure disturbance, which is reflected proportionally in the degree of deflection of the piezo film. In [91], a piezoelectric hydrophone sensor is constructed on a MEMS platform to measure the acoustic emission caused by the change in the hydrodynamic transients in the presence of leakage in a pipeline. This sensing technology is relevant with the works in the hydraulic transient analysis section. Piezoelectric-based MEMS sensors are, however, highly sensitive to changes in the ambient temperature and, therefore, are not suitable for sensing in challenging environments.

**Figure 22:**
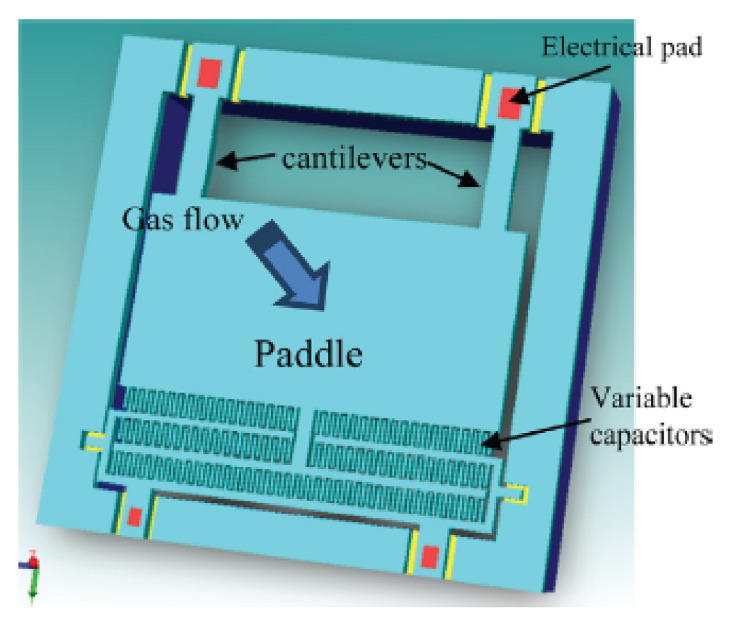
Schematic diagram of MEMS capacitive flow sensor, fabricated using silicon-on-insulator (SOI) wafers. The paddle is perpendicular to the direction of gas flow. Reprinted from Sensors and Actuators, Volume 231, Nguyen, S.D; Paprotny, I.; Wright, P.K.; White, R.M. MEMS Capacitive Flow Sensor for Natural Gas Pipelines, Page 28–34, Year 2015 with permission from Elsevier [94].

Capacitive flow sensors are considered the more reliable counterpart of the piezoelectric sensors, which are more resilient operationally since the sensitivity of this type of sensor is not affected by the changes in the ambient temperature. These sensors are commonly used in measuring the flow rate of the gas transported in a natural gas pipeline. In [92,94], an in-plane MEMS capactive flow sensor as shown in Figure 22 was designed to measure the gaseous flow velocity. The sensor operates on the basis of employing the displacement of a micro-fabricated paddle caused by the dynamic changes in the gas pressure in a pipeline. The variation in the displacement of the paddle varies the capacitance of the variable capacitors. By measuring the capacitance at the electric pads, the velocity of the gas flow in the pipeline can be determined.

In [88], a wireless sensor network (WSN) based on MEMS incorporated the use of accelerometers for measuring vibrations on the external surface of a pipeline to determine the change in water pressure caused by ruptures in the pipeline. While being more affordable, compared to visual optic, thermal and fibre optic sensors due to their general use worldwide in a large array of applications across different disciplines, the data provided by MEMSs are often in the form of electrical readings that can be easily computed, unlike those from fibre optic sensing that require the use of interrogators. However, the scalability of MEMS-based sensing is often constrained by the limited scanning range of these sensors. Most MEMS-based sensors are developed for localised sensing. Therefore, the strategic placement of MEMS-based sensors is crucial to allow a compromise between the deployment cost and the accuracy of localisation.

MEMS sensors also play a very important role in the navigation of inspection gauges or robots in pipelines. In [90], a correction algorithm, involving azimuth and pitch error correction at each pipeline junction, was proposed to enhance the positioning precision of the MEMS Strap-down Inertial Navigation System (SINS) by 54.42%. The authors of [89] implemented a case study on the reliability of the combination of MEMS inertial measurement unit (IMU) and odometer in the tracking of the position of a pipeline inspection gauge (PIG) in a pipeline network. It was demonstrated that the maximum measurement repeatability errors in the use of the combination of measurement units did not exceed 0.25% and 0.1% of the actual pipeline length in the horizontal and vertical directions, respectively. In [93], an overground stationary MEMS piezo-electric based acoustic vector sensor was used to evaluate the position of a PIG running in an underground pipeline network by analysing the acoustic emission generated by the movement of the PIG.

### 2.10. Magnetic Flux Leakage (MFL) (Leakage, Crack, Corrosion)

**Figure 23:**
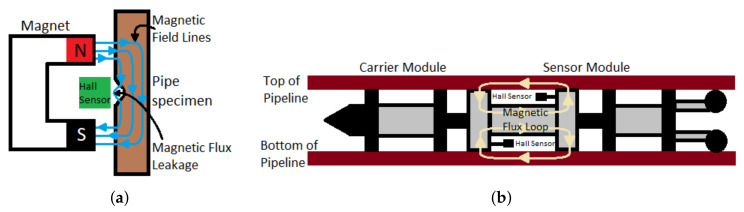
(**a**) Principle of MFL-based inspection for pipelines. (**b**) Layout of a circumferential MFL PIG. Modified from [100].

The distortion of magnetic flux by discontinuities in a solid forms the basis of magnetic flux leakage (MFL)-based detection of cracks and leakages. Unlike the previous methods, MFL-based detection only works for the inspection of pipelines made of ferromagnetic materials, such as steel. The MFL approach requires the induction and saturation of magnetic field lines in the wall of a pipeline. The fields lines can only be induced in metallic materials, which have high magnetic permeability. Figure 23a shows how magnetic flux leakage, caused by a defect on the wall of a pipe, is measured using a Hall effect sensor. A Hall effect sensor outputs a voltage that is proportional to the magnitude of the magnetic field measured.

When no discontinuities are present on the wall, the magnetic field lines are concentrated in the wall of the pipeline. In the presence of discontinuities, the magnetic flux loop breaks, resulting in the leakage of magnetic field lines that are picked up the Hall effect sensor. The computation required for the detection of leakages or cracks by measuring MFL is fundamentally simple since the localisation of surface discontinuities is achieved by distinguishing abnormal readings from normal readings of the magnetic field strength measured by the Hall effect sensor. However, MFL-based inspection is only able to cover a localised area of the internal wall of the pipeline unless it is installed on a device that is capable of moving.

In order to address the limitation of MFL-based inspection in covering the entire length and surface area of the internal walls of pipelines, the design of a circumferential MFL inspection tool was proposed in [4,16,95]. Figure 23b shows the layout of a circumferential MFL pipe inspection gauge (PIG). A pipeline inspection gauge (PIG) travels in the pipeline to perform specific inspection tasks based on the types of sensors equipped. By having a circumferential design, Hall effect sensors can be mounted along the circumference of the PIG. The sensors only have to be mounted onto the PIG, unlike methods based on the analysis of acoustic emission, vibration and hydraulic parameters, which require sensors to be distributed in the pipeline network. This drastically eases the maintenance effort required to ensure the proper functioning of the sensors. However, the transferability of a MFL PIG to various types of pipeline networks is limited by its fixed diameter.

In [16,96], the feasibility of the circumferential design was tested by establishing a finite element model of the tool on ANSYS, an engineering simulation and 3D design software. The results produced in [16] show that the amplitude of the leakage signal increases with the increase in the depth and length of the defect. While further analysis is required to accurately evaluate the dimensions of defects, based on the results in [16], which portray a proportional relationship between the amplitude of the leakage signal and defect size, a basic MFL inspection tool consumes a relatively lower computational cost, compared to methods based on reflectometry, acoustic emission and hydraulic parameters. The combination of a circumferential design and a PIG that is capable of being manoeuvred in a pipeline makes MFL-based inspection a scalable means for pipeline networks of different capacities.

The sampling of the magnetic field strength by the Hall effect sensors while the PIG moves in a pipeline results in the collection of a large number of discrete magnetic field strength readings in the time series and spatial series, respectively. The density of the sampled points and the resolution of the point cloud are dependent on the sampling frequency of the Hall effect sensors and the physical gaps between the sensors. In [97], signal processing methods, such as interpolation smoothing, amendment of singular points, detect-free magnetic flux leakage compensation, and differential magnetic flux leakage processing, were found to be effective against limited sampled points.

In [98], a single hidden layer feedforward network was trained, using an extreme-learning model (ELM) to classify MFL signals in pipeline testing. In ELM, the parameters of the hidden layers of a neural network can be fixed, while the weights of the output neurons are resolved using the least square method. It was found that ELM-based neural networks have a lower training time and a higher accuracy of classification, compared to a general support vector machine (SVM). In [99], a decoupling algorithm was introduced to address the high error rate in determining the depths of defects. The depth of a defect with respect to the peak amplitude of the MFL signal is affected by the length and width of the defect as expressed in the following:

(19) $$D_{depth}=C_{2}\left(l,w\right)B_{peak}^{2}+C_{1}\left(l,w\right)B_{peak}+C_{0}\left(l,w\right)$$

where $B_{peak}=$ peak amplitude of the signal measured by Hall effect sensors, C(l,w)= coefficients of shape factors, l= length and w= width.

The accuracy in the determination of defect depths is severely degraded when significant errors are present in the coefficients of shape factors when these values are obtained through polynomial surface fitting. In order to eliminate the dependency of the defect on the length and width of a defect, the decoupling algorithm in [99], instead of calculating the coefficients in Equation (19), normalises the amplitude values of magnetic flux leakage measured from various defects, with the peak amplitude measured at a maximum depth. By determining the curve of the relationship between the normalised peak amplitude and the depth of the defect, the depths of defects in a pipeline can be determined directly based on the normalised peak amplitude values of magnetic flux leakage measured by the Hall effect sensors.

Other works involving MFL pipeline inspection can be found in [17,101]. In [101], a lightweight MFL inspection tool, using electromagnetic solenoids, was proposed for operation in small pipelines. The permanent magnets of conventional MFL PIG produce a strong adhesive magnetic force that is not suitable for the inspection of smaller pipelines. In [17], a cost-effective MFL inspection method, using commercially available magnetometer, was designed and tested.

**Figure 24:**
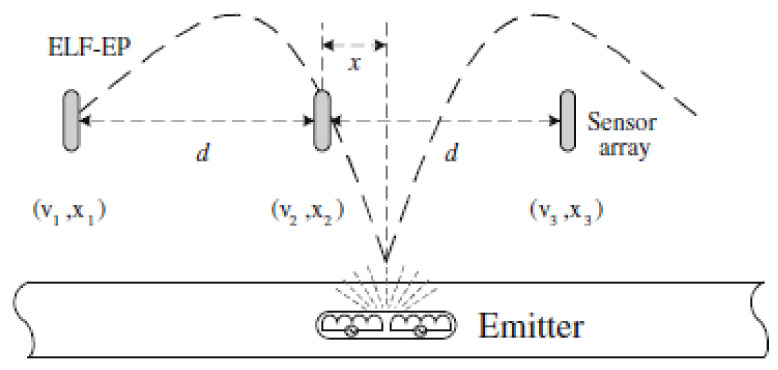
The distribution of ELF sensors outside a pipeline. Reprinted from Mechatronics, Volume 19, Qi, H.; Zhang, X.; Chen, H.;Ye, J. Tracing and Localization System for Pipeline Robot, Page 76–84, Year 2009 with permission from Elsevier [162].

MFL-based inspection is especially suitable for the detection of multiple faults in large pipeline networks. However, the frequency of this means of inspection is constrained temporally by the monitoring schedule of the MFL PIG. Additionally, the saturation of the magnetic flux on the wall of a pipeline, while required to generate the magnetic flux leakage in the presence of discontinuities, diminishes the sensitivity of the inspection system towards smaller defects. Without a reliable in-pipe localisation communication system, the use of MFL-based methods for real-time detection of defects could be challenging to the shielding effect of the metallic walls of pipelines. Unless the PIG is tethered to a data-collection point that is positioned outside the pipeline, the shielding effect prevents the transmission of data from the PIG to the external environment. Another less significant but notable challenge of the employment of PIG for defect detection in pipelines is the localisation of the PIG during its operation. In [162], an in-pipe communication system based on the emission of extremely low frequency (ELF) (23 Hz) was proposed as a means to trace and localise the position of an inspection gauge in the pipeline, using an ELF emitter mounted onto the gauge and arrays of ELF sensors outside the pipeline. The system was experimented in a pipeline network, where the diameter of the pipelines is 315 mm, the thickness of the walls of the pipelines is 12 mm, the pipeline distance is 3 km and the pipelines are 2 to 3 m underground. In the experiment, accuracies of localisations within 1 m were achieved. This localisation system is suitable for the identification of the locations of defects in real time. However, the cost of deployment is increased as the area coverage of the system increases since there is a need to distribute arrays of ELF sensors as shown in Figure 24.

A conventional MFL PIG induces magnetic saturation in the wall of a pipeline to detect the presence of discontinuities, such as leakages and cracks. Due to the large diameter, which is attributed to the circumferential arrangement of permanent magnets, the MFL PIG is not applicable for the inspection of pipelines of smaller diameters. For pipelines that have thick walls, the size and the number of permanent magnets have to be increased to produce sufficiently high magnetic saturation. As the size and the number of the permanent magnets increase, the diameter of the MFL PIG increases proportionally. This scenario limits the deployability of the conventional MFL PIG in the inspection of pipelines that have the combination of small diameters and thick walls. Moreover, the weights of the permanent magnets and the machinery of the driving mechanism as well as the robust structure of the carrier of a MFL PIG may cause mechanical damage as the PIG moves in the pipeline.

**Figure 25:**
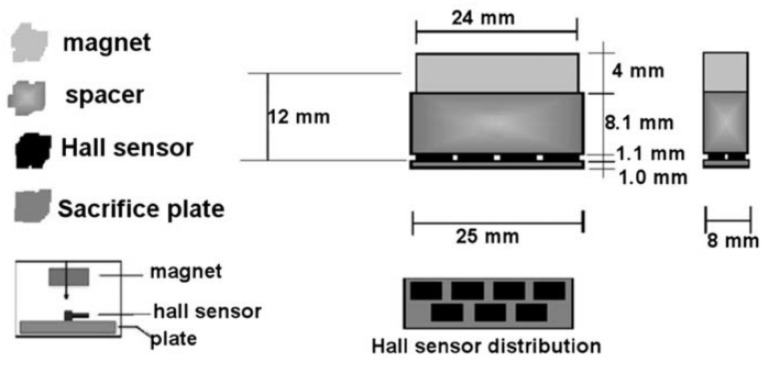
The schematic drawing of the ICS sensor. Reprinted from NDT & E International, Volume 42, Gloria, N.B.S.; Areiza, M.C.L.; Miranda, I.V.J.; Rebello, J.M.A. Development of a Magnetic Sensor for Detection and Sizing of Internal Pipeline Corrosion Defects, Page 669–677, Year 2009 with permission from Elsevier [102].

**Figure 26:**
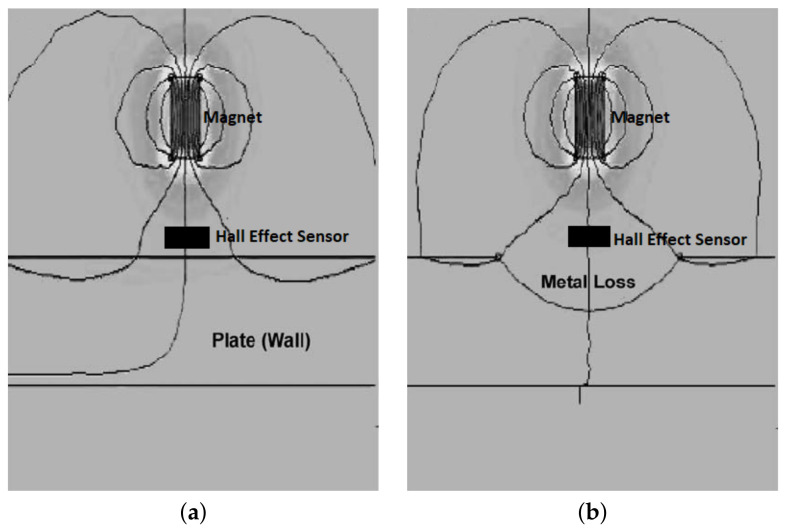
(**a**) Magnetisation of the wall of a pipeline using weak magnetic fields. (**b**) The measurement of the small changes in the magnetic field strength using Hall effect sensors. Reprinted from NDT & E International, Volume 42, Gloria, N.B.S.; Areiza, M.C.L.; Miranda, I.V.J.; Rebello, J.M.A. Development of a Magnetic Sensor for Detection and Sizing of Internal Pipeline Corrosion Defects, Page 669–677, Year 2009 with permission from Elsevier [102].

In conventional MFL PIG, the presence of discontinuities on the surface of the wall of a pipeline is detected based on the significant change in the magnetic field strength at the proximity of the Hall effect sensors caused by the occurrence of the leakage of the magnetic flux. The significant change in the presence of discontinuities is only possible at high magnetic saturation. While high magnetic saturation increases the detectability of severe cracks and ruptures in the wall of a pipeline, it also reduces the sensitivity of the magnetic flux in the detection of smaller defects, such as gradual thinning, grooves and pittings. The limitation of a MFL PIG in terms of the need to achieve high magnetic saturation is addressed in [102], using an internal corrosion sensor (ICS) as shown in Figure 25 to detect metal loss based on field disturbance. The magnet of an ICS, instead of saturating the wall of the pipeline with magnetic flux, as shown in Figure 23a, is oriented to magnetise the wall using weak magnetic fields, as shown in Figure 26a. Hall effect sensors are positioned close to the wall to measure small changes in the magnetic field strength, as shown in Figure 26b, caused by the presence of minor defects.

**Figure 27:**
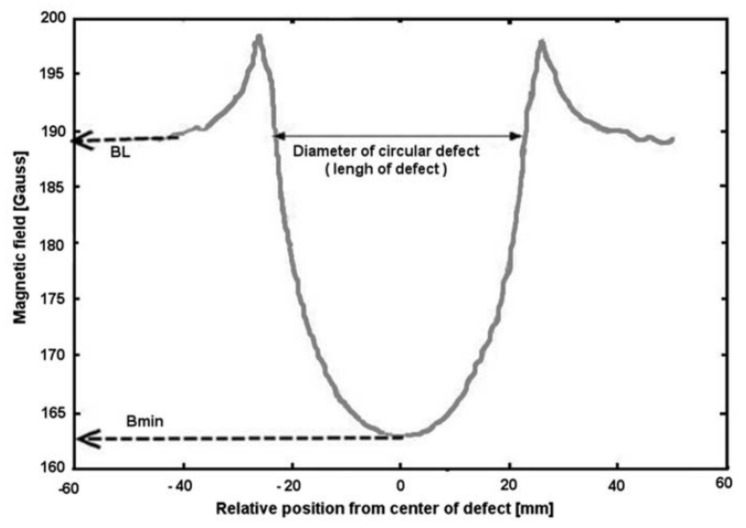
The visualisation of the dimensions of a corroded area on the wall of a pipeline using ICS. Reprinted from NDT & E International, Volume 42, Gloria, N.B.S.; Areiza, M.C.L.; Miranda, I.V.J.; Rebello, J.M.A., Development of a Magnetic Sensor for Detection and Sizing of Internal Pipeline Corrosion Defects, Page 669–677, Year 2009 with permission from Elsevier [102].

By plotting the magnetic flux density measurements with respect to the position of the ICS sensor from the defect, the depth and length of a corroded area on the wall of a pipeline can be visualised as shown in Figure 27. Based on the results in Figure 27, it can be observed that ICS allows the depth and length of a defect of a pipeline to be estimated directly from the measurements obtained by the Hall effect sensors. The distribution of the Hall effect sensors of the ICS, as shown in Figure 25, allows the ICS to measure the magnetic field strengths of a large area of the wall of a pipeline to map the defect with sub-millimetre accuracies. For conventional MFL PIG, an exhaustive set of experiments have to be implemented to first establish relevant relationships between the amplitudes of the MFL, and the dimensions of the defects because the MFL measurements obtained by the Hall effect sensors do not carry any explicit information about the dimensions of those defects. The ICS approach is, therefore, computationally less costly, compared to the MFL approach. It is also important to note that the error of the measurements obtained by the MFL approach is dependent on the degree of saturation of the magnetic field flux, which is determined by the thickness of the wall of a pipeline. This error does not exist in the ICS approach, which operates with a significantly weaker magnetic field flux that is significantly below the saturation point. The use of weaker magnetic field flux for inspection opens the possibility for the use of electromagnets to reduce the size and weight of the MFL PIGs.

### 2.11. Pulsed Eddy Current (PEC) (Corrosion)

**Figure 28:**
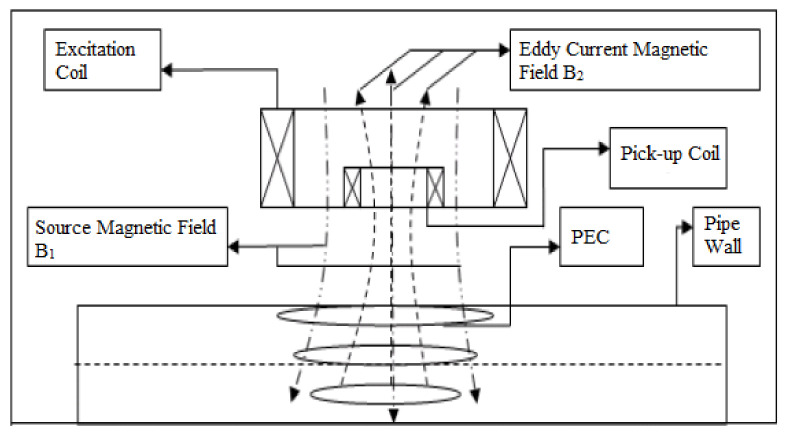
The schematic drawing of the PEC testing setup. Reprinted under Creative Commons CC BY license [103].

PEC was used in the detection of corrosion in insulated pipelines. The inspection of pipelines using PEC is non-intrusive since PEC is known to be able to penetrate the insulation coating of a pipeline. Conventional eddy current testing method employs Faraday’s law of electromagnetic induction to induce eddy currents in the wall of a pipeline via magnetic fields produced by sinusoidal electric current in an excitation coil. The induced eddy currents generate a quick-decaying magnetic field that is detected by the pick-up coil in the form of electric voltage [104]. The pulsed eddy current (PEC) testing, as shown in Figure 28, while employing a similar operating principle, uses electric current pulses with a broad frequency spectrum, allowing the eddy currents to penetrate different depths of the layers of the wall of a pipeline [103].

The pulsed voltage received at the pick-up coil of the PEC probe consists of the superposition of the signals produced by both the coating and the wall of a pipeline. However, the pulsed voltage attributed to the coating decays much faster, due to the low thickness of the coating. Therefore, the pulsed voltage measured at the receiver is mostly contributed by the eddy currents from the wall. By analysing the characteristics of the pulsed voltage, such as the amplitude, rising time, falling time and decaying rate, the change in the electrical conductance and magnetic permeability of the pipeline can be determined. These changes are relative to the change in the thickness of the wall of a pipeline.

In [104], the negative voltage pulses (negative peaks due to opposing effect of the current produced in the pick-up coil during electromagnetic induction) detected at the pick-up coil varied with different values of thickness of the wall of a pipeline. It was observed that the amplitude of the voltage pulse decreases while the decaying rate of the pulse increases as the thickness of the wall of the pipeline decreases. With a similar experimental setup as that in [104,107] employed the Fourier transform of the power spectrum density of the received pulse to achieve the same outcome. The challenge in the use of PEC to accurately determine the thickness of the wall a pipeline was investigated in [103]. It was found that the selection of the excitation strength of the PEC transducer directly affects the sensitivity of the transducer towards the changes in the thickness. There is a need for a compromise to ensure that the amplitude of the excitation pulse is sufficiently low to achieve the desired precision for a specific thickness while also being sufficiently high to create a magnetic field that is strong enough to penetrate the wall. In [105], this limitation is addressed by proposing the use of a differential probe technique, involving the use of two Hall effect sensors. The two sensors are located at the top and bottom of the axial centre of excitation coil. Any changes in the thickness of the pipe wall are evaluated by amplifying the small difference between the received signals measured by the Hall effect sensors, respectively. This approach by [105] is capable of differentiating thickness with a sensitivity of 1 mm.

PEC testing can be seen as an alternative to MFL testing for smaller and aged pipelines that are prone to mechanical damage. While both MFL and PEC testing rely on the use of magnetism, the complexity of the computation, which mainly involves signal processing techniques, required by the PEC is lower because the pulsed voltage at the pick-up coil at the PEC probe is less susceptible to noise, compared to the direct measurement of the magnetic field strength using the Hall effect sensors installed on a MFL PIG. However, MFL PIGs can be used to inspect pipeline networks of varying capacities and lengths, unlike the PEC probe, which can only be used for localised inspection. A breakthrough in converting a conventional PEC probe to a PEC pipeline inspection gauge (PIG) with an inspection speed up to 8 m/s was achieved in [106].

### 2.12. Fibre Optic Sensing (FOS) (Leakage, Crack, Corrosion)

Fibre optic sensing is seen as a more effective but less economical alternative for the conventional shape sensing techniques, such as cameras, radio detection and ranging (RaDAR), and light detection and ranging (LiDAR). Fibre optic sensing detects changes in the shape of an object by wrapping the object with one or more optical fibres and monitoring the optical changes in the fibre as the shape of the object changes. In conventional optical fibres, which employ the principle of total internal reflection for the transmission of data, light travels at approximately 70% of its speed in vacuum [112]. In [113], the solid core of the conventional optical fibres, which is made of silica glass, was replaced with a hollow core to allow light to travel at 99.7% of its speed in vacuum. At such speeds, either at 70% or 97% of the full speed capacity of light, a single remote interrogator unit, which interprets the optical changes in the fibre based on the type of modulation used, is sufficient to serve an optical fibre of several kilometres in length. By increasing the operating optical wavelengths, the sensing range is proportionally increased at the expense of the achievable spatial resolution. At an operating wavelength of 1550 nm, the corresponding sensing range and spatial resolution are 30 km and 10 m, respectively. At an operating wavelength of 975 nm, the corresponding sensing range is 4 km, while the spatial resolution is 2 m.

Being flexible and thin with diameters ranging between 100 µm and 2 mm, optical fibres can be used to measure strain at locations that are inaccessible to conventional sensors. The use of optical transmission renders fibre optic sensing immune to external electromagnetic fields [113]. However, the placement of optical fibres for the monitoring of buried pipelines is physically challenging, making FOS mostly deployed in overground and underwater pipeline networks. While an interrogator is used to handle the complex computational processes in deciphering the information carried by the optical signals in the optical fibres, the device is relatively costly, compared to sensing networks based on embedded systems that are of similar scale.

Like all other sensing strategies, optical fibres are used to monitor structural changes at large scales in the form of distributed fibre optic sensors. There are generally three common sensing techniques: Rayleigh, Raman and Brillouin scattering. These techniques, which share similar sensing mechanisms, are capable of monitoring the physical integrity of a structure by measuring optical changes caused by the change in the physical contact between the optical fibres and the structure. The mechanism involves the analysis of the back-propagating light generated when a forward-propagating optical signal is fed into an optical fibre. The characteristics of the back-propagating signal reflect the changes in the surrounding environment of the fibre [114].

The Rayleigh scattering sensing technique is used to measure the effects on the propagation of light caused by external physical fields, such as stress and strain. Since Rayleigh scattering is attributed to the local fluctuations of the refractive index of a material caused by unevenness and changes in the density of a material, the Rayleigh scattering sensing technique applies well to the monitoring of the structural integrity of pipelines in the detection of strain and cracks on the external surfaces. The Raman scattering sensing technique is conventionally used in distributed temperature sensing since the intensity of the anti-Stokes Raman scattered component of the optical signal is sensitive to changes in the local temperature of the optical fibres. The Brillouin scattering technique is used to monitor changes in temperature and strain [113].

**Figure 29:**
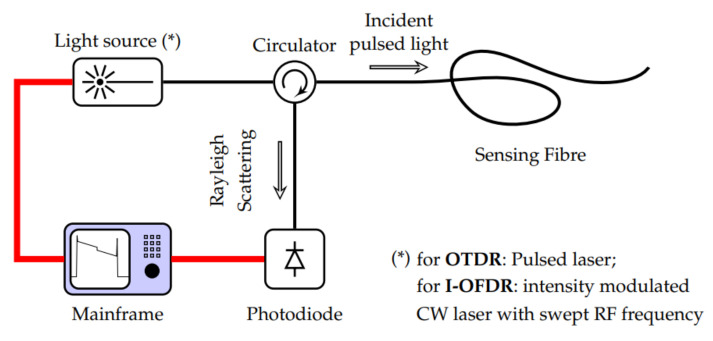
The principle of operation of the OTDR and I-OFDR schemes (CW—continuous wave; OTDR—optical time domain reflectometer; I-OFDR—incoherent optical frequency domain reflectometer; RF—radio frequency). Reprinted under Creative Commons CC BY license [114].

**Figure 30:**
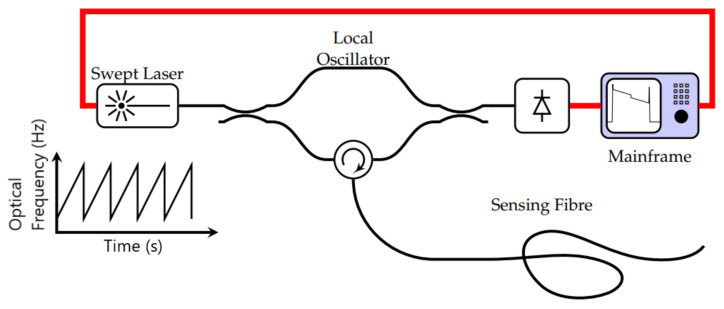
The principle of operation of the coherent OFDR scheme. Reprinted under Creative Commons CC BY license [114].

Two common interrogator units, based on the analysis of Rayleigh scattering, are the optical time-domain reflectometer (OTDR) and optical frequency-domain reflectometer (OFDR). The principle of an OTDR is as shown in Figure 29, where the forward and backward optical paths are separated after which photodetectors are used to measure the backward light intensity. By distinguishing the measured intensity values of the light pulses, the positions corresponding to the optical changes on the optical fibres can be determined. The principle of an OFDR, as shown in Figure 30, analyses the differences in the frequency characteristics for both the forward and backward optical signals.

**Figure 31:**
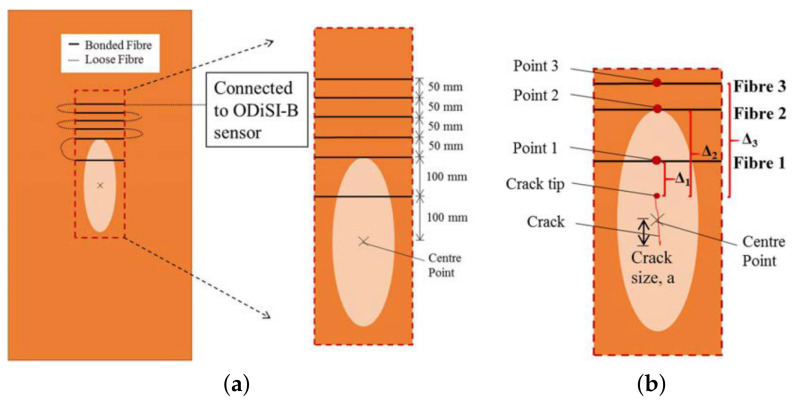
(**a**) Schematic drawing of the orientation of an optical fibre on the pipe specimen. (**b**) The position of the crack relative to the positions of the fibre rings. Reprinted under Creative Commons CC BY-NC-ND license [115].

**Figure 32:**
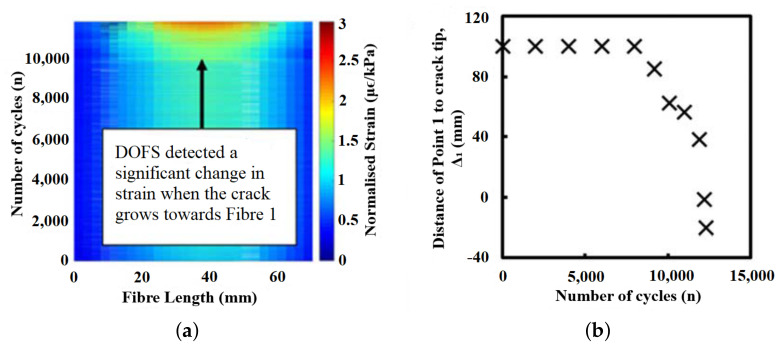
(**a**) The distributed strain measured along Fibre 1 during the cyclical pressure loading. (**b**) The relationship between the distance from the tip of the crack to Fibre 1 and the number of pressure loading cycles. Reprinted under Creative Commons CC BY-NC-ND license [115].

In [115], distributed fibre optic sensing (DOFS), using a OFTR based on Rayleigh scattering, the ODiSI-B optical distributed sensor interrogator from LUNA, was employed to measure the effect of internal pressure loading on the structural integrity of pipelines. Figure 31a shows how an optical fibre is oriented to form multiple fibre rings on the external surface of the pipe specimen. The same profile of pressure loading was repeated cyclically until a longitudinal crack was initiated. The position of the crack relative to the positions of fibre rings is as shown in Figure 31b. Based on the measurements recorded in Figure 32b, the tip of the crack grows towards Fibre 1 with significant nearness observed between the 10,000th and 12,000th cycles. The distributed strain spectrum in Figure 32a shows a corresponding gradual change in strain with a significant change recorded at the point when the tip of the crack is in contact with Fibre 1. This result provides evidence that DOFS can be used to detect the early onset of cracks, which is useful for preventive and predictive maintenance.

In [109,110], a Fibre Bragg Grating (FBG) strain sensor, which is a type of fibre optic sensor to detect changes in strain, was employed to detect the change in the diameter of a pipeline caused by the reduction in the pressure of the fluid in the pipeline when a leakage occurs. A FBG sensor is a specialised form of optical fibre. The refractive index of the core of the fibre is modulated according to the type of sensing required. The part of the core that is modulated with a varying refractive index is known as the grating.

**Figure 33:**
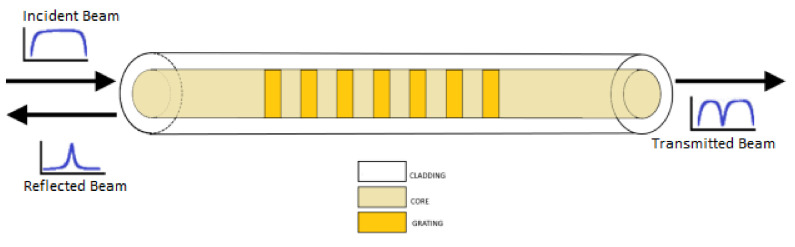
The schematic drawing of a simple FBG and the response spectra for the incident, reflected and transmitted beams. Reprinted under Creative Commons CC BY license [116].

Figure 33 shows the schematic drawing of a simple FBG. Since the modulated segments are equally spaced and have equal refractive indices, the refractive index along the grating, therefore, varies as a raised square wave. Based on the beams shown in Figure 33, the incident beam is reflected and transmitted due to the changes in the refractive index along the grating. When the incident beam is tuned to particular wavelengths that are defined by the type of modulation imposed on the grating, the Bragg condition is satisfied, where the reflected beam takes the form of a peak centred spectrum, while the transmitted beam is in the form of a notch filter. A change in the strain on the FBG is detected by the shift in the centre frequency of the peak response of the reflected beam [116].

In [108], a fibre optic sensor network was used for underwater natural gas pipeline leakage detection. A spectrum analysis of the optical signals was implemented using an optical time-domain reflectometer (OTDR). A wavelet transform was performed on the optical signals to increase the signal-to-noise ratio (SNR) to favour more accurate localisation of the leakages on the pipelines. Thermal monitoring using optical fibre sensors fibre optic sensor was implemented in [111] for the detection of leakages in oil and gas pipelines.

Distributed fibre optic sensing, which is widely in the detection of structural failure of pipelines, such as leakages and cracks, was experimented with in [5], using a setup similar to that shown in Figure 31 for corrosion monitoring. As mentioned earlier, optical fibres are sensitive to environmental parameters, such as strain and temperature. By analysing the spectral response of the Rayleigh backscatter of an optical fibre using the optical time-domain reflectometry interrogator, the distributed strain along the circumferential wall of a pipeline, also known as the hoop strain, can be acquired. The hoop strain is inversely proportional to the thickness of the wall of a pipeline.

**Figure 34:**
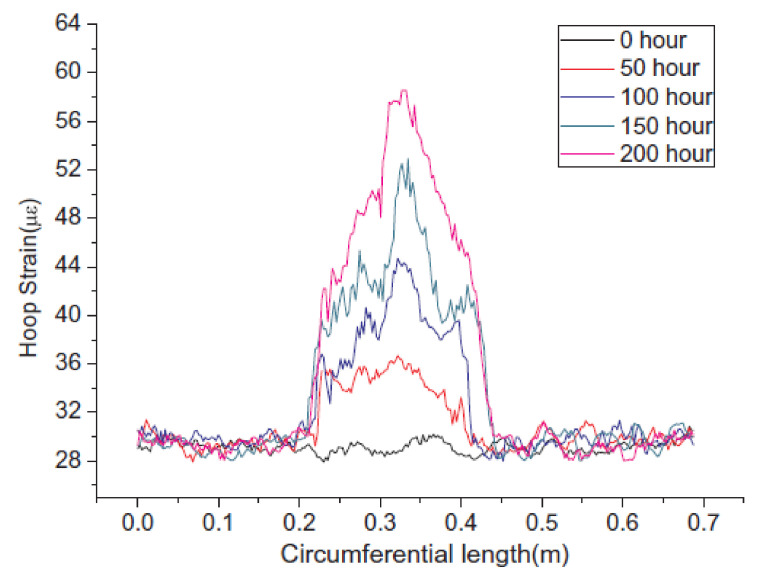
The hoop strain distribution of one optical fibre measured at a 50-hour-interval. Reprinted from Measurement, Volume 122, Ren, L.; Jiang, T.; Jia, Z.; Li, D.; Yuan, C.; Li, H. Pipeline Corrosion and Leakage Monitoring Based on the Distributed Optical Fiber Sensing Technology, Page 57–65, Year 2018 with permission from Elsevier [5].

Figure 34 shows the hoop strain distribution of a pipeline along the circumferential length of one optical fibre wrapped around the circumference of a pipeline measured at a 50-h interval as the laboratory-assisted corrosion in the pipeline develops. Based on the results in Figure 34, it can be observed that the peak strain measured by the optical fibre increases as the corrosion becomes more severe. The increase is caused by the progressive reduction in the thickness of the wall of the pipeline that results in a higher degree of deformation of the wall as pressure is exerted by the water flowing through the pipeline.

Fibre optic sensors, being sensitive to changes in the environmental fields, such as strain and heat, as well as being capable of capturing and relaying high-resolution data at the speed of light, are indisputably oriented for sensing applications of the future. However, despite the presence of various forms of interrogators to reduce the complexity of computation required in data processing, the practicality of FOS for the monitoring of underground pipeline is still lower than that of methods based on the analysis of hydraulic parameters, reflectometry and magnetic flux leakage.

### 2.13. Mobile Sensing/Robots/Drones (Blockage, Leakage, Crack, Corrosion)

The use of mobile sensing in pipeline failure detection is synonymous with the rapid development of embedded systems and the miniaturisation of various sensing devices, such as pressure transducers, ultrasonic transducers, lasers and cameras. Since most sensing devices are range limited, the use of robot technology increases the coverage of the scanning area of these devices, allowing them to be used for the inspection of large pipeline networks. The mobile sensing technology allows close-up monitoring of the internal environment of underground pipeline networks. Although most underground pipeline networks are accessible from above ground through inspection chambers that are normally positioned equidistantly, certain segments of a pipeline network are beyond the physical limit of a maintenance office, or even the range limit of sensing devices installed in the inspection chambers. Mobile sensing comes into play to overcome these limitations by tunnelling through the pipelines, using robots. However, it is important to note that the mobile sensing technology is not merely confined to the use of in-pipe sensing strategies, as the technology also includes the use of unmanned aerial vehicles, better known as drones, to perform inspections on the external surfaces of pipelines.

**Figure 35:**
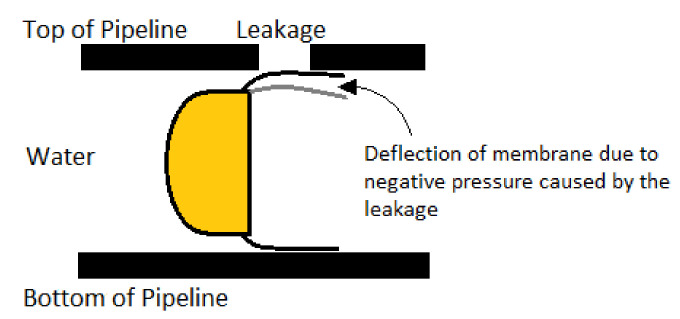
The deflection of the membrane on the detector of PipeGuard when a leak is present. Modified from [117].

In [27], an in-pipe robot, PipeGuard, employing the principle of pressure gradient analysis, was developed to detect the presence of leakages in gas pipelines. The robot consists of two parts: the carrier and the detector. The carrier handles the locomotion of the robot in pipelines, while the detector consists of a circular drum with a membrane that deflects, as shown in Figure 35, when pressure differences are present along the internal walls of the pipelines. As the robot travels along the internal environment of the pipeline, the change in the pressure gradient in the presence of a leakage causes the deflection of the membrane, resulting in the variation of the forces exerted on the drum of the detector; PipeGuard simplifies the sensing strategy used in conventional sensing strategies that are based on the use of an array of stationary hydraulic sensors installed along the internal wall of a pipeline. The simplification includes reduced maintenance effort and higher scalability. However, PipeGuard is not capable of real-time leakage detection since it performs only scheduled monitoring. A counterpart for the detection of cracks and corrosion in pipeline networks is a pipeline inspection gauge as proposed in [4,95] for the employment of magnetic flux leakage measurement, and [122] for the employment of ultrasonic waves. Since drones are becoming increasingly mainstream overground surveillance tools, Ref [3] proposed the employment of a drone to perform thermal imaging for the detection of leakages in underground pipeline networks.

**Figure 36:**
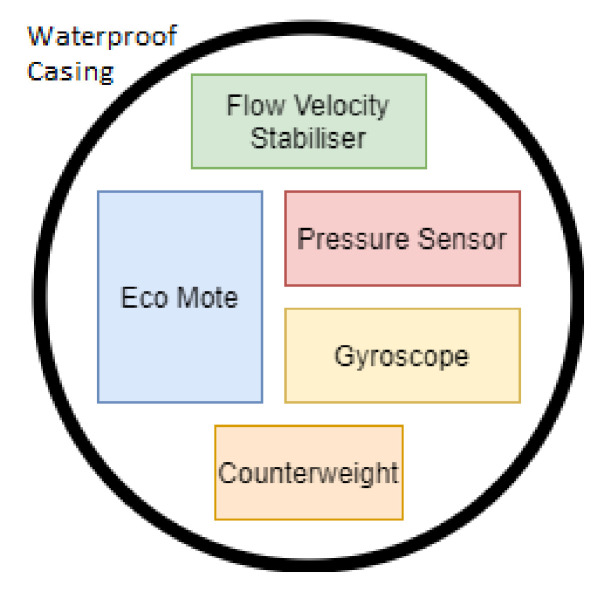
PipeProbe sensor capsule consisting of a waterproof plastic casing, a flow velocity stabiliser, Eco mote, a pressure sensor, a gyroscope sensor and a counterweight. Adapted from [28].

PipeProbe, a mobile sensor capsule technology introduced in [28], is able to reconstruct a 3D spatial layout of a pipeline system by collecting sensor readings. The motion of the sensor capsules is driven by the flow of water. Each of the sensor capsules, as illustrated in Figure 36, was designed to collect pressure readings as the capsules travel in the pipeline network. A median filter was then applied to the pressure readings to produce a smooth pressure graph. By analysing the trend and gradients of the pressure graph, the length of the straight segments and turning points of the pipelines were determined to finally produce a spatial layout of the pipeline network. The flow-driven sensing strategy of PipeProbe provides a monitoring means for hidden pipelines that are too small for in-pipe robots. Although [28] did not elaborate on the use of these capsules for the detection of water loss events, the potential exists for the pressure readings collected by the sensor capsules to be analysed, using the negative pressure gradient analysis proposed in [82,83] to detect water loss events in a pipeline network. However, the location data of the capsules corresponding to each pressure reading are required. If the capsules can be localised as they travel in the pipelines, PipeProbe stands out as a sensing strategy with higher deployability since it does not require the on-site installation of sensors. With the reduced number of sensors and embedded systems per deployment due to the mobility of the sensor nodes, PipeProbe is cost-effective, both computationally and economically.

**Figure 37:**
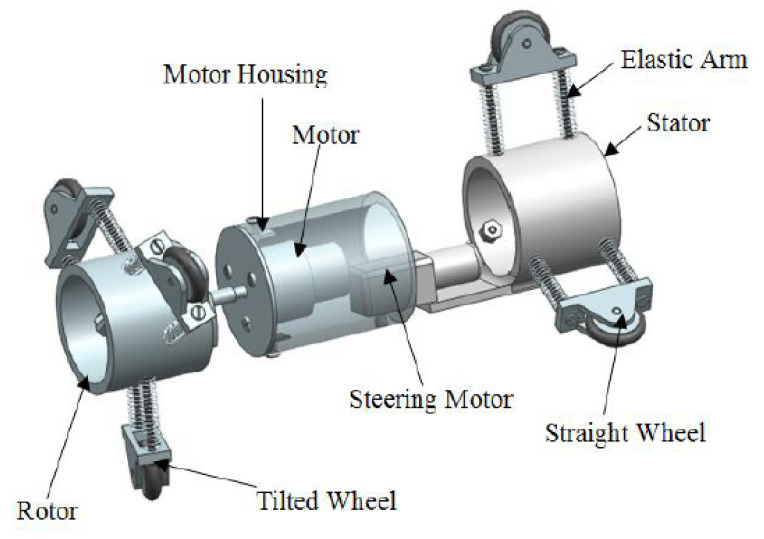
Solid model of a screw-drive robot, which is capable of moving along the x-axis, y-axis and z-axis in straight and curved pipelines. Reprinted under Creative Commons CC BY-NC-ND license [121].

The PipeGuard in-pipe robot proposed in [27] is part of the family of the screw-drive robots. Designs of screw-drive robots have been researched rigorously. For example, both [118,120,121] proposed the design of an actively steerable screw-drive in-pipe robot as shown in Figure 37, respectively. These robots can be manoeuvred to perform inspections in pipelines carrying different types of substances. The motivation behind the development of in-pipe robots that are capable of programmatic locomotion is the unsuitability of the use of free-flowing mobile sensing platform in pipelines carrying solid materials, such as those used in pneumatic waste conveyance systems (PWCSs).

Both fore-driving and rear-driving mechanisms are composed of three legs with a wheel attached at each of the legs. The wheels of both the fore-driving and rear-driving mechanisms are angled differently. The wheels of the fore-legs touch the internal wall of the pipeline at a subtle angle (approximately 15 degrees) to promote a helical motion of the front-driving mechanism as DC motor at the body of the robot rotates. The wheels of the rear-legs touch the internal wall of the pipeline at an angle of 180 degrees to promote a straight motion of the back-driving mechanism. The combination of the rotational helical motion of the fore-legs and the linear motion of the rear-legs allows the robot to tunnel through bent and curved pipelines. The robot design proposed in [120] has a steerable fore-driving mechanism, owing to an additional steering motor, which enables it is to manoeuvre through T-branches of a pipeline network.

**Figure 38:**
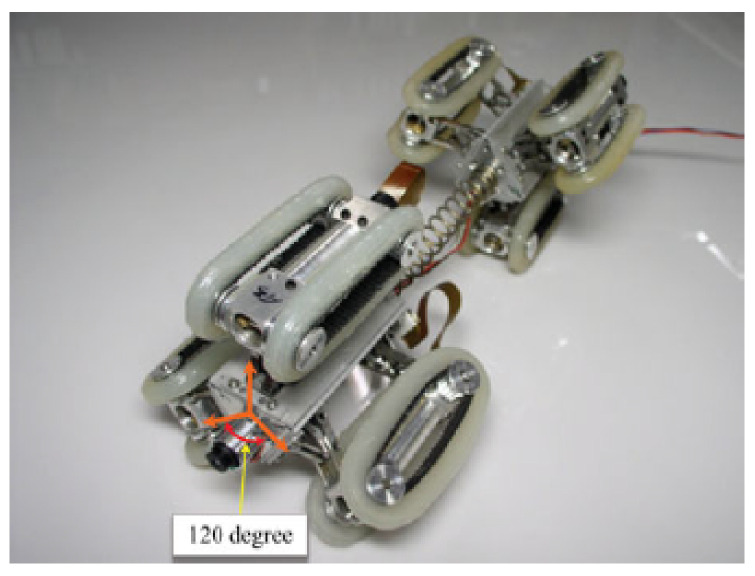
A two-segment in-pipe robot with caterpillar wheels. Reprinted under Creative Commons CC BY license [119].

**Figure 39:**
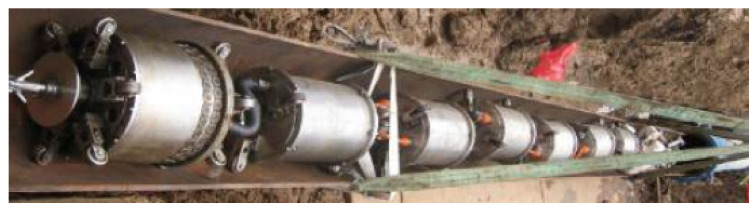
A worm-type in-pipe robots with multiple segments. Reprinted from Industrial Robot, Volume 37, Issue 2, Wang, Z.; Cao, Q.; Luan, N.; Zhang, L.; Meniconi S. Development of An Autonomous In-pipe Robot for Offshore Pipeline Maintenance, Year 2014 with permission from Emerald Publishing Limited [108].

There are other types of manoeuvrable robots, such as the caterpillar wheeled robots [119] and worm-type robots [108] as shown in Figure 38 and Figure 39, respectively. Robots with caterpillar wheels have a stronger grip on the internal wall of a pipeline, compared to robots with ordinary wheels. Worm-type robots have multiple segments, which can be equipped with various types of sensing devices, such as ultrasonic phased arrays and Hall effect sensors.

### 2.14. Process Tomography (Blockage, Leakage, Crack, Corrosion)

Process tomography is a common technique used in the pipeline industry to measure concentration distribution and flow phenomena in process pipes [123]. Generally, tomography employs the imaging by section principle through the use of a penetrating wave. This principle is used in computed tomography scan (CT), a common medical imaging technique. The use of process tomography in the inspection of process pipelines has been proven to be effective in the detection of blockages, leakages and cracks.

**Figure 40:**
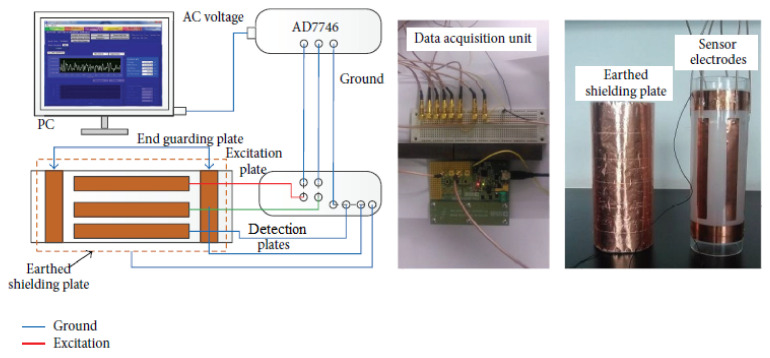
Schematic diagram and practical illustration of electrical capacitance measurement setup. Reprinted under Creative Commons CC BY license [32].

The study [32] proposed the use of electrical capacitance tomography to detect the presence of partial blockages in the form of wax deposition in non-metallic pipelines. In this work, a total of four wax models of different thickness of wax deposition were used to represent four different scenarios of partial blockage in a pipeline. For each of the scenarios, using the experimental setup as shown in Figure 40, the electrical capacitance at the location of each of the detection plates was measured as AC voltage was induced in the pipe segment through the excitation plates. The experimental results showed that the electrical capacitance measured generally increases as the thickness of the wax deposition increases.

**Figure 41:**
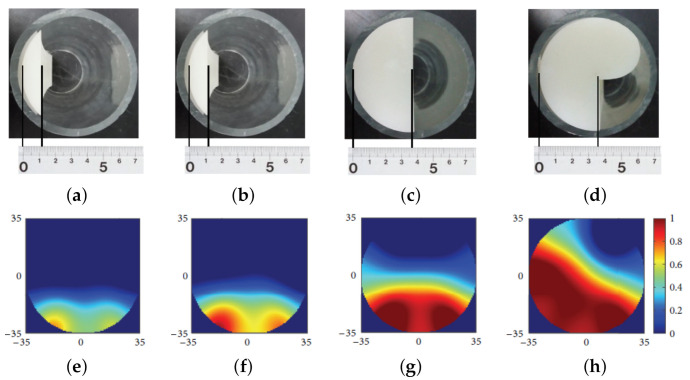
Actual wax model for deposits of (**a**) concentration = 11%, (**b**) concentration = 18.5%, (**c**) concentration = 52.5%, and (**d**) concentration = 62.5%. Reconstructed image for deposit (**e**) concentration = 11%, (**f**) cncentration = 18.5%, (**g**) concentration = 52.5%, and (**h**) concentration = 62.5%. Reprinted under Creative Commons CC BY license [32].

Using the electrical capacitance values collected in the experiment, Ref [32] attempted to reconstruct the image of the wax deposition in the pipeline, using the linear back-projection algorithm. Figure 41a–d shows the actual wax deposition models of different thicknesses of wax deposition. Figure 41e–h illustrates the reconstructed image of the wax deposition, using the linear back-projection algorithm.

The correlation of the wax deposit concentration between the actual wax model and the reconstructed image is as follows:

- 93.79% for actual concentration = 11%.
- 92.78% for actual concentration = 18.5%.
- 98.01% for actual concentration = 52.5%.
- 87.04% for actual concentration = 62.5%.

The results in [32] provided convincing evidence that electrical capacitance tomography is reliable in the detection of discrete wax deposit in non-metallic pipeline segments, especially in partial blockage conditions. However, the use of electrical capacitance measurement as the fundamental detection mechanism in this approach may not work in metallic pipelines, due to the presence of the shielding effect between the internal and external walls of a pipeline. Moreover, as wax deposits may be present in various locations in a pipeline network, although they are more commonly found at pipe bends and junctions, the use of electrical capacitance tomography requires the detection setup to be installed in multiple locations, which may incur an expensive deployment cost. Electrical capacitance tomography was also employed in [33], using a vastly similar approach as that found in [124], to measure the depth of defects in the form of cracks and grooves on the internal wall of a plastic pipeline.

In [124], magnetic induction tomography (MIT) was employed to detect the presence of metal loss events in a pipeline. This was implemented on the basis of measuring the change in the passive electromagnetic properties of a pipeline that is caused by the reduction in the metal mass due to the occurrence of corrosion. Eight voltage induction coils were uniformly distributed around the circumference of the external wall of a pipeline segment. By measuring the amplitudes of the induced voltages at different regions corresponding to the locations of the induction coils, the severity of metal loss events in the pipeline segment can be deduced based on the ratio between the amplitudes of excitation voltages produced by the coils and the amplitudes of the induced voltages. It was found that the ratios in the amplitudes increase as the severity of metal loss events increases. These ratios were utilised for the reconstruction of the image of the pipeline segment to provide a visual cue for the local corrosion condition. The narrow-band pass filtering method was used for the image reconstruction.

The MIT approach proposed by [124] addresses how process tomography can be used to evaluate the severity of metal loss events in a metallic pipeline. However, one major weakness of this method is that the granularity of the data obtained is highly dependent on the number of coils used in the MIT system. Due to design limitations, this method may only be suitable in the detection of general metal loss events in a pipeline segment. Discrete metal loss defects in the form of hairline cracks or small leakages may not be within the detection or sensitivity range of the MIT system.

**Figure 42:**
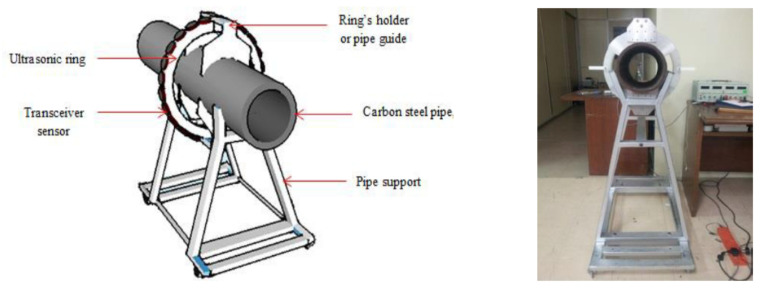
The setup for the ultrasonic tomographic instrumentation system. Reprinted under Creative Commons CC BY-NC-ND license [125].

The weakness in the MIT approach in terms of the sensitivity towards smaller metal loss defects was addressed in [125], which proposed the design and fabrication of an ultrasonic tomographic instrumentation system, as shown in Figure 42. A total of 28 ultrasonic transducers were mounted on the sensing ring at a distance of 50 mm from the the external surface of the pipeline segment. This mounting setup allowed each of the transducers to cover up to 20 mm of the circumference of the pipeline.

**Figure 43:**
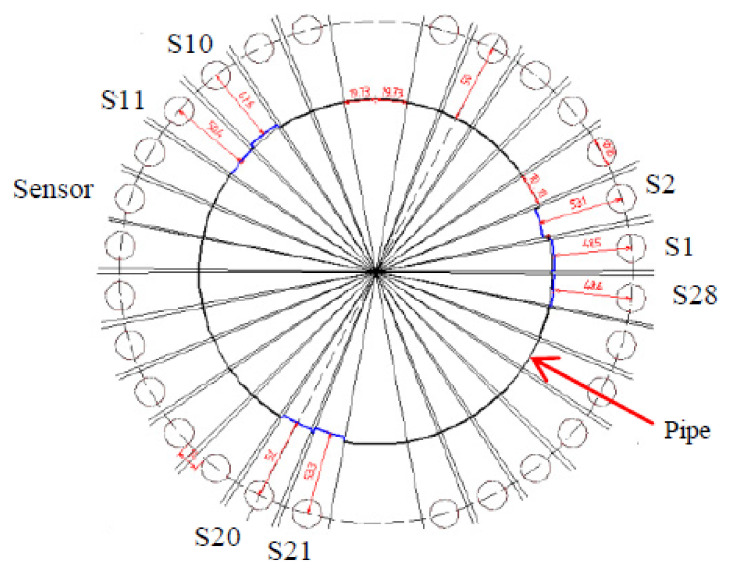
The reconstructed image of the cross-section of the pipeline segment at the location of the sensing ring. Reprinted under Creative Commons CC BY-NC-ND license [125].

Each of the transducers in the setup in Figure 42 was responsible for transmitting an incident ultrasonic wave towards the wall of the pipeline segment. The voltage of the reflected ultrasonic wave was then measured as a proxy for the thickness of the pipe wall corresponding to the location of the transducer. Using the voltages of the reflected ultrasonic waves at each of the transducers, an image illustrating the cross-section of the pipeline segment at the location of the sensing ring was reconstructed as shown in Figure 43. In the experimental scenarios used by [125], the ultrasonic tomographic tool was able to achieve an accuracy of 99.82% with the smallest measurable depth of 0.4 mm.

The method proposed by [125] is notably only capable of performing localised inspection, a common disadvantage shared by tomographic instruments, which can be observed in the methods proposed by [32,33,124]. However, it is important to note that process thermography methods contribute to the pipeline inspection industry by providing visual representations of the internal environment of a pipeline that are more perceivable for maintenance personnel through the employment of non-invasive techniques for data collection.

### 2.15. Radiography (Corrosion, Weld Defect)

Radiography is the use of radiation such as X-rays, gamma rays or any forms of ionising and non-ionising radiation to obtain the profile of a test specimen in the form of a radiographic film. The contrast on the radiographic film varies with respect to the varying penetration depth of the radiation at different parts or locations of a pipeline. Parts with higher penetration depth in a pipeline result in regions of higher contrast on the radiographic film [129]. Therefore, based on the measurement of the radiographic contrast between areas with the thinning of the wall and surrounding normal areas, the thickness of the wall of a pipeline can be evaluated. Like thermography, radiography is able to perform inspection of a pipeline without the removal of the insulating layer [126].

**Figure 44:**
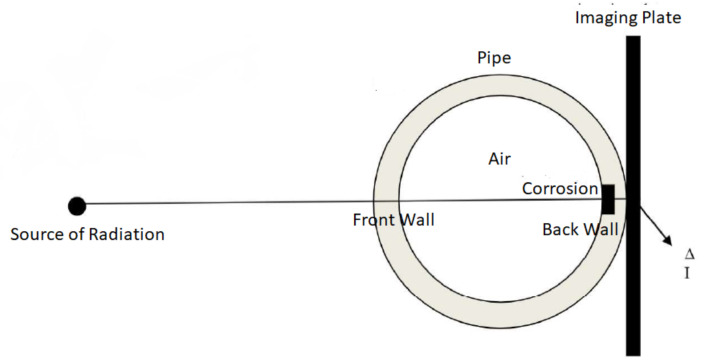
The setup for the evaluation of the thickness of the back wall of a pipeline using radiography. Reprinted under Creative Commons CC BY-NC-ND license [126].

In [126], a methodology was proposed, using a computerised radiographic system to evaluate the thickness of the wall of a pipeline based on the measurement of the contrast on a radiographic film with measurement errors smaller than 5%. Figure 44 shows the schematic drawing of the radiography setup for the evaluation of the severity of the thinning of the wall of a pipeline based on (ΔI), which is the difference between the intensity of the incident radiation at the source and the intensity of the radiation absorbed by the imaging plate. The operating principle of the setup obeys the Beer’s absorption law as expressed in the following:

(20) $$I=I_{0}\exp-\mu t$$

where I= intensity of radiation at the imaging plate, $I_{0}=$ intensity of radiation at the source, μ= total absorption coefficient of the media of transmission and t= the distance of transmission. Based on the setup in Figure 44, μ is the sum of the absorption coefficients of the first wall ($\mu_{pipe}$), the air in the hollow space ($\mu_{air}$) and the second wall of the pipeline ($\mu_{pipe}$), while *t* is the sum of the thickness of the front wall ($t_{fw}$), the diameter of the hollow space ($t_{air}$) and the back wall of the pipeline ($t_{bw}$). By measuring *I* of the radiation at the imaging plate and assuming that $I_{0}$, the thickness of the front wall of the pipeline ($t_{fw}$) and the diameter of the hollow space ($t_{air}$) are all constants, the thickness of the back wall of the pipeline ($t_{bw}$) can be determined by the following:

(21) $$t_{bw}=\frac{\ln\left[\frac{I}{I_{0}}\right]+\mu_{air}t_{air}}{-\mu_{pipe}}-t_{fw}$$

**Figure 45:**
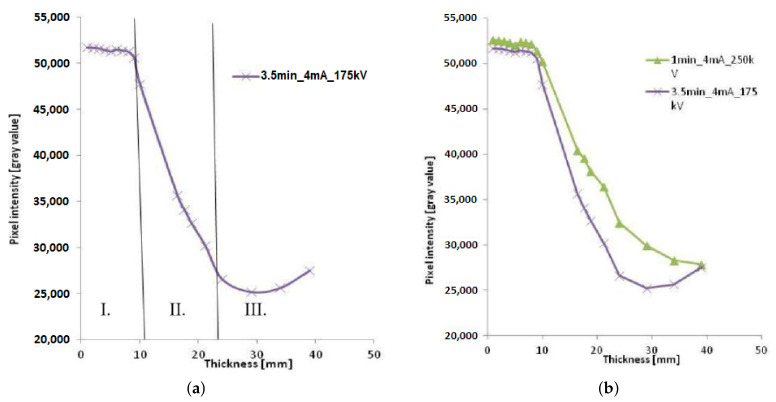
(**a**) The relationship between the pixel intensities of the radiographic film and the thickness of the wall of a pipeline at 175 kV tube voltage. (**b**) The comparison of the relationships between the pixel intensities of the radiographic film and the thickness of the wall of a pipeline at 175 kV and 250 kV tube voltage. Reprinted under Creative Commons CC BY-NC-ND license [126].

In [126], the relationship between the intensity of the radiation, the thickness of the wall of a pipeline and the pixel intensities of the radiographic film produced on the imaging plate was established. The authors also worked on the optimisation of the exposure parameters of the source of radiation to ensure that a linear relationship existed between the pixel intensities of the radiographic film and the thickness of the wall of a pipeline. The graph in Figure 45a shows the relationship between the pixel intensities and the thickness of the wall at 175 kV tube voltage. Based on the curve in Figure 45a, it can be seen that only a linear relationship exists at region I. The plateau in region I is caused by the saturation due to the radiation overdose when the thickness of the wall is small. The non-linearity in region III is caused by the scattering of the radiation, as the penetration depth of the radiation is severely hampered by the large thickness of the wall. The linearity in region II makes it the region of interest for the analysis of the radiographic contrast of a radiographic film based on the pixel intensities. The wideness of region II affects the range of measurable thickness of the CR system. When the thickness of the wall of a pipeline falls outside region II, the results obtained from the analysis of the contrast of a radiographic film are not reliable.

Figure 45b shows how the relationship between the pixel intensities and the thickness of the wall of a pipeline changes when the energy of the radiation source is increased. Based on Figure 45b, it can be observed that range of measurable thickness (region II) at 250 kV tube voltage is wider due to the reduced amount of scattered radiation as the thickness of the wall increases. However, there is also a decrease in the magnitude of the gradient of the curve corresponding to the 250 kV tube voltage. A smaller gradient signifies a lower achievable radiographic contrast for small changes in the thickness of a wall.

In radiography, the qualitative evaluation of the thinning of the wall of a pipeline is based on the comparison of the intensities measured in a radiographic test conducted on a specimen with the standard values of normal pipelines. The quantitative evaluation, on the other hand, is based on Equation (21), which requires all the constants to be accurately determined before the thickness of the back wall of a specimen can be determined. Radiography tests produce localised results and are often carried out ad hoc since the tests require the use of X-rays or gamma rays, which are hazardous. The instruments required for sourcing the radiation and capturing the radiographic images are also bulky and expensive. However, radiography is highly scalable since the power of the source of radiation can be adjusted to cater to pipelines of different wall thicknesses. However, the computational costs incurred for the use of radiography to evaluate the thinning of the wall of a pipeline is low since the severity of thinning can be gauged by a simple analysis on the intensity of the radiation captured in the form of a radiographic film on the imaging plate. This is unlike the use of radiography in the detection of bad welds, as explained in the next section, which requires intensive image processing to evaluate the conditions of weld defects.

**Figure 46:**
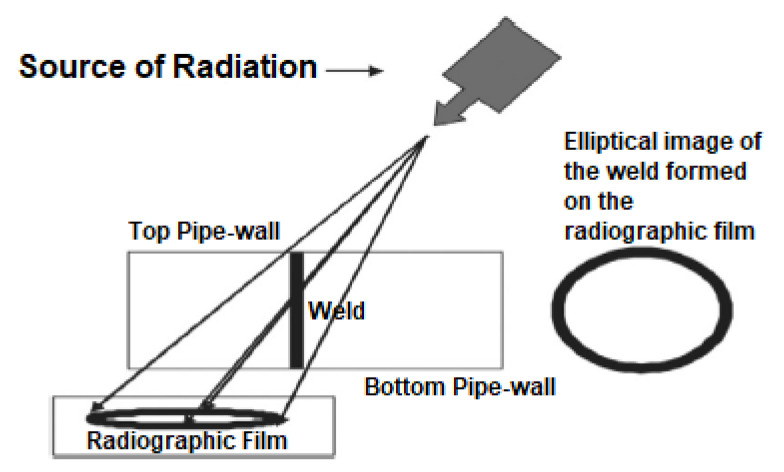
The operating principle of the DWDI technique. Reprinted from NDT & E International, Volume 86, Boaretto, N.; Centeno, T.M. Automated Detection of Welding Defects in Pipelines from Radiographic Images DWDI, Page 7–13, Year 2017 with permission from Elsevier [129].

The double-wall double image (DWDI) technique, as shown in Figure 46, is the most common radiographic technique used in the inspection of welded joints of pipelines. The term ‘double wall’ refers to the radiation penetrating through two walls of the pipelines. The term ‘double image’ refers to the superimposition of the radiographic images of both the upper and lower walls, resulting in an elliptical image. The double image is achieved by tilting the radiation source at an angle to avoid the overlapping of the images of the upper and lower welds of a pipeline [129].

**Figure 47:**
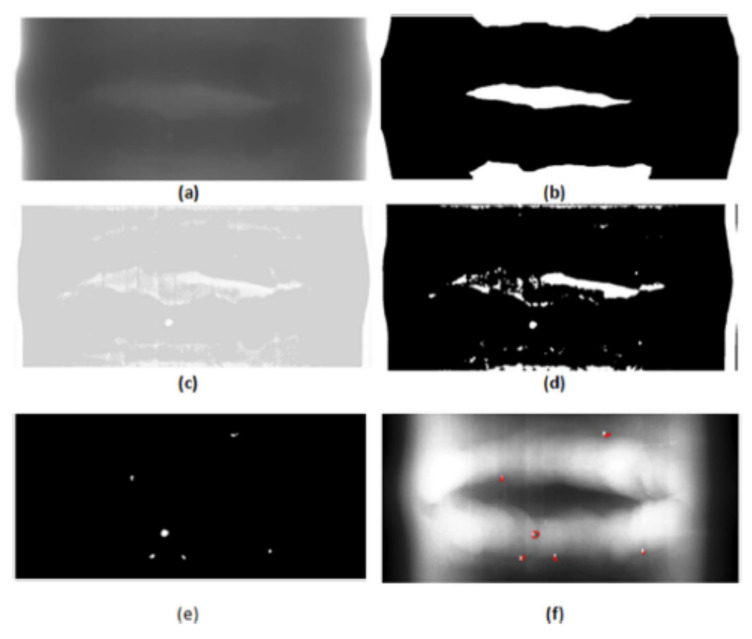
The image processing techniques applied to an original radiographic image of a weld prior to defect identification. (**a**) Clipping of the original image. (**b**) Thresholded image showing the region of interest. (**c**) Histogram equalisation. (**d**) Result of the combination of the image in (**b**) and the result of the Otsu thresholding of the image in (**c**,**e**); the revelation of potential defects and the region of interest and defects within 10% from the left/right and 7% top/bottom are discarded. (**f**) The labelling of defects after their edges are revealed by the morphological gradient method. Reprinted from NDT & E International, Volume 86, Boaretto, N.; Centeno, T.M. Automated Detection of Welding Defects in Pipelines from Radiographic Images DWDI, Page 7–13, Year 2017 with permission from Elsevier [129].

In [129], a feed-forward neural network with back-propagation learning was trained, using features extracted from radiographic films of the welds to classify the discontinuities in five classes: porosity, slag inclusion, lack of fusion, cracks and no defect. The trained network was able to produce an F-score of 87.5% in the dual classification of defects and non-defects. Prior to the extraction of features, the photographic films were subject to a series of image processing steps, such as adaptive Wiener filtering, average filtering, histogram equalisation and Otsu thresholding. Figure 47 shows how the original radiographic DWDI image of the top and bottom welds of a pipeline underwent several steps of image processing before the potential defects could be detected. Based on Figure 47f, which was the final result of a series of image processing techniques applied on the original image in Figure 47a, the extraction of features was carried out on the basis of such parameters as the area of the discontinuities, the eccentricity of the ellipse between the images of the top and bottom weld, the roundness of the ellipse, and the ratios between the major and minor axes. A similar image processing approach was implemented in [128].

In [127], the performance various architectures of deep neural networks (DNNs)—AlexNet, VGG-S, VGG-M, VGG-F, VGG-M-128, VGG-M-1024, VGG-M-2048, VGG-VD-16, and VGG-VD-19—in the detection of welds from the radiographic images of pipelines were evaluated. Since DNNs perform automated feature extraction without the need for human intervention, unlike the training of feed-forward neural networks that require the labelling of defects, the training time and the computational cost to train DNNs are significantly higher. It was found that DNNs are resilient, even in the presence of white and impulsive noise in the input radiographic images, with the VGG-VD16 being the best-performing architecture with an F-score of 96%.

Radiography, despite being effective in the detection of weld defects, poses several challenges for practical implementation in the industry, such as the high computational power required for image processing and the bulkiness of the radiographic devices. Like infrared thermography, radiography fulfils all four criteria: potential for quantitative measurement, being capable of real-time monitoring, being non-intrusive and being contactless. While efforts to reduce thermal cameras are ongoing, as seen in products such as the Lepton Flir to increase the compatibility of thermography with embedded systems for higher scalability and portability, the reliance of radiography on high-power radiation remains a massive challenge to tackle. The computationally intensive image processing techniques required for the identification of defects may demand the use of powerful computing platforms that are yet to be found among existing embedded systems.

### 2.16. Infrared Thermography (Leakage, Corrosion)

Every object emits electromagnetic heat waves in the form of infrared radiation. The temperature of the surface of the object determines the intensity of the infrared radiation. Therefore, an object with a surface consisting of points of different temperature values will radiate infrared rays of different values of intensity. The variation in the temperature on the surface of an object can be visualised, using an intensity spectrum of the infrared radiation emitted from the object [18]. The effectiveness of thermal imaging in detecting leakages and cracks depends on the difference in the thermal emissivity, heat capacities and temperatures of different surfaces and layers.

**Figure 48:**
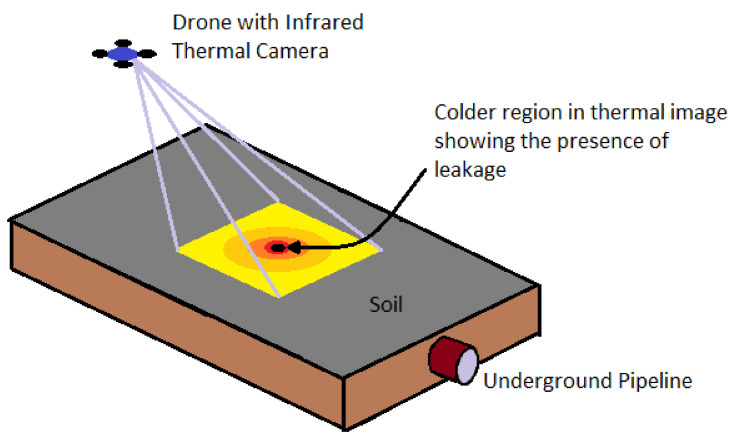
Thermal imaging of underground pipelines using a drone equipped with a thermal camera. Modified from [3].

In [3], infrared thermal imaging was used to detect leakages in underground pipelines. The underlying principle of the approach in [3] is based on the different absorption levels of the solar radiation by the pipeline itself, the fluid in the pipeline, and the environment of the pipeline. The soil above the pipeline has high temperatures since it is exposed to the solar radiation directly. Due to constant contact between the pipelines and the soil, the soil and the pipelines have almost equal temperatures, due to thermal equilibrium. Since the fluid in the pipelines is constantly flowing, thermal equilibrium hardly takes place, resulting in the fluid having significantly a lower temperature, compared to the pipelines and the soil. In a normal scenario when no leakage is present, the thermal camera detects a homogeneous intensity of infrared radiation. When a leakage occurs, the low-temperature fluid is exposed to the soil through the leaking orifice. By equipping a drone with a thermal camera, the thermal imaging of the underground pipelines in a large area can be carried out as shown in Figure 48. The presence of a leaking orifice can be detected through the distinguishable low-temperature region in the thermal image captured. In [18], the potential of infrared thermography in the field of corrosion monitoring was reviewed. The infrared thermography fulfills all four futuristic criteria of any corrosion sensing technique. The criteria are as follows:

- Direct measure of metal loss or corrosion rate.
- Real-time measurement with immediate results.
- No disruption to the services of pipelines during inspection.
- Distant and remote monitoring.

Infrared thermography exists in two categories: passive thermography and active thermography. In active thermography, apart from the use of a thermal camera to capture the thermal signature of the surface of an object, an external heat source that provides excitation to the surface is required. The characteristics of the heat source in terms of intensity and wave patterns can be adjusted to stimulate thermal evolution, such as heating and cooling of a surface. Passive thermography, on the other hand, involves the direct measurement of the thermal signature of an object, which is naturally at temperatures that are different from the background or surrounding temperature. In some cases, electromagnetic heat waves are propagated by objects that are under abnormal loading, such as fatigue, and impact or tensile loading. However, since passive thermography has no control over the thermal evolution of the surface of an object, it is only effective for the qualitative identification of abnormal temperature patterns or changes. Active thermography that allows the control over the heat states of the surface makes quantitative analysis, such as defect characterisation, possible.

In the field of corrosion monitoring of sub-surfaces, such as the internal walls of pipelines, the employment of thermography is not widely employed in the industry, especially for quantitative characterisation, due to numerous challenges that are inherent from the limitations of the technology itself. The limitations include the following:

- The thermal properties of an object, such as the variations in surface emissivity and reflectivity.
- The environmental conditions, such as non-uniform heating, variation in the ambient temperature, wind and humidity.
- The expensive cost of thermography devices for harsh operating conditions.
- The availability of energy for excitation in the case of active thermography.

Additionally, for simulation and experimental purposes, corrosion defects are often modelled as simplified cases, such as the flat-bottom holes or thickness variation often compromised of prior knowledge about the condition of the corrosion, when in reality, corrosion defects and the physics of heat are complex beyond the accuracy of the existing analytical models. This explains why thermography, despite being widely used for water and gas leakage monitoring, remains as only an alternative for existing state-of-the-art methods in corrosion monitoring.

Nevertheless, three common active thermography methods are used for the detection of corrosion or metal loss events. These methods are pulsed thermography (PT) [130], lock-in thermography (LT) [131] and step heating thermography (SHT) [132]. In PT, pulsed thermal energy in the form of heat is applied to the surface of an object, which results in instantaneous heating. Heat is then conducted from the surface of the object to the interior. The decay of the surface temperature is expressed as the following:

(22) $$T\left(t\right)=\frac{Q}{\rho CL}\left[1+2\sum_{n=1}^{\infty }\exp-\frac{n^{2}\pi^{2}}{L^{2}}\alpha t\right]$$

where *Q* is the heat deposited on the surface, ρ is the density of the object, *C* is the specific heat capacity, α is the thermal diffusivity and *L* is the sample thickness. Although Equation (22) only evaluates the temperature decay one-dimensionally, it, however, allows the thickness of an object to be determined with minimal computational complexity since all other unknown parameters in the equation are assumed to be fixed. By measuring the change in the thickness of an object, such as the wall of a pipeline over time, using the PT technique, the rate of corrosion or metal loss can be evaluated. Metal loss results in change of the thickness of a metallic object, which can be detected by measuring the variations in the temperature decay on the surface of the object.

**Figure 49:**
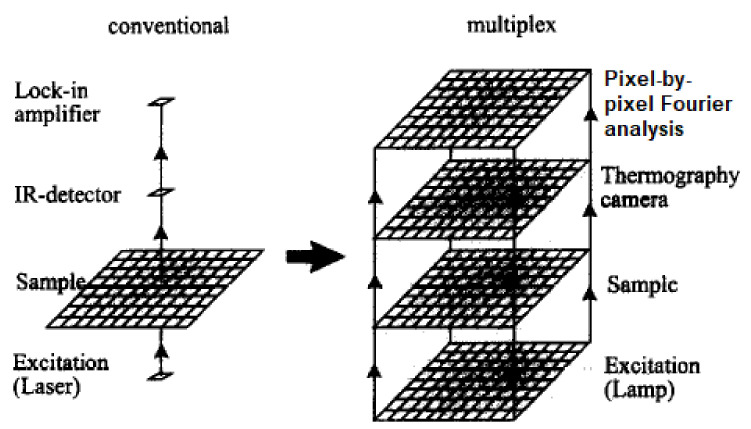
The schematic drawing showing the comparison of the setups for the photothermal radiometry and the lock-in thermography technique. Reprinted from Revue Generale de Thermique, Volume 37, Wu, D.; Buss, G. Lock-in Thermography for Non-destructive Evaluation of Materials, Page 693–703, Year 1998 with permission from Elsevier [131].

The LT technique is also known as phase-sensitive modulation thermography. The LT technique is derived from the combination of photothermal radiometry and infrared imaging. Photothermal radiometry allows for the remote measurement of heat transport in an object by measuring the phase angle between the remote optical energy deposition and the resulting thermal modulation. The use of infrared imaging allows fast detection of sub-surface or hidden defects. Figure 49 shows the schematic drawing showing the comparison of the setups for both the conventional photothermal radiometry and the LT technique. Based on Figure 49, it can be seen that photothermal radiometry relies on point-by-point raster scanning, which provides accurate measurements of temperature modulation at the expense of the long time needed to complete the scan of the sample. The LT technique, on the other hand, uses a wide-area excitation source and a thermal camera to scan the sample to obtain its surface temperature profile in pixels. A Fourier analysis is performed on the data of each of these pixels to obtain the magnitude and phase of the temperature modulation. Based on outcome of the Fourier analyses, localised changes in thickness can be determined through the characterisation of thermal wave fields of the sample.

The SHT technique outperforms the PT technique in terms of the detectable depth of a defect, which increases the effectiveness in the detection of embedded defects. Unlike pulsed heating in the PT technique, which uses a thermal stimulation pulse of high-power photographic flashes, the SHT technique applies heat continuously to the surface of an object for a long period. Due to the decaying nature of a pulse, the PT technique requires pulses that are of higher amplitudes as the thickness of the sample increases. High-power pulses may cause damage to the surface of the sample. The SHT technique, on the other hand, uses low-intensity uniform heat source to allow heat to penetrate the sample at a slower rate. For the SHT technique, the relationship between the temperature at point *X* and the thickness of the sample is expressed as the following:

(23) $$T\left(X,t\right)=\frac{2\sqrt{\alpha t}Q}{\kappa }\left[ierfc\frac{L(1+2n)-X}{2\sqrt{\alpha t}}+ierfc\frac{L(1+2n)+X}{2\sqrt{\alpha t}}\right]$$

where *T* is the temperature, *t* is the time, α is the thermal diffusivity, κ is the thermal conductivity of the material of the sample, *Q* is the heat flux, and *L* is the thickness of the sample.

In infrared thermography, the computational steps required for the qualitative analysis of corrosion are relatively simple since there is no need to accurately determine the amount of metal loss, apart from some direct image processing techniques to determine whether corrosion defects are present or otherwise. However, if the objective of a thermography inspection is focused on achieving accurate quantitative characterisation of corrosion defects instead of image processing, the computational steps involve intensive signal processing, machine learning and mathematical modelling to accurately predict the quantitative metrics of metal loss or corrosion defects. Another concern that was mentioned earlier is how active thermography helps in realising the quantitative characterisation of corrosion and also how its passive counterpart has much lower potential in doing so. The deployability of active thermography is limited since it requires an excitation heat source that may not be viable unless small transducer modules are designed to be equipped on robots or drones.

### 2.17. Optical Inspection (Blockage, Leakage, Crack, Corrosion)

The deployability of optical inspection of the internal environment of pipelines using closed-circuit television cameras has always been constrained by the lack of visibility in the interior of pipelines and also the poor quality of the images obtained due to poor lighting conditions [133]. If these constraints can be overcome, optical inspection has the potential to detect cracks, leakages and corrosion defects, such as pittings, in pipelines since cameras are able to collect visual information in the form of images or videos, unlike methods based on MFL, hydraulic parameters and vibrations, which employ sensors that are prone to interference and noise.

**Figure 50:**
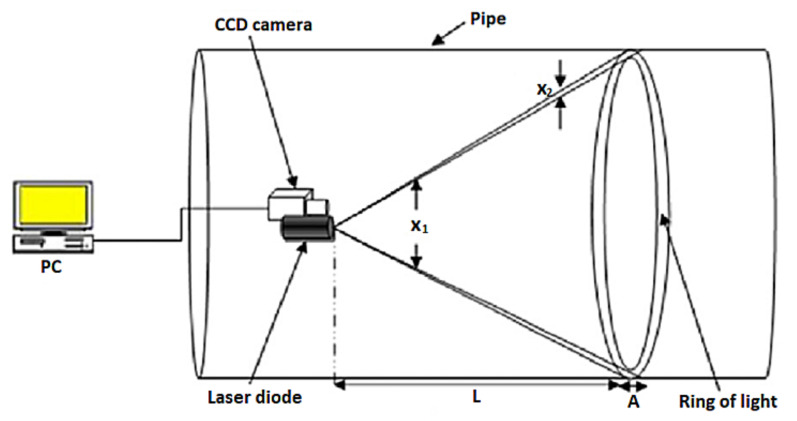
The projection of the laser beam in the form of a ring on the internal wall of a pipeline. Reprinted from NDT & E E International, Volume 52, Safizadeh, M.S.; Azzizadeh, T. Corrosion Detection of Internal Pipeline Using NDT Optical Inspection System, Page 144–148, Year 2012 with permission from Elsevier [133].

**Figure 51:**
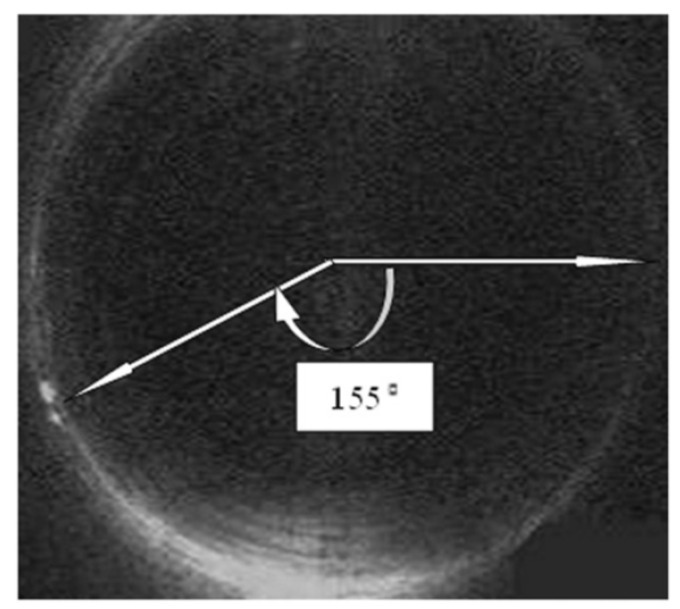
The ring profile of a pipeline segment showing the angular position of two ruptures. Reprinted from NDT & E E International, Volume 52, Safizadeh, M.S.; Azzizadeh, T. Corrosion Detection of Internal Pipeline Using NDT Optical Inspection System, Page 144–148, Year 2012 with permission from Elsevier [133].

The study of [133] was able to address the aforementioned constraints by proposing an intensity-based optical inspection method, which employs a CCTV-based laser profiler that combines a CCD camera, a laser-based optical diffuser and a ring projector. The ring projector expands the laser beam from the optical diffuser into a ring of light, illuminating the internal wall of the pipeline as shown in Figure 50. The variations in the intensities of the images captured by the light-sensitive CCD camera reveals information about the surface texture of the wall, thereby allowing the corrosion state of the wall to be evaluated. Light falling on areas with discontinuities on the surface of the wall is reflected irregularly, resulting in regions of higher intensities on the images captured by the CCD camera. Figure 51 shows the CCD image displaying the ring profile of a pipeline segment that is ruptured. By analysing the wall of a pipeline in the form of ring profiles, the image processing steps required becomes computationally simpler since feature extraction is performed only from the ring region of each image instead of having to focus on all areas of an image that requires a significantly higher computational cost. Since the features on the ring profiles for most defects are larger than 1 pixel, a scanning window containing a specified number of pixels was used to evaluate the local average intensity of different angular positions of the ring. The localised intensity ($I_{avg}$) is expressed as the following:

(24) $$I_{avg}=\frac{\Sigma I}{n\times m}$$

where Σ is the sum of the intensity values of all the pixels in the scanning window and n×m is the size of the scanning windows in pixels. Similar laser-based methods were proposed in [135,136]. The study [134] improved the design of the optical inspection device by including a parabolic reflector to increase the sharpness of the ring profile in which a defect measurement sensitivity of 150 microns was achieved.

Optical inspection, being an intrusive method, is often lacking in demand and doubted in the industry since the halting of the operations of the pipelines is required. Despite being intrusive, the deployability of the optical inspection methods are relatively high since the internal environments of various types of pipelines can be qualitatively analysed via direct optical profiling, using unmanned carriers, such as robots or drones since camera vision technology has achieved the maturity required for the miniaturisation and water-proofing of cameras.

### 2.18. Gamma-ray Transmission (Blockage)

**Figure 52:**
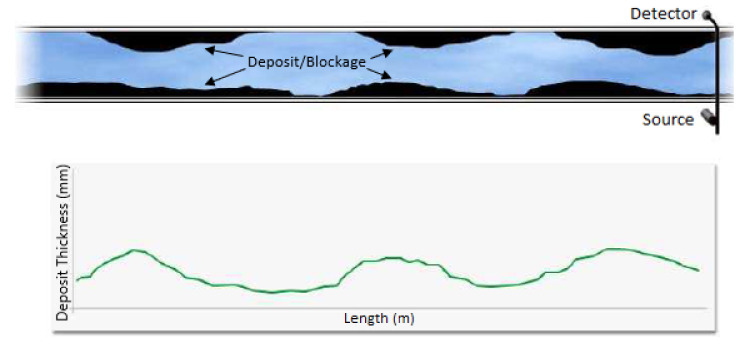
The setup for blockage inspection of a pipeline based on gamma-ray transmission and the corresponding measured blockage profile. Reprinted under Creative Commons CC BY license [137].

The gamma-ray transmission technique can also be used for the evaluation of the size of a blockage or deposit in a pipeline [137]. Gamma-ray transducers consisting of a gamma-ray source and a detector were fitted outside a pipeline diametrically as shown in Figure 52 to measure the transmitted intensity of the gamma ray across different sections along the length of the pipeline. While the computational strategy of this method that relates the the thickness of deposit on the wall of a pipeline to the intensity of transmitted gamma ray is relatively straightforward, the deployability of the sensing system based on gamma ray transmission is off limits for pipelines in the public environment since the use of gamma radiation for inspection may pose a safety hazard. In addition, the inspection is also localised to the part of a pipeline at which the sensing system is installed, limiting the deployability of the sensing system. The generality of the sensing system in terms of its suitability for pipelines with different operating parameters is relatively high since the detection thresholds of the gamma-ray detector can be easily tuned. The scalability of the system in terms of the size of the pipeline plant depends on the deployment cost available as well as the number of transducers required.

### 2.19. Vapour Sampling (Leakage)

**Figure 53:**
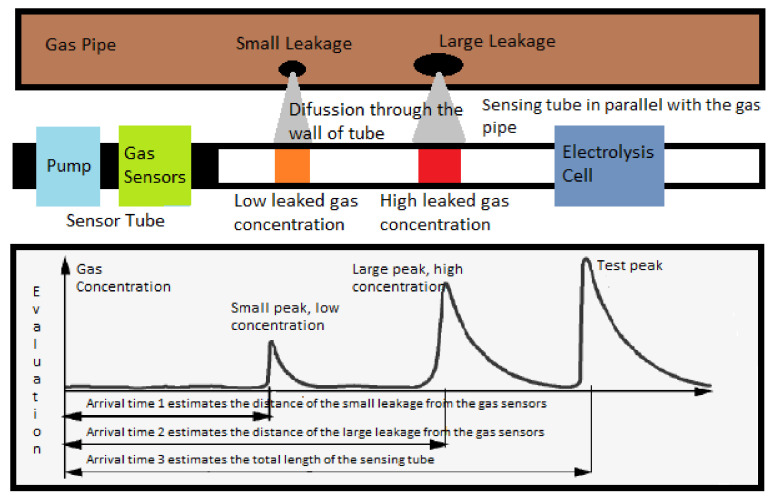
The schematic diagram of a vapour sensing tube by Framatome ANP Erlangen Germany for the detection of hydrogen gas leakages in a pipeline. Modified from [138].

Vapour sampling or sensing is used to detect leakages in pipelines carrying natural gases or other gaseous substances, such as hydrogen. In vapour sampling, the severity of a gas leakage is gauged based on the concentration or the volume of the gas that diffuses out of the pipeline through the rupture [138]. Figure 53 shows a sensing tube that is installed in parallel to the pipeline to perform either periodical or continuous vapour sampling on the air surrounding the pipeline.

The sensing tube is designed to be permeable to the substances to be detected by the vapour sampling system. It allows the diffusion of gases into the tube down the concentration gradient. In the event of a leakage, the leaked gas forms regions of high concentration in the air column inside the sensing tube. The pump connected at the end of the tube draws the air column towards the gas sensors, allowing the concentration profile of the air column to be evaluated. The end of the air column is marked, using a test peak of the gas to be detected. The test gas is normally produced using electrolysis prior to being injected into the sensing tube [138].

Due to the use of the principle of the diffusion of gases, which observes the presence of challenges, such as the rapid and random diffusion of gas particles, vapour sampling works best for short pipelines. Therefore, in order to ensure the accuracy in the localisation of leakages in long pipelines, the number of sensing tubes required for a vapour sampling network should be sufficient to have sensing coverage for all the pipelines in the facility. The number of gas sensing modules depends on the lengths of the sensing tubes. Due to the rapid changes in the gases’ concentrations due to the random nature of the diffusion of gas particles, the maximum length of the sensing tube served by each gas sensing module should be determined to both ensure true positive detection and avoid false negative detection of leakage events. The evaluation of the gases concentration employs only simple comparative computing based on standard concentration thresholds. The scalability of the vapour sampling technique is considerably high since the essentials of the sensing technique remain the same, regardless of the capacity of a pipeline network. Several vapour sampling networks for small pipeline plants can work together to address the sensing challenge in large pipeline plants. However, the number of sensing modules varies based on the safety requirements and thresholds of the pipeline facility.

### 2.20. Fluorescence (Leakage)

Hydrocarbon compounds in petroleum absorb ultraviolet light, resulting in the excitation of the electrons in the compounds. Due to the tendency of the electrons to return to their resting energy levels, the excitation is removed through the process of fluorescence emission of specific wavelengths, primarily in the visible region of the electromagnetic spectrum. Therefore, by looking for fluorescence signatures at these specific wavelengths, the presence of oil spillage from underwater oil pipelines can be determined [139].

The detection of oil spillage is performed by identifying fluorescence signatures at wavelengths between 400 nm and 650 nm. The identification process employs laser fluorosensors that use a laser excitation source that operates in the ultraviolet region of the electromagnetic spectrum between 300 and 355 nm. Since very few substances are able to fluoresce between 400 nm and 650 nm upon being excited by the ultraviolet rays, oil spillage can be easily distinguished by analysing the fluorescence spectra produced by the fluorosensors [139].

In [140], an unmanned surface vehicle equipped with a laser fluorosensor was used to detect oil spillage in the sea. The laser fluorosensor consists of a micro-pulse laser emitter operating at a wavelength of 337.1 nm and a spectrometer with a wavelength coverage between 338 and 736 nm. The results produced in [140] show that different hydrocarbons have their respective peaks of fluorescence intensity at different wavelengths, which allows each individual hydrocarbon to be easily distinguished from the rest. Additionally, the significant difference between the peak of the fluorescence intensity of water and that of the hydrocarbons makes the detection of oil spillage possible.

For certain gases, such as hydrogen, which are not detectable via laser-induced fluorescence due to the absence of absorption bands in the near-visible and visible regions of the electromagnetic spectrum, the analysis of the Raman scattering of the gas is employed. Like laser-induced fluorescence used for the detection of hydrocarbons in oil spillage, the Raman LiDAR also uses a laser excitation source operating at the ultraviolet band. Due to the use of an ultraviolet ray as the excitation wave to produce Raman scattering in the gas monitored, the analysis of Raman scattering is interfered by the laser-induced fluorescence produced by the substances surrounding the gas [141].

### 2.21. Electromechanical Impedance (EMI) Method (Leakage, Crack)

The electromechanical impedance (EMI) method is widely used in the monitoring of the structural health of various civil infrastructures, such as concrete structures, bridges, aerospace structures and steel structures. The measurement of EMI overcomes the insufficient sensitivity towards tiny cracks observed in methods employing the analysis of mechanical vibrations. While the state of the structural integrity is related to the mechanical impedance of the structure, the direct measurement of the mechanical impedance is challenging since such sensors do not exist. Therefore, instead of measuring the mechanical impedance of a structure directly, the electromechanical impedance is measured by employing the electromechanical coupling property of piezoelectric materials, which states that the changes in the structural mechanical impedance caused by the presence of cracks are directly related the variations in the electromechanical impedance measured at the interface between the piezoelectric material and the mechanical structure [142]. Therefore, in the EMI method, piezoelectric transducers are commonly used as both the actuators and sensors to generate high-frequency structural vibrations and detect the variations in the EMI, respectively [143].

**Figure 54:**
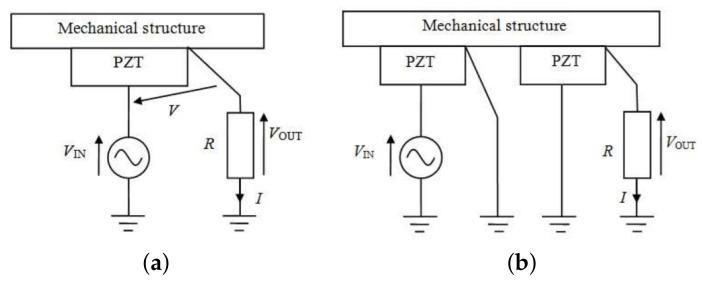
(**a**) EMI measurement setup using one piezoelectric transducer (PZT). (**b**) EMI measurement setup using two piezoelectric transducer (PZTs). Reprinted under Creative Commons CC BY license [143].

Figure 54 shows two common methods that are used to measure the EMI. In Figure 54a, the piezoelectric transducer (PZT) is used as both the actuator and the sensor simultaneously, where the EMI can be expressed as the following:

(25) $$Z=\frac{V_{IN}-V_{OUT}}{V_{OUT}}R$$

In Figure 54b, two PZTs are placed at two different locations on a mechanical structure, where one of the PZTs acts as the actuator, while the other PZT acts as the sensor. The EMI is this measurement setup can be expressed as the following:

(26) $$Z=\frac{V_{IN}}{V_{OUT}}R$$

**Figure 55:**
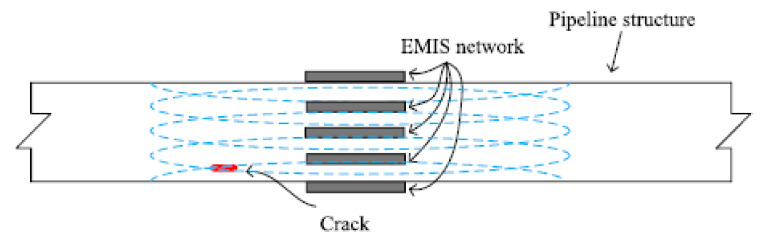
The schematic drawing of an annular setup of EMI sensors. Reprinted from Smart Materials and Structures, Volume 26, Number 10, Zuo, C.; Feng, X.; Zhang, Y.; Zhou, J. Year 2017 with permission from IOP Publishing Limited [144].

**Figure 56:**
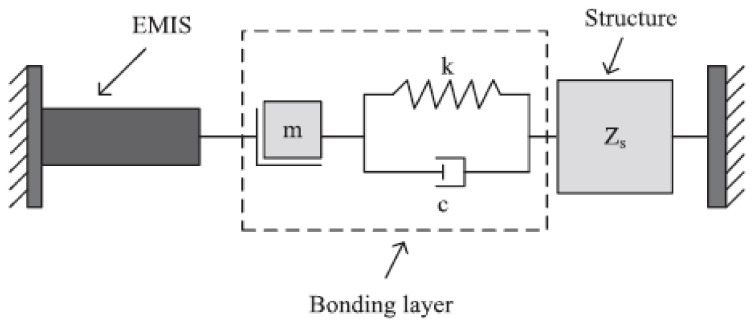
The modified one dimensional EMI model showing the bonding layer between the EMI sensors and the pipeline. Reprinted from Smart Materials and Structures, Volume 26, Number 10, Zuo, C.; Feng, X.; Zhang, Y.; Zhou, J. Year 2017 with permission from IOP Publishing Limited [144].

In [144], multiple EMI sensors (EMISs) were used, as shown in Figure 55, to enhance the spatial coverage of monitoring on the pipeline. Unlike the EMI models in Figure 54, the EMI model in [144] took into account the influence of the bonding layer between the EMI sensors and the pipeline as shown in Figure 56. By comparing the point frequency response plot (impedance versus frequency) measured by the EMI sensors on a test pipeline with the baseline plot obtained from a pipeline with no damage, using the deviation between the amplitudes of the resonant peaks sampled as the basis of comparison, the damage index of the test pipeline was computed for the ease of comparison between the severity of damage experienced by pipelines in the same network.

**Figure 57:**
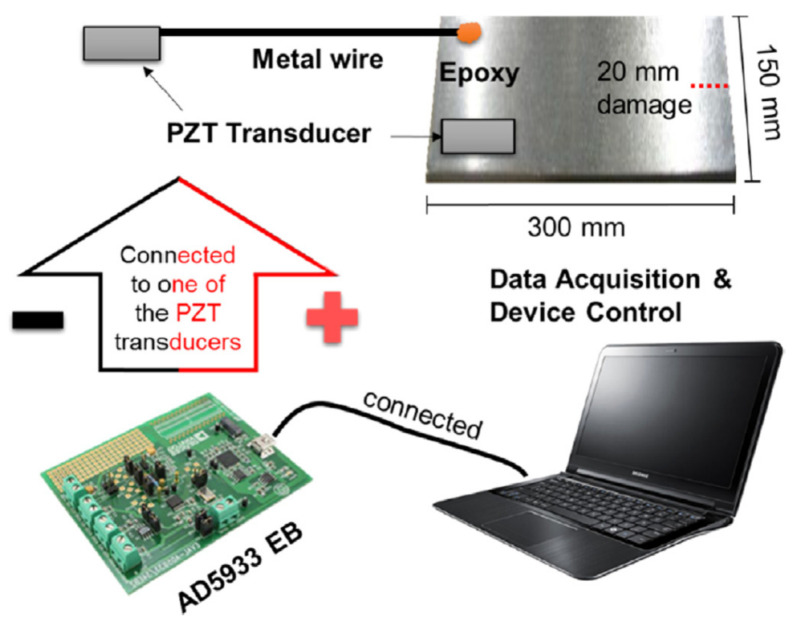
Using a metal wire as a bridge between the PZT and the epoxy coating of a pipeline for EMI measurement. Reprinted from Journal of Sound and Vibration, Volume 383, Na, W.S.; Lee, H. Experimental Investigation for an Isolation Technique on Conducting the Electromechanical Impedance Method in High-temperature pipeline facilities, Page 210–220, Year 2016 with permission from Elsevier [145].

However, the low curie temperature of piezoelectric transducers renders the EMI method unsuitable for the monitoring of pipelines carrying high-temperature substances. The heat from the pipelines changes the magnetic properties of the PZTs, resulting in the failure of the EMI method in the detection of changes in the structural integrity of the pipelines. The study [145] overcame this problem by using a metallic wire as a bridge between the PZT and the epoxy coating of a pipeline. Figure 57 shows the schematics of the experimental setup used in [145]. Although the method proposed in [145] was able to protect the PZT transducer from being subject to high temperatures, the sensitivity of the EMI measurement towards the detection of damage was reduced.

**Figure 58:**
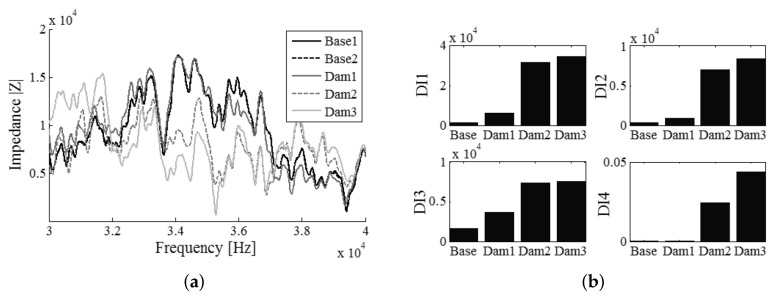
(**a**) The impedance versus frequency plots showing the shifts in the resonant peaks from those in the baseline plots for different damage locations. (**b**) The 4 sets of damage indexes calculated on the basis of different metrics. Reprinted under Creative Commons CC BY license [143].

The EMI method was also used in the damage assessment of the engine housings of jet planes [143]. Figure 58a shows the impedance versus frequency plots for several damage locations on the engine housings and the baseline plots, acting as the reference for comparison. Figure 58b shows four sets of damage indexes based on different computational metrics: DI1 [147], DI2 [146], DI3 [148], and DI4 [147], calculated from the analysis of the resonant peaks in Figure 58a.

The computing challenge in the employment of the EMI method is minimal since the calculation of the damage index of a mechanical structure is based on such metrics as root mean square deviation (RMSD) [146] and cross-correlation [148], obtained through the comparison between the frequency response plots of both the mentioned pipeline and its respective baseline counterpart. The use of statistical metrics, which are computationally straightforward in the EMI method, makes EMI sensor networks highly scalable. However, for a high-capacity pipeline network, due to the challenge in managing a large number of EMI sensors, these sensors should only be placed at locations that are prone to damage.

### 2.22. Electrochemical Impedance Spectroscopy (EIS) (Corrosion)

**Figure 59:**
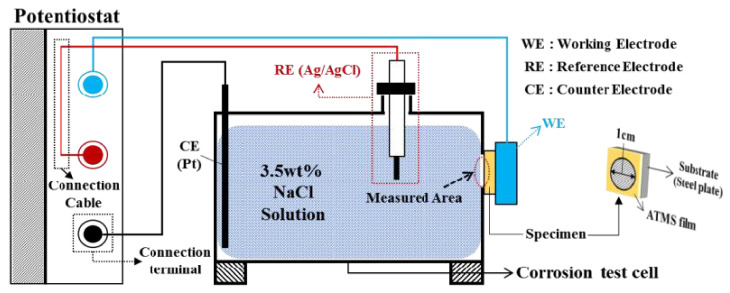
The schematic drawing of the experimental setup for EIS. Reprinted under Creative Commons CC BY license [149].

EIS is used as a laboratory-based inspection method to monitor the severity of electrochemical corrosion taking place on the wall of a metallic pipeline. Electrochemical corrosion is common in water pipelines due to the presence of reactive chemical compounds, such as bicarbonates and chlorides. Along with oxygen, these compounds induce the occurrence of cathodic and anodic reactions at the interfaces between the water and the metallic wall. These reactions result in the breakdown of the structural integrity of a pipeline through the replacement of strong metallic layers with brittle rust. Figure 59 shows an example of the experiment setup required to evaluate the corrosion characteristic of a metal specimen by varying the frequency of the AC electric potential between the reference electrode and the specimen. EIS is often performed offline since it is challenging to perform an in situ experiment using the setup shown in Figure 59. The corrosion characteristic of a metal specimen can be evaluated by measuring the impedance of the specimen over a range of AC frequency. The electric impedance measured for metals that are less prone to corrosion is generally higher due to the exhibition of higher polarisation resistance. The types of the working electrode, reference electrode and counter electrode vary according to the type of the specimen of an EIS test [149].

The EIS experiments performed in [150] validated that the presence of chlorides reduces the significance of the passivity phenomenon, resulting in the occurrence of structural breakdowns at a lower critical electric potential.

The computational complexity of the EIS is relatively low since the severity of corrosion is determined on the basis of measuring the electric impedance of the metal specimen and comparing the value measured with the reference value for the same type of metal. The scalability of the EIS depends on the homogeneity of the environmental conditions, such as aeration and moisture at different locations of a pipeline network, However, the outcomes of corrosion tests using EIS are localised since the specimens are randomly chosen from discrete locations of a pipeline network.

### 2.23. Corrosion Growth Modelling (Corrosion)

Corrosion growth modelling uses data from in-line inspection (ILI) techniques to estimate and predict the rate of metal loss of corrosion defects in pipelines to determine the regime for re-inspection and maintenance. Certain ILI techniques, such as methods based on magnetic flux leakage and guided ultrasonic waves, require periodical human intervention, which is expensive and at the same time, cause service disruptions since the methods are essentially intrusive. By modelling the growth of corrosion, the frequency of ILI of pipelines can be reduced since the future corrosion state can be estimated. The degree of certainty of the estimation, however, depends on the type of model used.

The fundamental of corrosion growth modelling matches the data measured over time from one ILI technique to another to determine the location and characteristics of the defects in a pipeline. Based on the matched defect features, the corrosion growth path for each local defect is determined to generate projections about metal loss over time. From the perspective of computing, the effort of matching the defect features from a different in-line inspection technique, better known as sensor fusion, is relatively simple if the number of defect features and the variations are limited. In most scenarios, such as in water pipelines, the number of defect features is large and increasing over time, resulting in the generation of large data that are computationally intensive to handle.

Therefore, in order to overcome this limitation, a stochastic corrosion growth model was proposed in [151]. The model in [151] treats the defect features provided by a single event of data collection from in-line inspection techniques as a population of defect sizes with a distributed frequency of defect sizes. By comparing the distribution of defect sizes for subsequent events of data collection, the increase in the severity of corrosion events in a pipeline network can be determined.

**Figure 60:**
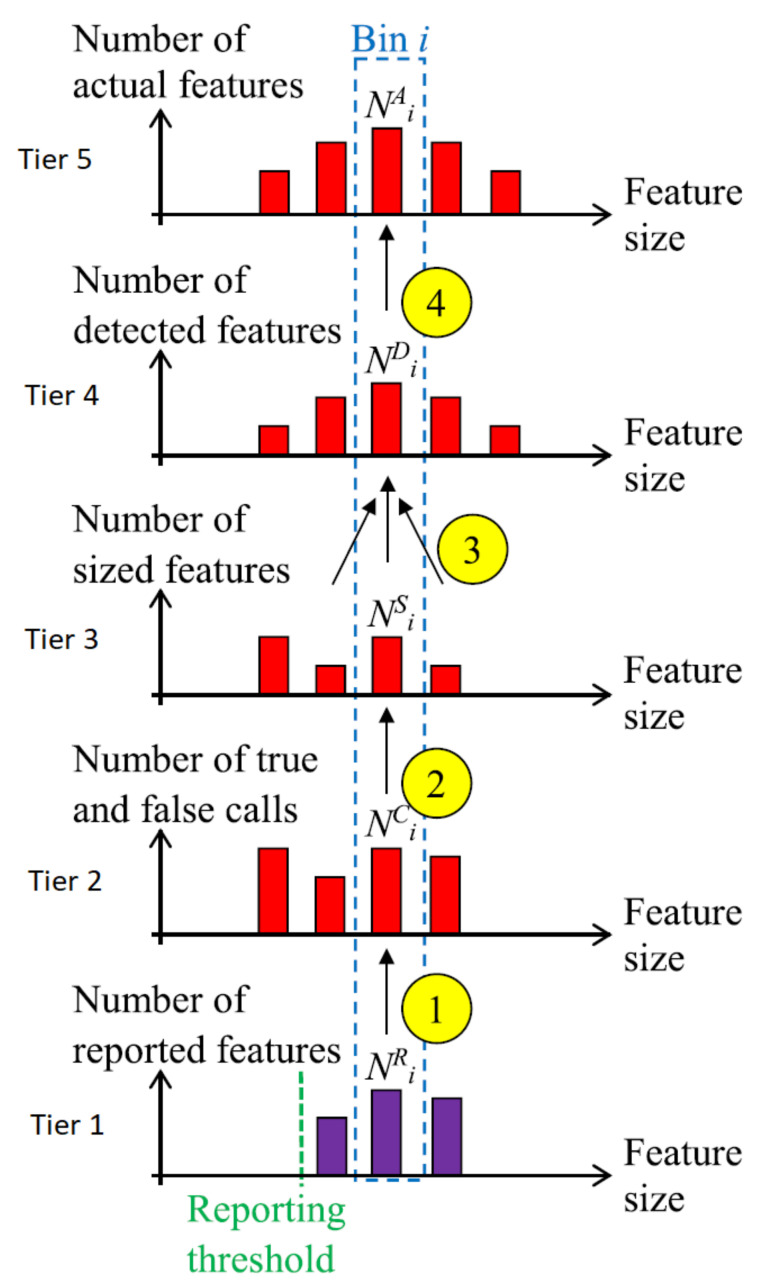
The hierarchical process (bottom to top) to determine determining the distribution of the number of actual features in histograms (each group of features from the similar range of feature size is represented in a bar, which is referred to as a bin) from the number of reported features provided by the data from in-line inspection techniques. Reprinted from Reliability Engineering & System Safety, Volume 180, Dann, M.R.; Maes, M.A. Stochastic Corrosion Growth Modelling for Pipelines Using Mass Inspection Data, Page 245–254, Year 2018 with permission from Elsevier [151].

**Figure 61:**
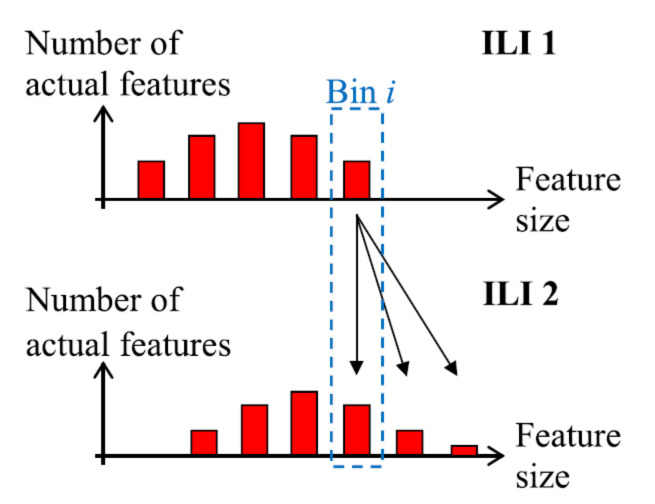
The shift in the distribution of the defects to the right is observed by comparing the distribution generated by two successive in-line inspections (ILI1 and ILI2). Reprinted from Reliability Engineering & System Safety, Volume 180, Dann, M.R.; Maes, M.A. Stochastic Corrosion Growth Modelling for Pipelines Using Mass Inspection Data, Page 245–254, Year 2018 with permission from Elsevier [151].

The example in Figure 60 shows how the actual metal loss in a pipeline network is determined. At tier 1, the range of the feature size provided by the ILI techniques is limited by the reporting threshold (sensitivity and measuring range of the sensors). At tier 2, the number of reported features provided by the ILI consists of both the number of true and false calls. In addition, from tier 2 onwards, it is assumed that the non-reported features are also provided by the operator of the ILI. Therefore, an extra bin on the most left position of the histogram is seen at tier 2. However, if the non-reported features are not available, the model should be conditional, as the non-reported results from ILI do not exist. Using the binomial probability theorem, the number of true calls (sized features) is determined. At tier 3, the number of true calls from tier 2 is now annotated as the number of sized features. By redistributing the false calls due to a sizing error to other bins, assuming that the feature size follows a normal distribution, the number of detected features is now distributed as shown in this histogram at tier 4. At tier 5, the frequency for each bin is adjusted by including the number of undetected features, determined also by using the binomial probability theorem. By comparing the distributions of actual defects generated using data from two successive ILIs, the progression of corrosion in a pipeline network can be determined as shown in Figure 61.

The same author also proposed a framework for automated feature matching for the determination of corrosion growth in [152]. In this framework, the complex 3D multi in-line inspection problem was simplified into multiple instances of a 2D point matching problem, which allows the time taken for the computation of defects to be minimised. After the simplification, a parametric probabilistic model, based on three parameters—α to control the number of outliers, β to control the transformation close to identity, and $T_{final}$ to control the probabilistic correspondence assignment—was used to match feature points from two in-line inspection instances. Matching feature points with a probability higher than 0.9 from two instances of inspection are considered for the corrosion growth analysis for the development of corrosion growth models.

The corrosion model in [153] incorporates the use of Monte Carlo simulations for the estimation of the probability distributions of maximum pit depth and growth rate with respect to different soil conditions and the use of extreme value distributions—Weibull, Fréchet or Gumbel—to fit the distribution of these corrosion attributes. At the expense of higher computational costs, Monte Carlo simulations greatly reduce the complexity and the cost of laboratory studies required to determine the long-term distribution of corrosion tributes in different soil environments. Additionally, using the average pitting depth from field data, it was found that Monte Carlo simulations were able to estimate the time-variant maximum pitting depth for different soil conditions with the worst accuracy of 12%. In [155], the effects of temporal variability of operating parameters, such as pH, flow velocity, temperature and partial pressure of gas in corrosion growth modelling, were investigated. The corrosion rate in [155] was predicted on the basis of electrochemical impedance spectroscopy, which was discussed in this report. It was found that the variations in these operating parameters affected the long-term corrosion rate by changing the electrochemical properties of the metal specimen. A dynamic model for pitting and corrosion-fatigue damage based on dynamic Bayesian networks (DBNs) was used in [154] to represent the temporal and varying growth rate of corrosion. Monte Carlo simulations were also used in [154] to analyse the time-variant growth of the size of a pitting defect to estimate the time taken for the defect to transition to a short crack.

The employment of corrosion models for the prediction of corrosion growth should be considered as a second to none alternative to predictions solely relying on the data from in-line inspections. Corrosion models are often computationally intensive since the size of the data required for accurate and reliable predictions is generated using computer simulations. However, the higher computational costs often offset the high maintenance and operating costs needed for in-line inspection techniques. There is also no concern about the scalability of corrosion models since adjustments can be easily made virtually to fit the requirements of pipeline networks of various capacities.

### 2.24. Distributed Cyber-Physical Systems (Leakage)

With the rapid development of smart devices that are capable of micro-sensing, on-board processing and communicating wirelessly, distributed cyber-physical systems (CPSs) based on wireless sensor networks (WSNs) are used indispensably in failure monitoring and reporting for distribution pipelines [159].

In [34], PipeNet, a pioneering water pipeline monitoring system based on a wireless network system was developed and field-tested in the city of Boston in the U.S.A. in collaboration with Boston Water and Sewer Commission (BWSC). PipeNet used Intel Mote sensor nodes to collect hydraulic and vibration data in underground water pipelines at a high sampling rate. The data from each sensor node were then transmitted using wireless UART to the nearest gateway. The gateways were responsible for relaying the date back to a backend server. The integer Haar wavelet transform and frequency content characterisation were employed to analyse the pressure and acoustic data, respectively, to detect leakages in the water pipelines. SmartPipes by [156] introduced a pressure sensing method based on force-sensitive sensors (FSRs) which detects the change in the diameter of a pipeline caused by the change in the internal pressure of the pipeline. The use of FSRs overcomes the difficulty in the installation of the conventional hydraulic pressure sensors in underground pipelines that have limited accessibility. In [157], while employing conventional hydraulic pressure sensors, the predictive Kalman filter was hybridised with the conventional time difference of arrival algorithm to detect water loss events in a water pipeline network. Instead of relying on multiple algorithms to separately filter, analyse and compress the pressure data, which may cause a severe degradation of the state of health of the battery, the predictive Kalman filter, which is both efficient and lightweight, was used to perform all three tasks.

**Figure 62:**
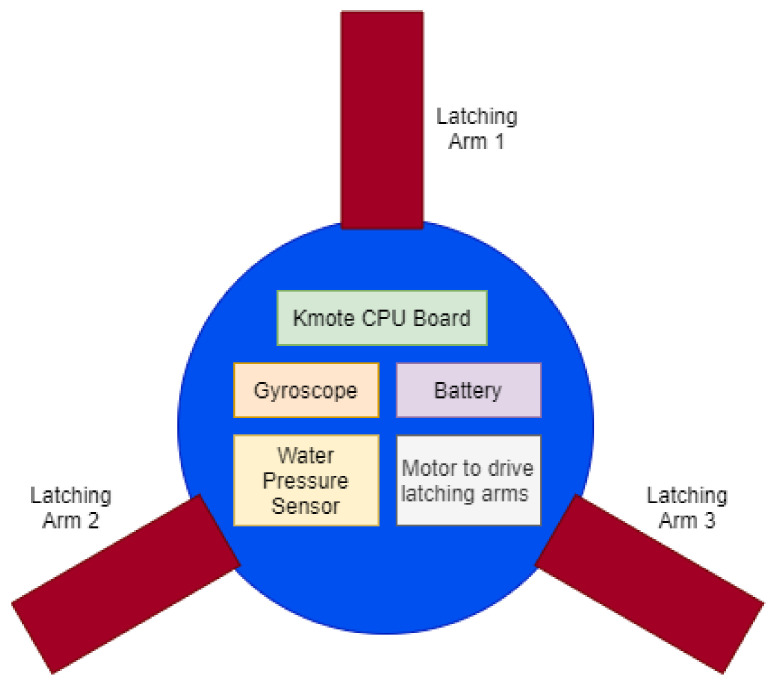
The prototype of TriopusNet sensor node. Adapted from [158].

The solution, known as TriopusNet, for automating the deployment and replacement of the sensor nodes in a wireless sensor network was proposed in [158] with the aim to reduce the number of sensor nodes required to cover a unit sensing area in a pipeline network without compromising on the state of the network connectivity among the nodes, which collect data at high rates. The TriopusNet node prototype as illustrated in Figure 62 consists of a sensing unit comprising of a pressure sensor and gyroscope and a mechanical latching unit consisting of three mechanical arms. The latching unit allows the sensor node to latch itself to the internal wall of a pipeline. The system design of TriopusNet resolved the order of the deployment of the sensor nodes by transforming the physical layout of a pipeline network into a virtual tree represented by a source, branch points, pipe segments and end points. The source is essentially the main faucet or the water reservoir of the pipeline network. The branch points are the intersection points between two or more pipeline segments. The end points are essentially the faucets of the end users of the network.

By running a post-order transversal algorithm on the virtual tree, the sensor nodes are deployed in the pipe segments in the following order by deploying the sensors at the pipe segments that are closest to the end points. By orchestrating the on–off moments of the endpoint faucets, the sensor nodes can be precisely carried by the flow of water from the source to their respective pipeline segments. The post-transversal algorithm allows the deployment of downstream sensor nodes to precede the deployment of its upstream sensor nodes to prevent the blocking of the deployment pathway of a newly released sensor node by the preceding sensor nodes. The TriopusNet also considered the replacement of sensor nodes that were depleted of battery charge by employing the collaboration between the battery-depleted nodes and good nodes. When a sensor node is depleted of battery charge, it unlatches itself from the wall of the pipeline and is flushed out of the pipeline network to prevent clogging. All the good nodes then move downstream to cover up the voids left by the departed sensor nodes. The sensor repository at the water inlet of the pipeline network subsequently releases fresh good nodes to cover up the upstream voids that are created by the movements of the good nodes.

**Figure 63:**
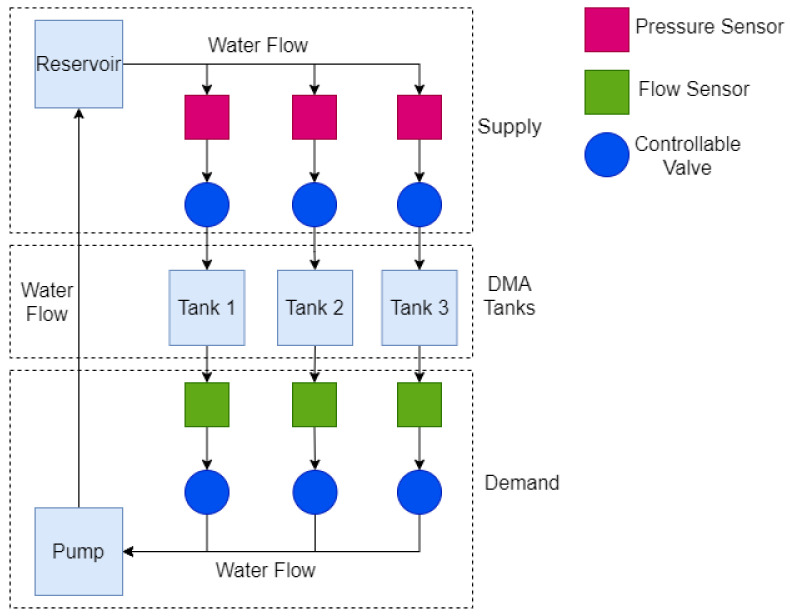
The schematic drawing of the WaterBox testbed. Adapted from [40].

A smart water management testbed, WaterBox [40], was developed for the monitoring and controlling of water distribution systems (WDS). WaterBox, as shown in Figure 63, is a small-scale testbed, consisting of three layers—supply, district meter areas (DMAs) and demand layers—to simulate a dynamic meter area DMA-based smart water network. The flexible hydraulic, hardware and software infrastructure enables the replication of dynamic events, such as leakages and bursts in water networks.

WaterWise was developed to enable real-time monitoring of water distribution network in Singapore. Eight water-proof sensor nodes consisting of a pressure sensor, a hydrophone, a flow meter, a processing board and 3G/Wi-Fi communication unit, were deployed to collect data across 60 km^2^ area of Singapore. The data collected from the sensors were used for visualisation, on-line event detection and on-line dynamic hydraulic modelling of the WDS in Singapore.

The data from WaterWise were used to demonstrate an efficient search algorithm in [35]. The algorithm merges the average distances between any two mobile sensor nodes in a water pipeline network with the difference in the arrival times of the pressure variations detected at the locations of the sensors to locate leakage events more effectively.

In [159], a smart WSN based machine learning method was used for the detection and size estimation of leakages in oil and gas pipelines. The performance of the WSN in the identification of leakages based on negative pressure waves using different machine learning methods—support vector machine (SVM), K-nearest neighbour, Gaussian mixture model (GMM) and naïve Bayes (NB) networks—was tested and compared. SVM was found to have the highest accuracy of leakage detection and sizing among all the methods. Similar works were also implemented in [25,160], where it was found that the accuracy of detection could be further improved, using the combination of SVM and Fisher discriminant analysis (FDA).

## 3. **Discussion**

Table 1 shows the various existing non-destructive methods along with their suitability for the detection of different pipeline defects. The failure detection methods covered in this paper are non-exhaustive and include, to the best of our knowledge at the point of writing, non-destructive technologies that were practically validated in the industry, either in the form of modern wireless sensor networks or human-operated devices.

**Figure 64:**
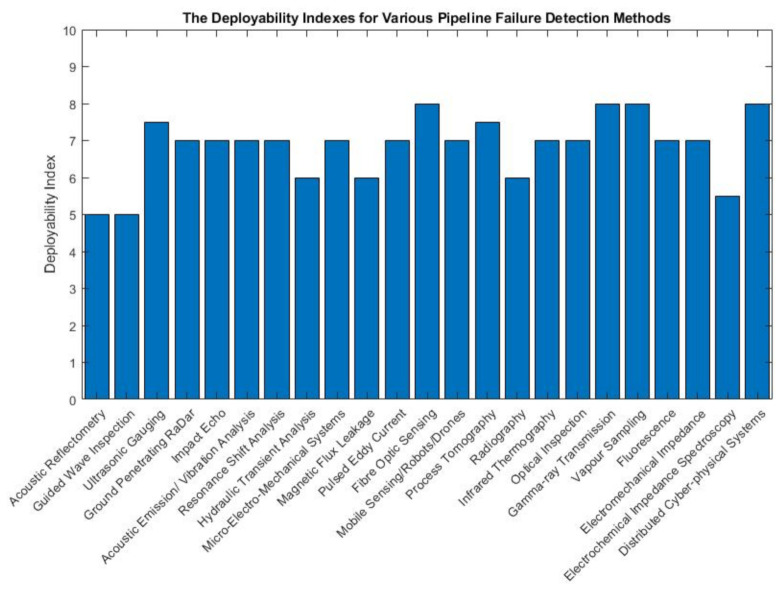
The deployability indexes for various pipeline failure detection methods.

**Figure 65:**
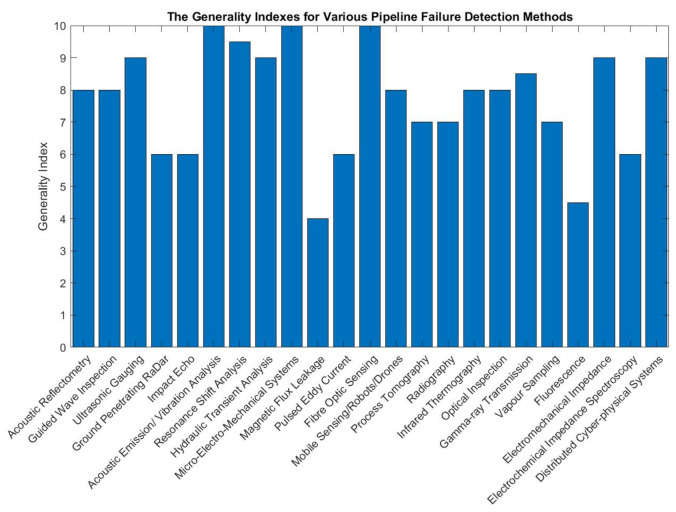
The generality indexes for various pipeline failure detection methods.

**Figure 66:**
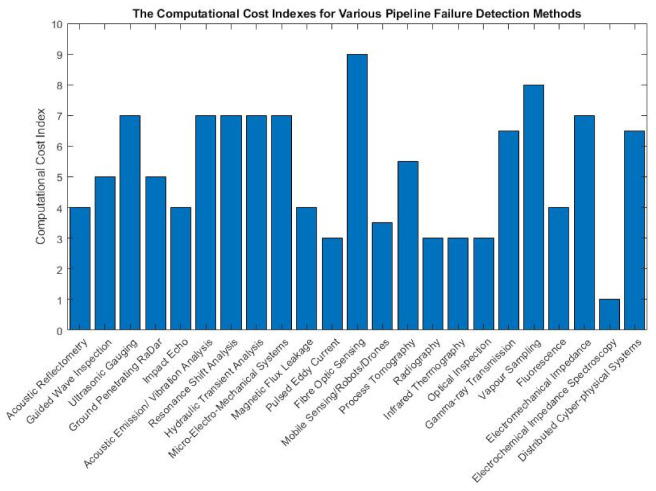
The computational cost indexes for various pipeline failure detection methods.

In this section, we revisit the suitability, unique features and the limitations of all the failure detection methods for pipeline networks that were discussed in the previous sections. We have also put together arrays of metrics in the form of scoring matrices as shown in Table 2, Table 3 and Table 4, which can be used, respectively, to define the levels of deployability, generality and computational cost of a detection method. The aspect of deployability of an inspection method considers the intensity of the effort needed to implement the method in industrial pipeline networks. The generality of an inspection method is measured based on the level of human intervention required to adjust the hardware specifications and software parameters to cater to pipeline networks of various operating environments. The computational cost takes into account elements, such as the types of the algorithms used, the processing power and the number of sensor nodes. The indexes of deployability, the system generality and the computational costs of the methods, calculated by summing up the scores of the metrics in the scoring matrices, are shown in the form of bar charts in Figure 64, Figure 65 and Figure 66.

Acoustic reflectometry is widely utilised for the detection of various pipeline defects. It is proven to be effective in the detection of leakages, cracks and weld defects. The use of audible sound waves in reflectometry is a popular choice for the detection of large blockage and severe leakage events. Audible sound waves, which have wavelengths longer than 1.9 cm, have a wider scanning area which are suitable for the detection of large defects, such as full blockages or partial blockages in pipelines with large diameters. However, audible waves have long wavelengths that limit the resolution of the reflected signals of the transmitted waves, resulting in the suppression of the potential for the 2D or 3D reconstruction of pipeline defects. Therefore, for applications that require the detailing of defects with sub centimetre dimensions, ultrasonic waves are a better candidate, with significantly shorter sub-millimetre wavelengths, owing to their higher frequencies in the megahertz range. However, most single-unit ultrasonic transducers do not work well for the detection of defects in large pipelines due to the narrower scanning area of the ultrasonic waves compared to that of the audible sound waves. In order to overcome the limitation in the scanning area of an individual transducer unit while leveraging the sub-millimetre measurement accuracies of the ultrasonic waves, multiple ultrasonic transducers can be arranged in the form of a guided wave transducer ring or phased array to produce a resultant ultrasonic beam with higher energy and directivity. This development has essentially led to an increase in the popularity of the employment of ultrasonic waves, surpassing that of audible sound waves, for the non-destructive testing of pipelines. The development of ultrasonic phased arrays has also made the inspection of girth welds possible since multiple ultrasonic transducers can be operated simultaneously to produce a resultant high-energy, high-directivity and steerable ultrasonic beam that can penetrate through the wall of a pipeline to measure different layers of a girth weld to detect the presence of defects, if any. One notable weakness of the guided wave transducer ring or phased array if used for the inspection of underground pipelines is the need to excavate the pipelines before the inspection can be carried out. The need for human intervention and service disruptions during the inspection of pipelines for both the acoustic reflectometry and guided wave inspection is reflected in the lowest deployability indexes of these methods among the rest as shown in Figure 64.

The narrow scanning area of an ultrasonic beam, despite being a weakness in reflectometry, was leveraged in ultrasonic gauging to measure the thickness of the walls of pipelines with micrometre accuracies by employing the time-of-flight principle. Human intervention and the disruption of services are not required during the inspection of pipelines using ultrasonic gauging. Since the ultrasonic gauge is mounted externally on the external wall of a pipeline, the technology can be applied in any types of pipeline facilities: building materials and types of substances carried, with the possibility of a challenging installation environment for underground pipelines. Therefore, the ultrasonic gauging technology is presented as one of the methods that have high deployability and generality indexes as shown in Figure 64 and Figure 65. However, ultrasonic gauging performs only localised measurements. Therefore, in order to create a thickness profile of the pipelines in a large network, the installation of multiple ultrasonic transducers is required at accessible locations. Data interpolation has to be implemented to estimate the thickness values in segments of pipelines that are not physically covered by the transducers. In conjunction with the growing interest in the use of in-pipe robots, there is an increasing demand for the miniaturisation of sensors that are both portable and capable of contactless sensing. Acoustic resonance technology (ART), which employs a wide-band ultrasonic beam instead of a narrow-band ultrasonic beam in standard reflectometry methods, was experimented with to measure the state of corrosion of the internal wall of a pipeline using the acoustic resonance technology (ART) has also been experimented. The ART transmits a wide-band ultrasonic beam and subsequently measures the amplitudes of the reflected waves at various resonant frequencies to determine the thickness of the wall.

Ground-penetrating radar (GPR) and impact echo (IE) which also operate based on the principle of reflectometry are widely employed in the detection of leakages in underground pipeline plants. Unlike acoustic reflectometry that is performed locally in or on the external surface of a pipeline, the signals from the excitation sources of GPR and IE are transmitted across the soil from above the ground. Due to the attenuation of the signals by the soil and also the low directivity of the excitation signals, the granularity of the reflected signals is often too low for accurate deductions about the presence of defects unless the defects are severe such as leakages where a huge amount of water loss is ongoing. Despite these drawbacks in terms of the resolution of the reflected signals, GPR and IE are non-intrusive and can be implemented easily using human-operated tools. These technologies are, therefore, suitable for small-scale pipeline plants where the human intervention required is minimal. Despite having various testimonials providing evidence about the effectiveness of reflectometry methods in the detection of wide defects, it is important to note that these reflectometry methods require tools that exist in the form of hardware with large form factors that are difficult to adopt for wireless sensor networks. The GPR and IE methods, being constrained only to the capability of detecting severe leakages occurring in underground pipelines, are among the methods with low generality indexes as shown in Figure 65.

As the use of wireless sensor networks in pipeline failure detection has increasing relevance in conjunction with the fast development in embedded systems, technologies based on the analysis of acoustic emissions, vibrations, resonant frequencies and hydraulic parameters have gained widespread research interest since 2010. These technologies employ simple sensors that are affordable and readily available such as the pressure sensors, flow sensors, acoustic sensors and micro-electro-mechanical sensors. Accelerometers and piezoelectric pressure sensors are examples of the micro-electro-mechanical sensors that have undergone drastic miniaturisation, making them the perfect candidates for employment in wireless sensor networks. One significant challenge that may be faced in the processing of the data from these sensors is the noise present in the readings that could affect the reliability of the sensing system, especially in the detection of small defects. The noise is caused by the presence of external disturbances taking place amidst the fast polling process used by the sensors to collect real-time readings from their surrounding environments. Therefore, before the data are fed to the processing pipeline, these data are first denoised using various types of wavelet transforms. However, caution should be given to the aggressiveness of the noise reduction since there is a tendency for these transforms to remove high-frequency components attributed to small defects such as tiny leakages. For data consisting of hydraulic parameters, these data, after being denoised, are often being processed using the transient analysis or the gradient analysis to identify the locations and the severity of the defects in a pipeline network. Data from motion or acoustic-based sensors are normally processed using cross-correlation techniques to accurately localise the defects. Supervised machine learning algorithms, such as the support vector machines, were also employed to predict classify the types and the severity of the defects based on features extracted from the signals measured by the sensors. However, methods relying on sensor networks require an optimal distribution of sensors in terms of the placement and quantity. A high-capacity pipeline plant with a large number of pipelines may require a large distribution of sensors that may incur a high cost of deployment and manpower for the installation of sensors. The reliance of wireless sensor networks on a large number of sensors and various computational algorithms contributes to the high computational cost indexes as shown in Figure 66. However, most of these sensor networks have high generality indexes as shown in Figure 65 since they are capable of detecting multiple types of defects in various pipeline environments.

The rising concern about the difficulty in retrofitting sensors to aged pipelines and the amount of human intervention needed for the maintenance of these sensors has brought about the use of magnetic flux leakage (MFL) pipeline inspection gauges (PIGs) in the detection of leakages, cracks and corrosion in long metallic pipelines. MFL PIGs are able to travel long distances in a pipeline network to monitor the structural integrity of the pipelines. Since no excavation activities and on-site installation of sensors are required for the employment of MFL PIG, the inspection time and the deployment cost for MFL PIGs are significantly lower than those required by acoustic reflectometry and sensor networks. Unlike reflectometry methods that rely on the analyses of reflected signals, the sensors on the PIGs measure the strength of the magnetic field lines along the internal wall of a pipeline directly. However, since the wall of a pipeline is constantly under magnetic saturation due to the strong magnetic fields produced by the magnets on the PIGs, there is therefore a reduced sensitivity of the sensors towards small events of magnetic flux leakage due to minor discontinuities on the internal wall of a pipeline. The level of magnetic saturation in the wall of a pipeline cannot be compromised since the saturation is key to accentuate the effect of magnetic flux leakage at the locations of cracks or leakages. Achieving high magnetic saturation in pipelines with very thick walls but smaller diameters is practically challenging since the size of the magnets required will be proportionally large and heavy. The large form factor of a MFL PIG may also cause mechanical damage to aged pipelines. Due to the employment of the magnetisation of metal, MFL PIGs can only be used in the inspection of metallic pipelines. This limitation along with the dimensional constraints of MFL PIGs are the underlying reasons why the MFL method has the lowest generality indexes among others as shown in Figure 65. The pulsed eddy current (PEC) technology which operates based on the principle of electromagnetism has eliminated the limitations present in conventional MFL PIGs by having a device, which has a significantly smaller form factor and does not require the use of magnetic saturation.

Fibre optic sensing (FOS) are futuristic replacements for the existing shape sensing technologies such as the cameras, RaDar and LiDAR. This means of sensing is capable of identifying the structural changes of a pipeline by measuring the changes in the strain of the optical fibre wrapped around the circumference of the pipeline. The changes in the strain of an optical fibre are determined using an interrogator unit that is relatively more expensive compared to standard industrial-grade sensors. Despite its expensive cost, one interrogator unit can serve one or more optical fibres of several kilometres in length. Therefore, despite having the highest computational cost index as shown in Figure 66, FOS have excellent deployability and generality scores as shown in Figure 64 and Figure 65. However, the setback in terms of the deployability of FOS becomes increasingly significant as the number and lengths of the pipelines increase since the installation of the optical fibres in a high-capacity pipeline plant requires high-level precision and significant manpower. The deployment of FOS becomes more challenging for underground pipelines since the access to the pipelines is limited by the locations of the inspection chambers. Nonetheless, FOS offers high sub-metre to several metres of spatial resolution depending on the operating wavelength of the light source. The optical fibres are also immune to disturbances from external electromagnetic fields since light pulses are used instead of electric current for the carriage of data.

The maturity of embedded systems in sensing has witnessed the proliferation of mobile sensing devices, robots and drones in pipeline defect monitoring. Like the MFL PIGs, the use of robots eliminates the need to rely on large sensor networks to monitor large pipeline plants. Instead, small sensor modules are carried and driven in the internal environment of a pipeline network to carry out all the required sensing activities. This effort reduces the maintenance workload required since the number of sensors required for the inspection process has been reduced significantly. Therefore, the availability of robots for in-pipe monitoring has also created the opportunity for revolutionised sensing technology such as the membrane-deflecting mechanism used in the PipeGuard robot by MIT for the detection of pressure changes caused by the presence of leakages along the internal wall of a pipeline. However, there has always been a notion about the intrusiveness of in-pipe robots that may cause disruptions to normal pipeline operations. This notion can be rectified by choosing the right type of robots, sensing technologies suitable for the internal environment of the pipeline network and an optimised monitoring schedule. The PipeGuard, for example, was able to flow along with the water in the pipelines to detect leakages without disrupting the operations of the water pipeline network. Certain internal pipeline environments, such as the operating internal environment of pneumatic waste conveyance pipelines, are not suitable for the use of inspection robots due to the high-speed transportation of solid waste in the pipelines. Such scenarios require a monitoring schedule to make full use of the non-operating period of the pipelines for the robots to perform inspection. The highlighted advantages of the mobile sensing devices, robots and drones contribute to the merit in the deployability, generality and computational cost indexes shown in Figure 64, Figure 65 and Figure 66 respectively.

As three-dimensional reconstruction of civil structures became increasingly mainstream especially for the inspection of structural integrity, process tomography has undergone rapid development especially in the domain of blockage and corrosion detection. Two most common inspection technologies under process tomography are the electrical capacitance tomography (ECT) and the magnetic induction tomography (MIT). Despite scoring a relatively high computational cost index due to the data processing techniques required for three-dimension image reconstruction, these technologies have moderate to high deployability and generality cost indexes making process tomography an appealing option among all other methods. ECT has shown massive potential for the detection and quantitative characterisation of partial blockage in the form of wax deposit in non-metallic pipelines. Not only ECT is able to validate the presence of blockage, it is able to accurately quantify the percentage of the cross-sectional coverage of the blockage with approximately 90% certainty in most scenarios. However, the working principle of ECT that relies on the thickness of dielectric insulators for the measurement of capacitance, which is also possible with non-metallic pipelines, has limited the widespread employment of this method. However, as polyvinyl chloride (PVC) pipelines become increasingly common especially in the transportation of water, ECT will continue to remain relevant. MIT is another notable approach that is capable of detecting metal loss events in metallic pipelines by measuring the changes in the electromagnetic properties caused by the reduction in the thickness of walls of the pipelines. The design of the MIT measurement system that takes the form of a uniformly distributed coils along the circumference of a pipeline is essentially an enhanced pulsed eddy current (PEC) method. Unlike the conventional PEC method that involves only one unit of probe, the MIT measurement system allows an array of probes to work simultaneously to map the thickness of the wall of a pipeline radially. Owing to the underlying technology employed in MIT, a MIT measurement system can only be used for the monitoring of metallic pipelines. The collection of data using the ECT and MIT measurement systems is just the initial stage of process tomography since data processing such as linear back-projection and narrow-band low fast filtering algorithms have to be implemented to reconstruct the three-dimensional model of the monitored pipeline. It is also important to note that careful attention is required during the assembly of the testing setup for tomography since the sensors have to be mounted precisely for reliable and accurate quantitative evaluation.

Radiography and active infrared thermography have shown immense potential for the quantitative evaluation of corrosion in pipelines. The passive infrared thermography, despite having lower potential for corrosion monitoring due to absence of heat control, remains a viable and deployable option for the qualitative characterisation of corrosion since miniaturised thermal cameras such as the hardware solutions provided by FLIR Lepton are readily available. The radiography and active infrared thermography, on the other hand, have limited deployability in the industry due to the bulkiness of the excitation sources and the sensing devices required by these sensing technologies. Like audible sound reflectometry and ultrasonic gauging, both radiography and active infrared thermography are only capable of localised inspection of corrosion that may not be favourable for the monitoring of large pipeline plants. The ultrasonic gauging tool has a much smaller scale factor compared to the set of tools required for radiography and thermography, making it suitable for bulk installation at multiple locations of a pipeline network. Despite the challenges in deploying these technologies in industrial pipeline plants, radiography stands out by being able to utilise simple image processing techniques to produce images of corrosion and weld defects of high granularity and clarity. The active infrared thermography is treated as the four-leaf clover that fulfils the requirements of a futuristic sensing method by being capable of direct measurement of metal loss, real-time monitoring, no or minimal disruption to pipeline services and contactless monitoring. The continuous effort to make radiography and active thermography relevant with pipeline inspection in the industry through miniaturisation or the reduction in the bulkiness of the sensing tools to further increase the deployability and generality will result in the application of these technologies in any emerging state-of-the-art detection strategies. The potential of these technologies can also be testified by the high deployability indexes, high generality indexes and low computational indexes relative to those scored by other methods or technologies.

Gamma-ray transmission is used for the detection of the deposits on the wall of a pipeline. While the deduction of the thickness of the deposits is computationally straightforward due to the simple inverse proportionality between the intensity of the transmitted gamma-ray and the thickness of the deposits, the use of gamma-ray poses safety hazard which therefore limits the deployability of the gamma-ray transducers in the public environment, The transducers for the gamma-rays, like the sensing system for radiography, thermography and ultrasonic gauging, are only capable of localised monitoring. Vapour sampling evaluates the presence of leakages in gas pipelines by evaluating the gaseous concentration of the ambience of the pipelines. Since the evaluation is performed using sensing tubes that are installed adjacent to the gas pipelines, the retrofitting of these tubes to existing pipeline networks may be challenging if spatial constraints exist. The detection of fluorescence signatures from hydrocarbon compounds leaked from pipelines carrying petroleum is a viable option for the quantitative characterisation of leakage events. The type of fluorosensors may differ greatly for different hydrocarbon compounds since fluorescence can only be induced if an excitation laser with the right wavelength is used. Vapour sampling and fluorescence are prone to be affected by the element of uncertainty present in the operating principles of their respective sensing systems. For vapour sampling, the random diffusion of particles may cause the leaked gas to be dispersed quickly, resulting in the occurrence of false-negative detection scenarios. In fluorescence sensing, the erratic photoelectric behaviour of the chemical compounds and the reaction of these compounds with the environment may affect the consistency of the inspection results.

The measurement of the electromechanical impedance (EMI) of a pipeline allows the evaluation of the structural integrity of the pipeline. EMI uses piezoelectric transducer to induce high-frequency vibrations in a structure before measuring the impedance at the interface between the piezoelectric material and the structure. The variations in the impedance measured are used as the inputs for the calculation of the damage index which provides information about the severity of the structural damage of a pipeline. Due to the localised coverage of the EMI sensors, the deployability and scalability of the EMI method depend on the number of EMI sensors required. In the industry such as in the evaluation of the integrity of the engine housing of jet planes, the EMI sensors are only installed at locations that are prone to damage. The electrochemical impedance spectroscopy (EIS) is a dated approach for the offline evaluation of the corrosion states of metal structures. By measuring the amount of current flowing through a metal specimen as the voltage across the specimen is varied, the state of corrosion of the specimen can be determined by correlating the change in electrical impedance with the corrosion rate of the specimen. Despite being able to accurately determine the state of corrosion of a metal specimen, EIS is a laboratory approach that is slow in producing results compared to the real-time monitoring solutions in the form of wireless sensor networks.

The modelling approaches help to determine the severity of a particular defect in between two in-line inspections. In-line inspection using MFL PIGs, in-pipe robots and guided ultrasonic transducer rings are conducted periodically due to the unfavourable level of human intervention and number of disruptions to the services of the pipeline networks. Corrosion growth modelling allows the progression of the corrosion states in a pipeline network to be determined based on the results from the previous in-line inspection. Various computer algorithms such as Monte Carlo simulations, stochastic modelling and dynamic bayesian networks have been employed in the modelling process. There is a lot of room for the innovation of new strategies to model corrosion more accurately. The type of modelling is data-centric the since the quantitative progress of corrosion defects can only be determined with existing knowledge about the corrosion states in a pipeline network that is available from the results of previous in-line inspections.

With the proliferation of efficient embedded systems in the industry, distributed cyber-physical systems in the form wireless sensor networks have become increasingly practical, especially for long-term continuous sensing especially for the monitoring of flow of fluid in pipelines to detect the presence of leakages. This can be testified by the high deployability and generality indexes scored. Although the signal or data processing algorithms are computationally light since embedded hardware is used in most cyber-physical systems, however, the number of sensors required to achieve the sensing objective is large, resulting in a high computational index score. Apart from MEMS which have undergone sufficient miniaturisation and improvement in reliability over the years, the form factors of most of the state-of-the-art sensing technologies especially those falling under the acoustic reflectometry and wave guided inspection categories are still relatively large and in need of human intervention. Thus, there is still room for further advancement of distributed cyber-physical systems in the pipeline inspection domain. There is a high possibility that this advancement will take place rapidly since wireless sensor networks are being adopted in various industries for asset monitoring.

There has been a low general interest in works on the fusion of existing state-of-the-art pipeline failure detection methods for the simultaneous detection of multiple defect types. In [163], a probabilistic model based on the dynamic bayesian networks, combining both distributed acoustic sensing (DAS) and internal leak detection (ILD), was proposed to detect and localise leakages in pipelines. In [164], the combination of the radial basis function (RBF) neural networks and data fusion based on Dempster-Shafer (DS) theory was used to identify leakage signals and locations of leakages respectively. By putting together two or more methods, where each of the methods has at least one strength that can overcome one or more weaknesses present in another method, the effectiveness and confidence level of defect detection can be significantly increased. Moreover, there is a potential for these hybrid solutions to cater for multiple defects since different methods could be suited for the detection of different types of defects.

## 4. Conclusions

In this paper, a taxonomy of detection methods for blockages, leakages, cracks, corrosion and weld defects in pipeline networks were developed and discussed. This effort is relevant to the existing need to identify the state-of-the-art pipeline failure detection methods or technologies that could be implemented to evaluate the structural integrity of various types of pipeline networks. For each detection method, the approaches used for signal analysis, data processing and defect classification were covered. Validated results from real-life experiments and computer simulations were also reviewed to provide a more comprehensive visualisation of the expected outcomes for different detection methods.

The strengths and weaknesses of all the methods discussed in Section 2 were summarised and comparatively analysed in Section 3. Figure 64, Figure 65 and Figure 66 show the variations in terms of the deployability, the generality and the computational cost for various pipeline failure detection methods. While every state-of-the-art methods feature their individual outstanding strengths, inconsistency in the performance of detection still exists due to the presence of hardware and software limitations. In order to optimise the performance of a pipeline defect detection strategy, multiple state-of-the-art defect detection methods can be combined either physically or in the form of data fusion to both improve the performance of the detection of a single type of defect and to cater for the detection of multiple types of defects.

The findings of this paper will lead to future works aimed at devising a new reliable failure detection platform that is adaptable for different pipeline types and operating environments. We are currently conducting experiments to test the feasibility of the measurement of metal loss in pipelines using various remote sensing technologies. The feasibility of these technologies is being tested separately to identify their individual potential strengths and weaknesses. The fusion of data from these individual technologies will be implemented in tandem with the use of an in-pipe robotic carrier to generate a complete quantitative degradation profile of a pipeline network consisting of the thickness of the walls of the pipelines and the patterns of metal loss such as grooves and pittings. The potential of corrosion sensing using depth camera imaging and radio frequency (RF) sensing will also be evaluated both experimentally and in computer simulations. Subsequently, the effectiveness of these sensing strategies will be benchmarked against the performance of the existing state-of-the-art defect detection technologies.

In order to reduce the impact of the limitations of a robotic based inspection system, such as the need for human intervention and the possible service disruptions during an inspection, the data collected during the inspection will be used to develop a corrosion growth model based on machine learning. The corrosion growth model provides a predictive platform for the forecast of future metal loss events. Accurate predictions of the progression of corrosion in a pipeline network potentially lead to the possibility of preventive maintenance through the optimisation of the maintenance regimes. The occurrence of disruptive failures often results in service downtime of a pipeline network due to the need for fault identification or major repair jobs. Preventive maintenance helps to reduce the probability of disruptive failures. The final outcome of our research will be translated into practical means to optimise maintenance regimes and minimise service disruptions of the pneumatic waste conveyance system (PWCS) and water pipeline networks in Singapore.

## Figures and Tables

**Table 1:** The suitability of existing non-destructive methods for the detection of different pipeline defects.

Failure Detection Methods	Defect Type					Key Aspects/Data or Signal Processing Techniques	References
	Blockage	Leakage	Crack	Corrosion	Weld Defect		
Acoustic Reflectometry	✓	✓	✓	✓	✓	Time-of-flight; phase change; power reflection ratio; spectral analysis; synthetic aperture radar; acoustic resonance technology; ultrasonic phased array	[6,7,8,42,43,44,45,46]
Guided Wave Inspection	✓	✓	✓	✓		Time-of-flight; ultrasonic transducer ring; phase change; spectral analysis; transmission/reflection coefficient analysis; non-linear modulation; guided microwave inspection	[9,47,48,49,50,51,52,53,54]
Ultrasonic Gauging				✓	✓	Time-of-flight; time-series cross-correlation; Gaussian model-based estimation; temperature compensation	[55,56,57,58,59]
Ground Penetrating RaDAR (GPR)		✓				Back-projection; back-propagation; GPR-camera fusion; Bayern approximation	[2,60,61,62,63,64,65]
Impact Echo (IE)		✓	✓			Sustained duration; resonance analysis; correction factor validation; Edge reflection analysis; noise removal	[19,66,67,68,69,70]
Acoustic Emission (AE)/ Vibration Analysis	✓	✓	✓		✓	Frequency analysis; vibrational amplitude and fluid transient analysis; time-difference cross-correlation; wavelet entropy analysis; machine learning classification	[1,9,10,11,30,38,71,72]
Resonance Shift Analysis	✓	✓	✓			System resonant frequency, amplitude, quality factor and bandwidth shifts analysis	[73,74,75,76,77,78]
Hydraulic Transient Analysis	✓	✓				Finite difference modelling; linear estimator; short duration transient test; fluid transient harmonic damping analysis; negative pressure method; gradient method; sequential probability ratio technique; wavelet transforms	[12,13,31,79,80,81,82,83,84,85,86]
Micro-Electro-Mechanical System (MEMS)		✓				Piezoelectric sensors; capacitive sensors	[87,88,89,90,91,92,93,94]
Magnetic Flux Leakage (MFL)		✓	✓		✓	Amplitude of MFL vs. length/width of defect; machine learning classification; decoupling algorithm	[4,16,17,95,96,97,98,99,100,101,102]
Pulsed Eddy Current (PEC)				✓		Electrical conductance analysis; magnetic permeability analysis; differential probe	[103,104,105,106,107]
Fibre Optic Sensing		✓	✓	✓		Spectral analysis; hoop strain analysis	[5,108,109,110,111,112,113,114,115,116]
Mobile Sensing/Robots/Drones	✓	✓	✓	✓		Pressure gradient analysis; pipeline inspection gauge (PIG); driving mechanisms; manoeuvrability	[3,4,27,28,95,108,117,118,119,120,121,122]
Process Tomography	✓	✓	✓	✓		Electrical capacitance measurement; magnetic induction measurement; ultrasonic measurement; image reconstruction; linear back-projection; narrow-band pass filtering	[32,33,123,124,125]
Radiography				✓	✓	Pixel intensity vs. pipe thickness; double wall double image technique; machine learning classification	[126,127,128,129]
Infrared Thermography		✓		✓		Thermal emissivity; thermal capacity; pulsed thermography; step heating thermography; lock-in thermography; spectral analysis,	[3,18,130,131,132]
Optical Inspection	✓	✓	✓	✓		Light intensity of image vs. surface condition/texture	[133,134,135,136]
Gamma-ray Transmission	✓					Transmission intensity vs. pipe thickness	[137]
Vapour Sampling		✓				Vapour sensing tube	[138]
Fluorescence		✓				Wavelength of fluorescence vs. type of spillage	[139,140,141]
Electromechanical Impedance (EMI)		✓	✓			EMI vs. structural integrity; piezoelectric-induced vibration; measurement of electrical impedance	[142,143,144,145,146,147,148]
Electrochemical Impedance Spectroscopy (EIS)				✓		Impedance measurement; polarisation resistance vs. corrosion rate	[149,150]
Corrosion Growth Modelling				✓		Stochastic corrosion model; Monte Carlo simulation	[151,152,153,154,155]
Distributed Cyber-physical Systems		✓				Wireless sensor networks; pressure and acoustic data analysis; post-order transversal algorithm; WaterBox; search algorithm; machine learning	[25,34,35,40,156,157,158,159,160]

**Table 2:** Scoring matrix for deployability.

**Number of Sensing Devices**	Multiple Sensing Devices	One Sensing Device	
	0	1	
**Human Intervention during Inspection**	Full	Partial	None Required
	0	1	2
**On-site installation**	Intrusive	Non-intrusive	None Required
	0	1	2
**Maintenance**	Disruptive	Non-disruptive	
	0	1	
**Disruptions during Inspection**	Full	Partial	None
	0	1	2
**Inspection Type**	Offline	Periodical	Real-time
	0	1	2

**Table 3:** The deployability indexes for various pipeline failure detection technologies.

**Type of Defects**	One Type	Multiple Types	
	0	1	
**Building Materials of Pipelines**	One Type	Multiple Types	
	0	1	
**Substances Carried by Pipelines**	One Type	Multiple Types	
	0	1	
**Operating Environment**	Underground or overground	Underground and overground	
	0	1	
**Diameter of Pipelines**	Fixed	Variable	No constraints
	0	1	2
**Software Parameters**	Exhaustive	Minimal tuning or none required	
	0	1	
**Hardware Duplication**	Difficult to duplicate	Can be duplicated with minimal or no changes required	
	0	1	
**Hardware Portability**	Non-installable	Temporary installation	Permanent installation
	0	1	2

**Table 4:** Scoring matrix for computational cost.

**Inspection Type**	Offline	Periodical	Real-time
	0	1	2
**Computational Platform**	Embedded systems	Dedicated computing software	Advanced computing platforms
	0	1	2
**Number of Sensor Nodes**	Single-node operation	Multiple nodes in a single sensing device	Distributed sensor networks
	0	1	2
**Processing Mode**	Offline	Online with delays	Near real-time
	0	1	2
**Type(s) of Algorithms**	Mathematical Formulations/ Modelling	Signal Processing or Image Processing and/ or Mathematical Formulations/ Modelling	Signal Processing and Image Processing and/ or Artificial intelligence/ Mathematical Formulations/ Modelling
	0	1	2

## Data Availability

Not applicable.
